# Structural insights into the function of type VI secretion system TssA subunits

**DOI:** 10.1038/s41467-018-07247-1

**Published:** 2018-11-12

**Authors:** Samuel R. Dix, Hayley J. Owen, Ruyue Sun, Asma Ahmad, Sravanthi Shastri, Helena L. Spiewak, Daniel J. Mosby, Matthew J. Harris, Sarah L. Batters, Thomas A. Brooker, Svetomir B. Tzokov, Svetlana E. Sedelnikova, Patrick J. Baker, Per A. Bullough, David W. Rice, Mark S. Thomas

**Affiliations:** 1 Department of Molecular Biology and Biotechnology, Krebs Institute, University of Sheffield, Sheffield, S10 2TN UK; 2 Department of Infection, Immunity and Cardiovascular Disease, University of Sheffield Medical School, Beech Hill Road, Sheffield, S10 2RX UK; 3 Present Address: Department of Chemistry, King’s College London, Britannia House, London, SE1 1DB UK; 4 Northern Genetics Service, The Newcastle upon Tyne Hospitals NHS Foundation Trust, Institute of Genetic Medicine, International Centre for Life, Newcastle upon Tyne, NE1 3BZ UK

## Abstract

The type VI secretion system (T6SS) is a multi-protein complex that injects bacterial effector proteins into target cells. It is composed of a cell membrane complex anchored to a contractile bacteriophage tail-like apparatus consisting of a sharpened tube that is ejected by the contraction of a sheath against a baseplate. We present structural and biochemical studies on TssA subunits from two different T6SSs that reveal radically different quaternary structures in comparison to the dodecameric *E. coli* TssA that arise from differences in their C-terminal sequences. Despite this, the different TssAs retain equivalent interactions with other components of the complex and position their highly conserved N-terminal ImpA_N domain at the same radius from the centre of the sheath as a result of their distinct domain architectures, which includes additional spacer domains and highly mobile interdomain linkers. Together, these variations allow these distinct TssAs to perform a similar function in the complex.

## Abstract (web summary)

TssA is an important component of the bacterial type VI secretion system (T6SS). Here, Dix et al. integrate structural, phylogenetic and functional analysis of the TssA subunits, providing new insights into their role in T6SS assembly and function.

## Introduction

Contractile bacteriophages of the family *Myoviridae* (i.e. T4), R-type pyocins and the type VI secretion system (T6SS) of Gram-negative bacteria are evolutionarily related nano-scale injection machines that puncture target cell membranes using a shared contraction mechanism^1–3^. These injection devices are comprised of an inner tube, surrounded by a contractile sheath, that are both assembled on a platform known as the baseplate. The inner tube is sharpened with spike proteins at the baseplate proximal end, which facilitates its penetration of target cells upon contraction of the sheath against the baseplate^2–5^.

The T6SS secretion machinery is formed from multiple copies of 12 core subunits (TssA-TssG, TssI-TssM) and a single PAAR tip protein^6–9^ and can be subdivided into two main components. One of these, the membrane complex, consists of 10 subunits each of TssJ, TssL, and TssM that assemble into a chamber-like structure with five-fold symmetry which serves to anchor the injection machinery at the cell envelope as well as providing an exit channel for translocated subunits and effectors^10–15^. The other component, the injection machinery, consists of two sub-complexes. One sub-complex consists of the inner tube, which is comprised of stacked hexameric rings of TssD (Hcp), capped by the trimeric hub protein, TssI (VgrG), and sharpened by the PAAR subunit, surrounded by repeating TssBC heterodimers that form the contractile sheath^1,3,5,16,17^. The latter consists of a six-start helix that possesses six-fold symmetry, giving a cogwheel-like appearance when viewed end-on^1,18–21^. Both the inner tube and sheath exhibit the same degree of helical twist thereby ensuring a six-fold symmetry match along the entire length of the tube-sheath complex^21^. The other sub-complex is the baseplate, which consists of TssE, TssF, TssG and TssK, and contains a central channel through which the sharpened inner tube passes upon contraction of the sheath^3,17,22–24^. The sheath is subsequently recycled by the AAA+ ATPase, TssH (ClpV)^1,18,25^.

Until recently, relatively little was known about the location and role of the TssA subunit within the T6SS complex. TssA subunits are enigmatic as they possess a conserved N-terminal region of unknown function, previously identified as ImpA_N (PFAM: PF06812^26^), whereas sequences located C-terminal to this region are highly divergent^6,27,28^. Consistent with this, phylogenetic analysis has suggested that the TssA family can be subdivided into three clades (TssA1, TssA2 and TssA3)^28^. The C-terminal regions of TssA1 and TssA2 have been shown to be required for assembly of these TssA subunits into higher order oligomers and both subunits are required for T6SS function^27,28^. However, the TssA3 subunit has not been previously investigated.

Recent studies on the TssA2 subunit of enteroaggregative *Escherichia coli* (EAEC), Ec042_4540, have provided structures for two of its putative three domains (the middle (Nt2) and the C-terminal domain (CTD)), leaving the structure of the highly conserved N-terminal domain (Nt1), yet to be determined. These structural studies showed that the CTD assembles into a dodecamer that resembles a six-pointed star. Further analysis showed that TssA2 interacts with components of the baseplate, inner tube, sheath and the T6SS membrane complex^27^. This led to the proposal of a capping model whereby TssA2 initially interacts with the core TssJLM membrane complex, thereby triggering baseplate recruitment. According to the model, TssA2 subsequently serves to coordinate the assembly of the inner tube and contractile sheath, during which it migrates away from the baseplate complex, remaining in contact with the distal end of the polymerising tube^27,29^. In a separate study, the TssA1 subunit of *Pseudomonas aeruginosa*, PA0082, was shown to form a ring-like complex of indeterminate stoichiometry and symmetry, and in contrast to TssA2, was proposed to serve as the T6SS counterpart of the phage T4 baseplate protein gp6, in a baseplate-associated model of TssA function^28^. However, in the absence of a high-resolution structure for TssA1 or TssA3, any relationship between their CTDs and the Nt2 and CTDs of TssA2 subunits are, as yet, unclear.

In this paper, we present evidence that there are only two main TssA clades (TssA1 and TssA2) and that each clade can be sub-divided into two distinct sub-clades, referred to here as A and B. We provide the first structure for any TssA1 subunit (TssA1^B^) to show that it is composed of two domains instead of three: the conserved N-terminal domain (Nt1), containing the ImpA_N region, that is tethered by a long linker to a distinct CTD that assembles into a ring containing 32 subunits with 16-fold symmetry. Furthermore, we present the first T6SS subunit interaction analysis of a TssA1^B^ member. We also provide the structure of an Nt2-CTD TssA2^A^ construct, demonstrating that 10 subunits assemble into a complex with distinct five-fold symmetry rather than the six-fold symmetry seen in EAEC TssA2^B^. The structure further reveals a striking range of conformational mobility of the TssA2^A^ Nt2 domain with respect to the CTD oligomer. Despite the differences in symmetry of TssAs belonging to the different clades, the similar subunit interaction network and disposition of their conserved domains suggests that they function by a related mechanism to coordinate inner tube/sheath assembly.

## Results

### Bioinformatic and proteolytic analysis of TssA family proteins

**Fig. 1:**
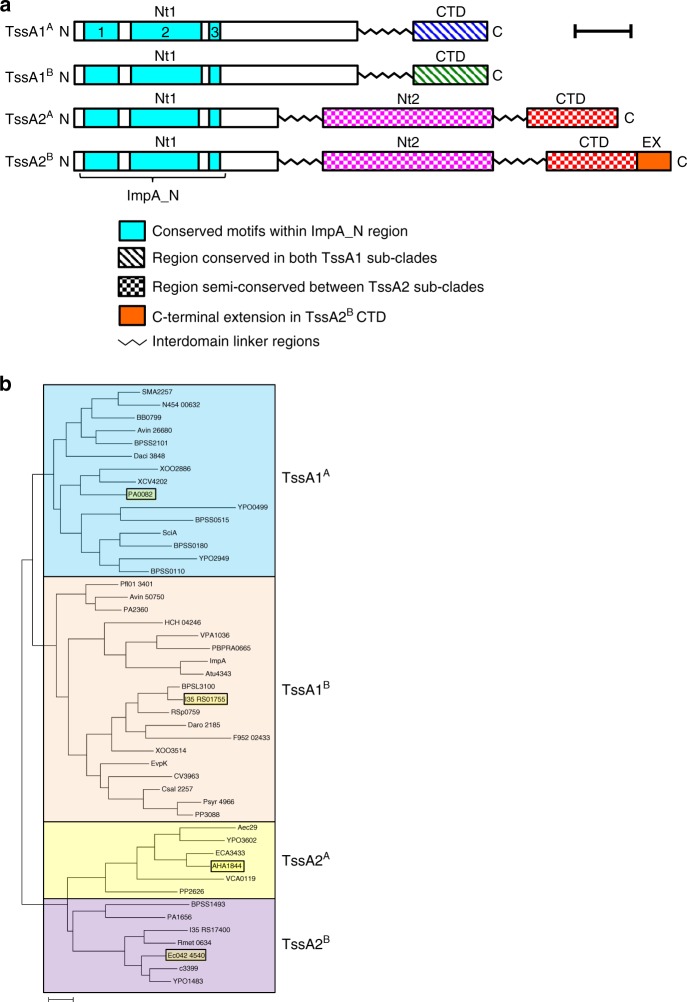
Phylogeny and domain organisation of TssA family proteins. **a** Proposed domain organisation of TssA1^A^, TssA1^B^, TssA2^A^ and TssA2^B^ subunits based on structural studies, secondary structure prediction and amino acid sequence alignment. The interdomain linkers comprise regions of variable length and amino acid sequence. The N-terminal conserved region, ImpA_N (comprised of three major regions of conservation, ImpA_N1-3), Nt1, Nt2, CTD and EX (C-terminal extension present in TssA2^B^ proteins) are indicated. Scale bar corresponds to 50 amino acids. **b** Maximum-likelihood phylogenetic tree from amino acid sequences of 47 TssA family members, showing the subdivision into three major clades as observed previously ^28^but with a revised nomenclature. The TssA2 clade is further divided into sub-clades A and B, based on patterns of sequence identity. TssA members discussed in detail in this study are highlighted in dark yellow. Scale bar represents 0.2 substitutions per site

Bioinformatic analysis suggests that all TssA subunits contain an N-terminal conserved region of ~120 amino acids (ImpA_N) composed of three shorter motifs of significant sequence similarity (ImpA_N1–3) (Fig. 1a and Supplementary Fig. 1). However, these proteins possess one of two distinct C-terminal regions based on amino acid sequence conservation, and are referred to here as TssA1 and TssA2 (Fig. 1a and Supplementary Fig. 2). In members of the TssA1 clade, ImpA_N forms part of a large N-terminal domain (Nt1) of ~250 amino acids which is connected by a central region of variable length (40–60 amino acids) and sequence to a conserved C-terminal region of 60–75 amino acids containing a signature EPxxP motif (Supplementary Fig. 2a). Phylogenetic analysis shows that TssA1 subunits fall into one or other of two sub-clades, referred to here as A and B (Fig. 1b). In TssA1^A^ members, such as *P. aeruginosa* PA0082 (Pa TssA1^A^), the EPxxP motif is EPSxP, whereas in TssA1^B^ members, such as *B. cenocepacia* I35_RS01755 (Bc TssA1^B^), an alternative EP(H/Q)SP motif is present (Supplementary Fig. 2a). TssA1^A^ corresponds to TssA1 in the previously proposed nomenclature^28^, whereas TssA1^B^ corresponds to TssA3. In comparison, TssA2 orthologues are longer (480–540 amino acids) and are predicted to contain three domains: Nt1 (including the ImpA_N region), a middle domain (Nt2) and CTD, each separated by regions of variable length and sequence (Fig. 1a and Supplementary Fig. 1 and 2b). However, our phylogenetic analysis reveals that TssA2 proteins are also subdivided into two sub-clades (Fig. 1b) with members of sub-clade B, represented by the EAEC TssA2 subunit (EAEC TssA2^B^) having an extension of 20–40 amino acids at the C-terminus compared to sub-clade A members, such as *A. hydrophila* AHA1844 (Ah TssA2^A^).

To provide support for the bioinformatics, analysis of protein fragments derived by either limited or adventitious proteolysis was used to identify putative domain boundaries. Analysis of Bc His_6_.TssA1^B^ degradation products suggested that TssA1^B^ possesses a large folded Nt1 domain (~31 kDa) connected to a small CTD (~8.5 kDa) by an interdomain linker of ~40 amino acids (Supplementary Fig. 3a, c, and d, Supplementary Table 1).

Products of limited proteolysis carried out on purified Ah His_6_.TssA2^A^ showed that residues in regions corresponding to positions R178-G190 and K378-E399, two regions of high sequence variability, were particularly susceptible to protease cleavage. This is consistent with the predicted three-domain architecture with approximate boundaries of M1-R178 for Nt1, D231-L374 for Nt2, and I402-K478 for the CTD (Supplementary Fig. 3b and e).

### Interactions of TssA1^B^ and its domains with other T6SS components

**Fig. 2:**
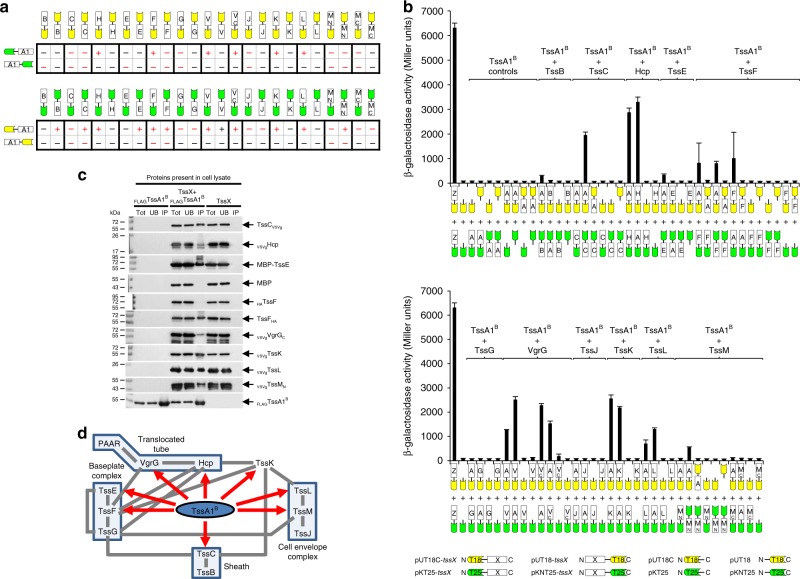
Interaction of TssA1^B^ with other T6SS subunits. **a** Two-hybrid analysis (maltose phenotypes). Hybrid proteins are represented by a green or yellow coloured motif representing the CyaA fragment (T25 and T18, respectively) linked to a white rectangle labelled according to the fused T6SS subunit, as shown in the key at the bottom of (**b**). T6SS subunits are indicated by a single letter corresponding to the suffix used in the Tss nomenclature (i.e. A1 corresponds to TssA1^B^, B corresponds to TssB etc) except for H (Hcp), V (VgrG) and V_C_ (VgrG core region). M_N_ and M_C_ represent the N-terminal cytoplasmic and C-terminal periplasmic regions of TssM, respectively. The efficiency of complementation of representative combinations (phenotypes shown in red font) were determined by β-galactosidase assay. **b** Two-hybrid analysis (β-galactosidase activity). Data is representative of three independent experiments (*n* = 3) performed in duplicate and values correspond to the mean ± standard deviation. Nomenclature as in (**a**). Z represents the Zip control. Values are presented in Supplementary Data 1. **c** Co-immunoprecipitation analysis. FLAG-tagged Bc TssA1^B^ and potential interacting T6SS subunits (TssX) tagged with the VSV-g or HA epitope tags, or with MBP, were co-expressed in *E. coli*. FLAG.TssA1^B^ was immunoprecipitated from cell lysates and recovered prey proteins (IP) were detected with the appropriate epitope antibody by western blotting. MBP was included as a control and was detected with MBP pAb. Proteins present in the cell lysate (Tot) and the unbound material (UB) were also analysed. Blots were also probed with FLAG mAb (an example of such a blot following co-expression of FLAG.TssA1^B^ and VSVg.TssM_N_ is shown in the bottom panel). Uncropped images of the blots are shown in Supplementary Fig. 4. **d** Bc TssA1^B^ interaction network. Interactions between Bc TssA1^B^ and other T6SS subunits are indicated by red arrows. Previously reported interactions are indicated by grey connectors. Note that VgrG and TssK have been shown to interact with the TssF-TssG complex^22^ but for simplicity are linked to TssF in this figure

The interactions of a TssA1^B^ subunit with other components of the T6SS have not been previously investigated. To address this, a two-hybrid analysis was performed. Complementation of CyaA function was assayed on maltose MacConkey indicator plates and by performing β-galactosidase assays, both of which indicated that TssA1^B^ may interact with TssC, Hcp, TssE, TssF, the conserved core region of VgrG (VgrG_C_), TssK, TssL and the N-terminal cytoplasmic region of TssM (TssM_N_) (Fig. 2a, b). To validate these results, co-IP experiments were performed using FLAG-tagged TssA1^B^ in pairwise combinations with other epitope-tagged subunits. The results supported the in vivo observations. Thus, epitope-tagged TssC, Hcp, TssF, VgrG_C_, TssK, TssL and TssM_N_ were all co-immunoprecipitated with FLAG.TssA1^B^ (Fig. 2c). Demonstration of an interaction between TssA1^B^ and TssE required solubilisation of TssE by fusion to the MBP solubility tag. In summary, the protein-protein interaction analysis suggests that TssA1^B^ makes contact with components of the baseplate, the membrane-anchoring complex, the inner tube and its cap, and the surrounding sheath (Fig. 2d). Two-hybrid analysis was then performed to investigate the role of the N- and C-terminal regions of TssA1^B^ in interactions with other T6SS subunits. This showed that TssA1^B^ CTD interacts with Hcp, TssF and VgrG (and also with VgrG_C_) but interactions with other subunits were not observed for the Nt1 domain (Supplementary Fig. 6).

### TssA1^B^ is an essential component of the T6SS

**Fig. 3:**
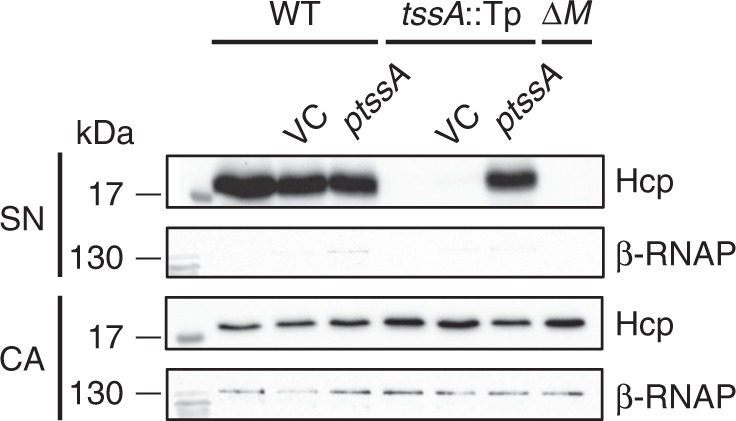
Effect of TssA1^B^ inactivation on T6SS secretion activity. Cell associated protein (CA) and spent supernatants (SN) from cultures of *B. cenocepacia* H111 (WT) and the isogenic *tssA1*^*B*^ mutant (*tssA*::Tp), each with or without pBBR1MCS (VC) or pBBR1MCS-tssA1^B^ (*ptssA*), were fractionated by SDS-PAGE, blotted onto PVDF membrane and probed with a Hcp pAb or a mAb specific for the RNAP β subunit (lysis control). Detection of bound antibodies was with HRP-conjugated secondary antibodies. The *tssM* mutant (Δ*M*) was included as a control as TssM has been shown to be an essential component of the *B. cenocepacia* T6SS^31^. Uncropped images of the blots are shown in Supplementary Fig. 5

P.a TssA1^A^, *V. cholerae* TssA2^A^ (VCA0119) and EAEC TssA2^B^ have been shown to be essential for T6SS activity^27,28,30^. Therefore, the requirement for TssA1^B^ for a fully functional T6SS was explored by assaying the ability of a *B. cenocepacia tssA1*^*B*^ mutant to secrete Hcp—an indicator of T6SS activity. The results showed that, unlike the wild-type strain, Hcp was absent from the culture supernatant of the *tssA1*^*B*^ mutant, but it was secreted when *tssA1*^*B*^ mutant bacteria contained a plasmid expressing *tssA1*^*B*^ (Fig. 3), thereby confirming the requirement for this subunit for a functional T6SS^31^.

### Structure determination of the TssA1^B^ Nt1 domain

**Fig. 4:**
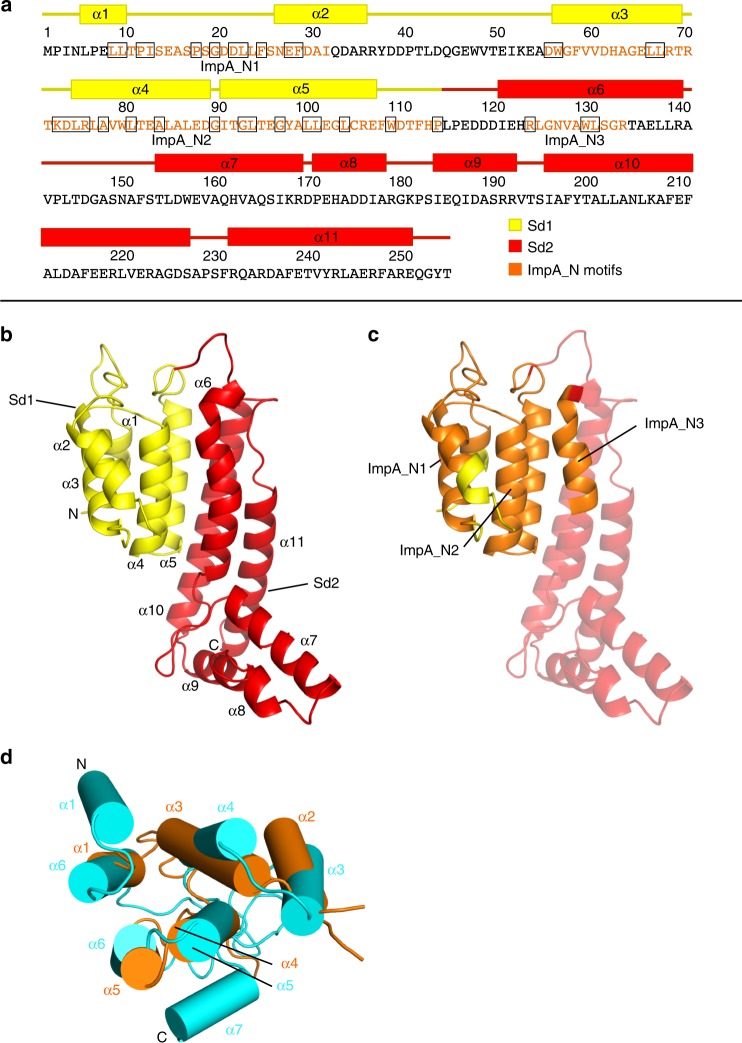
High-resolution structure of Bc TssA1^B^ Nt1 and its similarity to Ah TssA2 Nt2. **a** Bc TssA1^B^ Nt1 sequence annotated to show the elements of secondary structure. Location of α-helices are shown by coloured rectangles above the amino acid sequence with the region corresponding to Sd1 highlighted in yellow and Sd2 highlighted in red. The three ImpA_N motifs are indicated in orange font and conserved ImpA_N domain residues (see Supplementary Fig. 1) are shown in black boxes. **b** X-ray structure of Bc TssA1^B^ Nt1 (PDB: 6HS5 https://www.ebi.ac.uk/pdbe/entry/pdb/6hs5). The two subdomains, Sd1 and Sd2, are shown in yellow and red, respectively. **c** X-ray structure of Bc TssA1^B^ Nt1 represented as in (b) showing location of ImpA_N1-ImpA_N3 regions in orange. For clarity, the helices of Sd2 (α6-α11) located C-terminal to ImpA_N3 are made more transparent than those in Sd1. **d** Bc TssA1^B^ Sd1 (orange) superimposed on the Ah TssA2^A^ Nt2 domain (PDB: 6G7B https://www.ebi.ac.uk/pdbe/entry/pdb/6g7b; cyan) showing the close structural similarity of helices α1-α5 of Bc TssA1^B^ Nt1 (majority of ImpA_N) to helices α2-α6 of Ah TssA2^A^ Nt2^34^. α-helices are shown as cylinders

SEC and two-hybrid analysis of the overexpressed Bc TssA1^B^ Nt1 domain indicated that it is monomeric (Supplementary Fig. 7a, b). Analysis of the crystal structure of the TssA1^B^ Nt1 domain, determined to 1.80 Å (Supplementary Table 2), revealed a monomer composed of 11 α-helices that are organised into two subdomains, an N-terminal subdomain 1 (Sd1) connected to a larger subdomain 2 (Sd2) (Fig. 4a, b). Sd1 (M1-P113) is comprised of five antiparallel α-helices (α1–α5) and contains the ImpA_N1 (L8-I31) and the ImpA_N2 (D55–P113) motifs (Fig. 4a–c).

Sd2 of Bc TssA1^B^ (L114-Q251) contains six α-helices (α6–α11), of which the three longest (α6, α10 and α11) form a five-helix bundle with α4–α5 of Sd1. Residues from ImpA_N3 (R123–R133) (Fig. 4a–c) form the start of α6, a helix which includes a sharp kink between L124 and G125. The C-terminal helix of Sd2 (α11) terminates at Q251 and is thereafter followed by the connecting interdomain linker to the CTD. The sequence conservation in the ImpA_N region across the TssA clades (Supplementary Fig. 1) implies that all Nt1 domains are closely related and adopt a similar fold, which includes the entirety of Sd1, and the N-terminal part of the first helix of Sd2 of Bc TssA1^B^. Mapping of the 34 conserved residues in the ImpA_N region onto the structure of Nt1, shows that approximately two-thirds are involved in packing interactions in the core, with the remainder located predominantly at one end of the molecule in the loops between helices α1–α2, α3–α4, and α5–α6 (Fig. 4a, c).

### TssA1^B^ CTD forms a double-layered ring with D_16_ symmetry

**Fig. 5:**
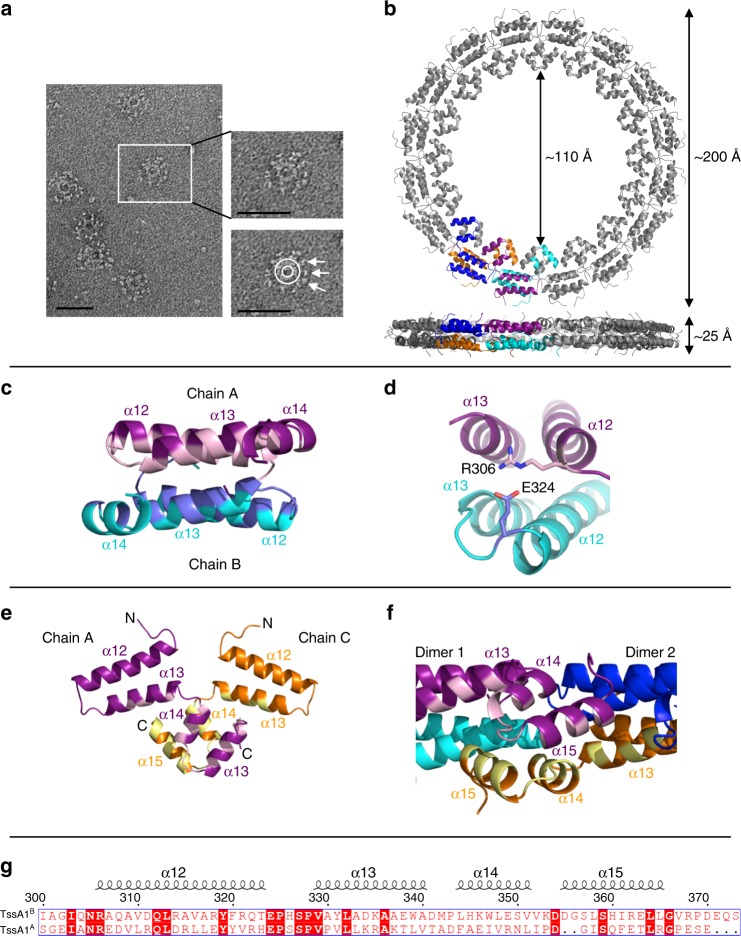
Structure of the Bc TssA1^B^ CTD ring. **a** Negative stain EM of TssA1^B^ particles. The magnified view indicates the location of the Nt1 domains (arrows) presented around the CTD oligomeric ring (circle). Scale bar corresponds to 50 nm. **b** Bc TssA1^B^ CTD monomers assemble to form a double-layered ring containing 32 subunits exhibiting D_16_ symmetry (PDB: 6HS6 [https://www.ebi.ac.uk/pdbe/entry/pdb/6hs6]). The views shown correspond to a top view down the 16-fold axis and a side view perpendicular to the 16-fold axis, with the dimensions of the ring shown. **c**, **d** Dimerisation of Bc TssA1^B^ CTD monomers. **c** Side view from inside the Bc TssA1^B^ CTD ring showing interaction of residues from helices α12, α13 and α14 within a dimer (chain A, purple; chain B, cyan). Interacting surfaces are highlighted (chain A, light pink; chain B, blue). **d** Formation of the salt bridge between R306 and E324 that caps both ends of the dimer interface. **e**, **f** Interactions between dimers to form the Bc TssA1^B^ CTD ring. **e** Expanded view of the two chains shown in purple and orange in (**b**), to show interactions leading to ring assembly. **f** Side view from inside the Bc TssA1^B^ CTD ring showing the interlocking interaction between helices α13, α14 and α15 from two dimers which are critical to ring formation around the 16-fold axis: dimer 1, purple/cyan; dimer 2, blue/orange. The interfacing regions of the purple and orange monomers are shown in light pink and yellow, respectively. **g** Structure-based sequence alignment of the Pa TssA1^A^ and Bc TssA1^B^ CTD domains. The secondary structure of TssA1^B^ CTD is highlighted above the sequence^75^

SEC-MALLS analysis of native Bc TssA1^B^ indicated an apparent molecular mass of 1.24 MDa (Supplementary Fig. 7c), suggesting that the TssA1^B^ oligomer contains ~30 subunits, and negative stain EM revealed rosette-like structures in which globular domains were irregularly distributed around a central ring (Fig. 5a). Two-hybrid analysis demonstrated that oligomerisation of TssA1^B^ required the CTD (Supplementary Fig. 7b), suggesting that the inner ring is composed of polymerised CTDs. This was confirmed by negative stain EM of the isolated CTD which was observed to form rings without peripherally located globular domains (Supplementary Fig. 7e).

The structure of a Bc TssA1^B^ CTD domain construct (I303-S373) was determined to 3.08 Å (Fig. 5b and Supplementary Table 3) and the asymmetric unit was observed to contain eight CTD monomers (chains A–H) (Supplementary Fig. 8a–c). Each chain folds into four α-helices (α12-α15) that lie in approximately the same plane, and form two antiparallel pairs (Fig. 5e). The eight monomers in the asymmetric unit further assemble into a higher order oligomer of 32 subunits, consistent with the SEC-MALLS analysis of TssA1^B^ and TssA1^B^ CTD oligomers (Supplementary Fig. 7c, d), with D_16_ symmetry using the crystallographic two-fold axes to make up a double-layered ring with 16 subunits in each layer (Fig. 5b and Supplementary Figure 8a). In the assembled Bc TssA1^B^ CTD ring, analysis by PISA^32^ revealed an extensive dimer interface (~950 Å^2^) between monomers in the different layers, which principally involves the interaction of residues located in helices α12–α13 with their two-fold related subunits forming a region of hydrophobic packing with hydrophilic interactions at the periphery (Fig. 5c, d). Further less extensive interactions (~570 Å^2^) were identified between dimers that facilitate formation of the ring, with each pair of helices α14-α15 in one dimer, together with a small number of residues from α13, interacting with their equivalents in adjacent 16-fold related dimers on either side (Fig. 5e, f). This type of interlocking series of interactions is repeated in a cyclic arrangement to form a double-layered ring (Supplementary Fig. 8d). Nested deletion of consecutive helical segments of TssA1^B^ CTD revealed that loss of α15 resulted in the failure of the protein to assemble into a high molecular weight ring, and structure determination of the construct H12–H14 (helices α12–α14) and of that consisting of α12 and α13 alone (residues 303–347), revealed them both to be dimers (Supplementary Figure 8e–h and Supplementary Tables 4 and 5).

In the crystal structure of the TssA1^B^ CTD oligomer, the double ring is ~25 Å thick with a lumenal diameter of ~110 Å and an outer diameter of ~200 Å (Fig. 5b), in agreement with the dimensions of Bc TssA1^B^ and purified CTD oligomers determined by negative stain EM analysis (Supplementary Fig. 7e, f). The C-termini of the TssA1^B^ CTD monomers are located at the inner face of the ring, while their N-termini are orientated on the outside, consistent with EM analysis, which places the Nt1 domain at the periphery of the ring in the intact complex (Fig. 5a). The irregular arrangement of the globular domains and the poor resolution of the region linking them to the inner ring are consistent with the presence of a flexible linker connecting the Nt1 domain to the CTD. Analysis of the CTD sequences of Bc TssA1^B^ and Pa TssA1^A^ identified 17 conserved residues, of which 11 are involved in interactions around the subunit interfaces, including the highly conserved EPxxP motif (Fig. 5g and Supplementary Fig. 2a). The Bc TssA1^B^ CTD structure shows that this motif defines a loop that connects α12 and α13, and includes a salt bridge between E324 from this motif and a conserved arginine, R306, from a symmetry-related subunit (Fig. 5d). The conservation of these residues suggests that the quaternary structures of TssA1^A^ and TssA1^B^ are related. Moreover, although it has been previously proposed that the C-terminal region of Pa TssA1^A^ is structurally similar to the C-terminal part of the gp6 subunit of the T4 phage baseplate on the basis of low level sequence similarity^28^, the similarity in sequence between TssA1^A^ and TssA1^B^ and the difference in structure of the TssA1^B^ CTD to gp6 indicates that this is not the case.

### TssA2^A^ Nt2 is structurally similar to the TssA1^B^ Nt1 domain

The structure of an Ah TssA2^A^ Nt2 domain construct (D231-L374) was solved to 1.76 Å with four subunits (chains A–D) in the asymmetric unit (Supplementary Table 6) arranged as two independent dimers, consistent with previous SEC analysis^33^, that showed that Ah TssA2^A^ Nt2 exists as dimers in solution. Each monomer folds into a cluster of seven α-helices (α1–α7), the arrangement of which is very similar to that of the EAEC TssA2^B^ Nt2 domain (RMSD = 2.0 Å) as determined by structural alignment^27,34^. Subsequent comparison using the Dali structural alignment server^35^ identified a match between the Sd1 domain of Bc TssA1^B^ Nt1 and the Nt2 domains of both Ah TssA2^A^ and EAEC TssA2^B^ (RMSD = 2.7 and 2.6 Å_,_ respectively) despite there being essentially no amino acid sequence similarity between them. Specifically, the related regions include helices α1-α5 of Bc TssA1^B^ Sd1 (i.e. most of the ImpA_N region) and α2–α6 of Ah TssA2^A^ Nt2 (Fig. 4d). This similarity suggests that an ancient gene duplication event may have played a role in the evolution of the Nt1 and Nt2 domains of TssA2 subunits.

### TssA2^A^ CTD assembles into an oligomer with D_5_ symmetry

**Fig. 6:**
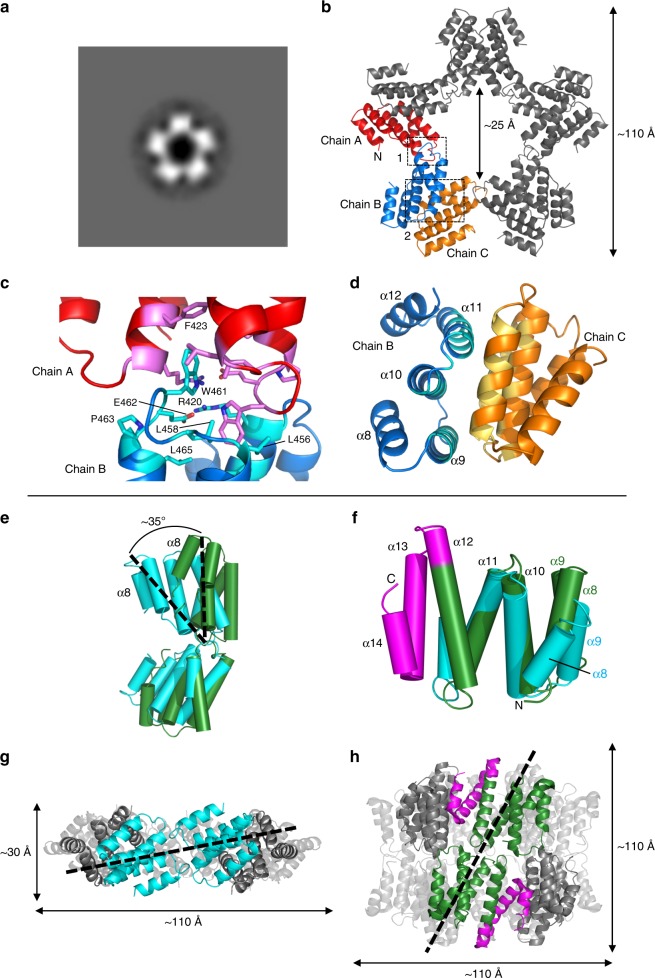
Structure of the Ah TssA2^A^ CTD ring. **a** Negative stain EM particle averaging of Ah TssA2^A^ CTD indicating five-fold symmetry. **b** Structure of 10 Ah TssA2^A^ CTD monomers assembled to form a decameric oligomer exhibiting D_5_ symmetry (PDB: 6G7C https://www.ebi.ac.uk/pdbe/entry/pdb/6g7c). Boxed region 1 (Interface 1), two-fold axis formed by dimerisation via the WEP motif (red and blue chains). Boxed region 2 (Interface 2), two-fold axis generated through packing of helices α9, α10 and α11 (blue and orange chains). The N-terminus of chain A is indicated and the dimensions of the ring are shown. **c** Interface 1 with key interacting residues indicated (chain A, pink: chain B, light blue). **d** Side view of interface 2 with interacting surfaces highlighted in light blue and sand. **e** Superposition of WEP-mediated dimers from Ah TssA2^A^ (cyan) and EAEC TssA2^B^ (PDB: 4YO5, green) about the WEP interface. The monomers exhibit a different relative orientation of ~35° about the WEP interface. View corresponds to chains A and B shown in (**b**). **f** Structural similarity of the CTD monomers of Ah TssA2^A^ (cyan) and EAEC TssA2^B^ (PDB: 4YO5, green). Helices α13 and α14 are unique to EAEC TssA2^B^ and form the C-terminal extension (magenta), which is important for the different quaternary structures assembled by TssA2^A^ and TssA2^B^. **g**, **h** Side by side views of the Ah TssA2^A^ (**g**) and EAEC TssA2^B^ (**h**) CTD oligomers with their respective five-fold and six-fold axes vertical. Assembly of Ah TssA2^A^ WEP-mediated dimers (cyan) around the five-fold axis involves a rotation relative to EAEC TssA2^B^ (green) of ~50° around the dimer axis as illustrated by the dotted black line. The neighbouring subunits with which each monomer within a WEP-mediated dimer interacts are shown in grey

Two-hybrid and SEC MALLS analysis indicated that Ah TssA2^A^ CTD self-associates and dictates the overall stoichiometry of the TssA2^A^ complex (Supplementary Fig. 9a, b, c, d). This is also a feature of EAEC TssA2^B^ ^27^. However, unlike EAEC TssA2^B^, the molecular weight estimations of Ah TssA2^A^, Ah TssA2^A^ CTD and an Ah TssA2^A^ Nt2-CTD fusion construct corresponded to a subunit stoichiometry of 10 rather than 12. Consistent with this, negative stain EM showed that Ah TssA2^A^ CTD and Ah TssA2^A^ Nt2-CTD assembled into complexes that resembled a five-pointed star (Fig. 6a and Supplementary Fig. 9e, f). The structure of crystals of an Ah His_6_.TssA2^A^ Nt2-CTD construct (R232-K478) was successfully determined to 3.13 Å through molecular replacement using the coordinates of the Ah TssA2^A^ Nt2 dimer as a search model (Supplementary Table 7). This identified the position of five dimers of the TssA2^A^ Nt2 domain (R232 to L374, helices α1–α7) in the asymmetric unit, which did not appear to be arranged with any obvious symmetry with respect to each other. Following refinement, these dimers could be seen to be essentially identical in structure to that of the dimer formed by the isolated TssA2^A^ Nt2 domain (RMSD = 0.4 Å). The remaining C-terminal residues of each of the 10 subunits (chains A-J) were organised into a separate domain (the CTD) consisting of five α-helices (G388-L472, α8–α12). The 10 CTDs were, in turn, assembled into a flat ring resembling a star with D_5_ symmetry (Fig. 6b), consistent with the negative stain EM and the SEC-MALLS analysis.

In the assembled TssA2^A^ CTD decamer two distinct two-fold interfaces can be identified (Fig. 6b). In the first, a two-fold related interface (shown by PISA^32^ to be 530 Å^2^) is formed primarily through interactions between residues from the extended loop linking α11 and α12 (Fig. 6b, c) and the N-terminal end of α10 and their symmetry-related partners. This loop includes a WEP motif (W461, E462 and P463) that is conserved across both TssA2 sub-clades, where W461 is partially buried within a hydrophobic pocket formed by the conserved residues L456, L458 and L465, and an interaction with F423, a non-conserved residue, in the two-fold related monomer. The entrance to this pocket is capped by a salt bridge between E462 of the motif and R420, two residues that are again conserved (Fig. 6c and Supplementary Fig. 2b). In the second, more extensive interface (950 Å^2^), the WEP-mediated dimers assemble around the five-fold axis through interactions between the surface of helices α9, α10 and α11 (Fig. 6b, d) and their two-fold related symmetry mates to form a mixed interface of hydrophobic/hydrophilic contacts. The TssA2^A^ CTD (S381-K478) ring has a thickness of ~30 Å, an outer diameter of ~110 Å, and a lumenal diameter of 25 Å (Fig. 6b, g). As in the structure of the Bc TssA1^B^ CTD ring, the N-terminus of each TssA2^A^ CTD monomer is orientated on the outside of the CTD oligomer resulting in the presentation of the Nt2-CTD interdomain linker at the periphery of the molecule (Fig. 6b).

Despite the differences in symmetry between TssA2 CTD oligomers assembled by members of sub-clades 2^A^ and 2^B^, the fold of the Ah TssA2^A^ CTD monomer is closely related to that of its counterpart in EAEC TssA2^B^ (PDB = 4YO5, RMSD = 1.6 Å). Moreover, close inspection shows that the conserved WEP motif is maintained at the dimer interface in members of both TssA2 sub-clades, albeit that the two monomers of Ah TssA2^A^ are arranged in a slightly different orientation with respect to each other, corresponding to a rotation of ~35° compared to their TssA2^B^ counterparts (Fig. 6e).

The way in which the dimers assemble around their respective five-fold and six-fold symmetry axes in the Ah and EAEC TssA2 CTDs are strikingly different, in contrast to the similarity seen around their two-fold axes. In Ah TssA2^A^, two monomers, related by a WEP-mediated two-fold axis, pack adjacent to one another, parallel to the five-fold axis, resulting in the formation of an oligomer which resembles two interpenetrating rings (Fig. 6g). The face of Ah TssA2^A^, used in subunit assembly around the five-fold axis (residues from α9, α10, and α11), interacts with a symmetry related equivalent (Fig. 6g). In comparison, EAEC TssA2^B^ CTD contains a 41 residue C-terminal extension which folds into two additional helices (α13 and α14) (Fig. 6f). These dominate the interactions around the six-fold axis, packing against the face of helices α9, α10 and α11 of an adjacent subunit (Fig. 6h). Consequently, this causes an incline of the WEP-mediated dimer by ~50°. Therefore, in EAEC TssA2^B^, two monomers, related by a WEP-mediated two-fold axis, stack on top of one another, parallel to the six-fold axis, with the dimers then assembling around the latter to form a double-layered ring (Fig. 6h). Interestingly, despite the difference in symmetry we note that the lumenal diameter and outer diameter of the TssA2^A^ and TssA2^B^ CTD oligomers are similar (Fig. 6g, h), thereby placing the Nt2-CTD interdomain linker at the same distance from the centre of the complex.

### A flexible linker in TssA2^A^ Nt2-CTD mediates domain mobility

**Fig. 7:**
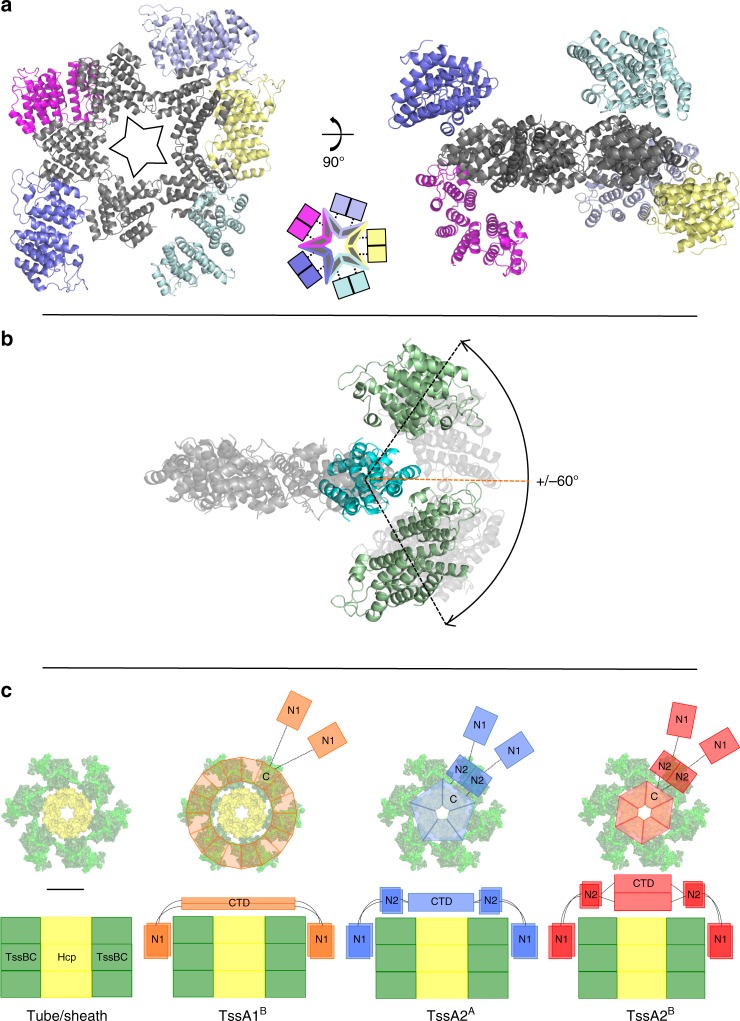
Schematic representation of TssA ring dimensions and domain flexibility. **a** Arrangement of the Ah TssA2^A^ Nt2 domains around the CTD oligomer as observed in the asymmetric unit. The Nt2 domains do not follow strict five-fold symmetry. The decameric CTD ring is shown in dark grey perpendicular to the five-fold (left) and two-fold (right) axes with each peripheral Nt2 dimer shown in a different colour. The diagram between the two structures shows the relationship between dimer-forming Nt2 domains (represented by coloured squares) and their corresponding C-terminal domains in the TssA2^A^ Nt2-CTD decamer. **b** Displacement of the Ah TssA2^A^ Nt2 dimers perpendicular to the five-fold axis of the CTD oligomer. The CTD ring is shown in light grey with one WEP-mediated CTD dimer indicated in cyan to which is tethered an Nt2 dimer. The Nt2 dimer (light green) is shown at its maximum displacement of ~60° either side of the plane of the CTD ring (orange line). Other observed positions of Nt2 are indicated by translucent shading. **c** Top and side view representations showing the domain architecture of TssA subunits from clades 1^B^, 2^A^ and 2^B^ modelled on the baseplate-distal end of the T6SS tube/sheath assembly during polymerisation. The *V. cholerae* TssBC extended sheath (green) surrounding a Hcp hexamer (yellow) (PDB: 5MXN) is shown on the left. Onto this, is modelled the TssA1^B^ CTD oligomer (orange) with 16-fold symmetry, showing one stacked CTD dimer attached to a pair of Nt1 domains via flexible linkers, the TssA2^A^ CTD oligomer (blue) with five-fold symmetry, showing a WEP-mediated CTD dimer connected to the two monomers of an Nt2 dimer via flexible linkers, each of which are in turn connected to an Nt1 domain, and the TssA2^B^ CTD oligomer (red) displaying six-fold symmetry, showing a CTD dimer connected to the two monomers of an Nt2 dimer via flexible linkers, each of which are in turn connected to an Nt1 domain. Labels are: C, CTD dimers (top view); N1, Nt1; N2, Nt2; CTD, CTD oligomer (side view). Scale bar corresponds to 65 Å

Despite the electron density for the Nt2 and CTD domains being clear, density was observed for only part of the Nt2-CTD interdomain linker, and, depending on the chain, between 6 and 13 residues (Q375-A387) could not be identified within this region. However, restrictions imposed by the maximum length of the absent linker residues and the relative orientations of the Nt2 and CTD domains allowed the unambiguous assignment of the Nt2 domains to their corresponding CTD (i.e. in the same polypeptide chain). This revealed that in the decameric Ah TssA2^A^ Nt2-CTD complex, the Nt2 domains are not related by the five-fold symmetry exhibited by their corresponding CTDs (Fig. 7a). Instead, dimers of Nt2 make no contacts with their own CTDs but rather contact either TssA2^A^ Nt2 or CTD domains of other decamers in the crystal lattice. Moreover, as a result of the flexible linker, the Nt2 dimers have quite different orientations with respect to the five-fold axis, with angular rotations of up to ~60° about the vertical axis (Fig. 7b), and ~45° and ~60° about the horizontal and torsional axes, respectively. Although, the observed Nt2 domain orientations described above are believed to be an artefact of crystal packing, the range of motion seen is believed to reflect the inherent flexibility between CTD and Nt2 domains. This is consistent with negative stain EM analysis that revealed a lower calculated correlation coefficient for strict five-fold symmetry when comparing an Nt2-CTD construct to a CTD construct (Supplementary Fig. 9e–h).

Sequence alignments suggest that the Nt2-CTD interdomain linker is longer in TssA2^B^ subunits than in TssA2^A^ subunits (Supplementary Fig. 2b). For example, the linker in Ah TssA2^A^ is composed of ~21 residues whereas it contains ~37 residues in EAEC TssA2^B^. The most likely explanation for the presence of a longer linker in TssA2^B^ is to overcome a restricted range of motion of Nt2 dimers that would be imposed by a shorter linker due to the possible physical interference between the Nt2 dimer and the ‘taller’ double-layered ring arrangement formed by EAEC TssA2^B^ CTD compared to the ‘squat’ interpenetrating ring assembled by Ah TssA2^A^ CTD (Fig. 6g, h).

The quite different orientation of the CTD WEP-mediated dimers in TssA2^A^ and TssA2^B^ oligomers has one further consequence, which is that the orientation of the interdomain linker between Nt2 and CTD is necessarily rotated by ~50° (Fig. 6g, h). We assume that the flexibility of the linker allows this structural difference to be maintained without affecting the biological role of the protein. The finding that in the Ah TssA2^A^ Nt2-CTD structure the Nt2 domains consistently occur as dimers further suggests that the Nt2 domains remain associated as part of the biological role of TssA, in contrast to the proposal by Zoued and co-workers^27^, who interpreted low resolution negative stain EM data to suggest that they separate to form monomers^27^. A rigorous test will require a high-resolution EM reconstruction from TssA particles embedded in vitreous ice, although this could be challenging given the apparent flexibility of domain linkers.

## Discussion

The structural studies presented here show that TssA1^B^, TssA2^A^ and TssA2^B^ possess structurally diverse CTDs that lead to distinct overall architectures for their respective complexes. Nevertheless, all these subunits contain a conserved N-terminal ImpA_N region that is peripherally located in the assembled complexes. The question that arises from this is whether these TssAs play the same role in the function of the T6SS? The presence of the ImpA_N region in the Nt1 domain of subunits from all TssA clades strongly suggests that they have a common function that is of critical importance. This is further supported by the studies described here which reveal that TssA1^B^ interacts with the baseplate components TssE, TssF and TssK, the inner tube subunit, Hcp, the tip protein, VgrG, the sheath subunit TssC and cytoplasmic components of the cell envelope-anchoring complex. This is strikingly similar to the TssA2^B^ subunit that was shown to interact with the same components except for TssF^27^. Indeed the TssA1^B^ and TssA2^B^ CTD oligomers were both shown to interact with Hcp and VgrG despite their quite different structures. This would argue for a similarity of function despite the divergent architecture of the two proteins. Given the sequence similarity and predicted structural relationship between TssA1^A^ and TssA1^B^, this suggests that all three clades of TssA perform related roles within the T6SS. If the role of these quite different TssA subunits is similar, then how is this achieved given the structural variations that are observed?

To date, two distinct models for the role of TssA have been proposed. In the first, baseplate-associated model, it was proposed that TssA is a component of the T6SS baseplate required for priming the inner tube and sheath for assembly, but not migrating during polymerisation^28^. This model was based partly on predicted similarities in structure for the CTDs of TssA1^A^ and the phage T4 baseplate protein gp6, to which it was believed that this TssA subunit was related. The structure we have obtained for TssA1^B^, which is closely related to TssA1^A^, reveals that it has a quite different structure to gp6. This suggests that TssA1^A^ is very unlikely to be a gp6 orthologue. Due to the absence of structural similarity to baseplate proteins and the conservation of the Nt1 domain among all TssA subunits, it is therefore likely that TssA1 subunits fulfil a similar role to TssA2 subunits.

In the alternative model proposed for a TssA subunit that corresponds to TssA2^B^, TssA acts as a coordinator of inner tube and sheath assembly, in which the CTD oligomer of TssA2^B^ would lie close to the centre of the tail assembly, above the position occupied by Hcp where it would coordinate polymerisation of the inner tube and sheath^27,29,36,37^. In one version of this proposal (the cap model) eversion of CTD monomers, from a closed form of the CTD oligomer to an open form, is then proposed to permit the passage of Hcp through the lumen of the open ring. This would allow sequential stacking of Hcp hexamers, with the N-terminal domains of TssA2^B^ interacting with the polymerising sheath. However, as yet, an open form of TssA2^B^ has not been observed and the proposed reorientation of monomers in the CTD oligomer remains speculative.

The studies described here show that the fold of TssA2^A^ retains clear structural similarity to TssA2^B^ yet generates a decamer possessing five-fold symmetry in contrast to the six-fold symmetry of the TssA2^B^ dodecamer^27^. Therefore, while it could be argued that it might lie in the same position in the complex as TssA2^B^, the difference in symmetry implies there must be changes to the complementary surfaces of surrounding TssB/-C/-D subunits to allow pseudo equivalent interactions. This situation becomes even more extreme given the quite different organization of the TssA1^B^ ring oligomer with its sixteen-fold symmetry. One possibility is that the structure of TssA1^B^ we have determined represents an open form of the TssA CTD oligomer, leaving a closed form of the TssA1^B^ CTD oligomer, which might pack on top of a Hcp hexamer, still to be identified. Alternatively, if some features of the cap model are correct, and TssA1^A^/TssA1^B^ and TssA2^A^ also serve to coordinate tube and sheath assembly, then, given their quite different symmetries and architectures, rationalising how they function in an equivalent way to that proposed for TssA2^B^ is problematic. One way for this dilemma to be resolved is if the different structures of these distinct TssAs represent different ways in which a common function can be maintained.

Comparing the TssAs of clades 1 and 2, these structures indicate that the Nt1 domain is linked either to a large diameter CTD ring (200 Å) of high symmetry, via a single flexible segment, as seen in sub-clade TssA1^B^, or to a smaller (100–110 Å) diameter CTD of lower symmetry, through a middle domain (Nt2) flanked by two flexible linkers, as seen in sub-clades 2^A^ and 2^B^. Despite these two alternative architectures, the radial position of the Nt1 domain from the centre of the complex is similar in both TssA1 and TssA2 clades (Fig. 7c). This suggests that a role of the Nt2 domain is to act as a spacer connecting the Nt1 domain of TssA2^A^ and TssA2^B^ to their corresponding CTDs.

In light of the above, and given the conservation of the Nt1 domain, we suggest that the common feature shared by all these TssAs is that the Nt1 domain is positioned around the periphery of the ring-forming CTD oligomers, furthest away from the symmetry axis but at an equivalent distance. Given the apparent symmetry mismatch between the tube/sheath assembly and the TssA CTD oligomers of some TssA species, it is important to consider how equivalent interactions between these components can be maintained. The simplest explanation is to consider a model whereby the interactions between TssA subunits and the tube/sheath assembly are solely the property of the conserved Nt1 domains, which have been suggested to participate in assembly of the latter^27^. This would mean that the role of the CTD or Nt2-CTD oligomers of clades 1 or 2, respectively, are to provide a platform for the presentation of the Nt1 domains above the growing tube/sheath complex at a similar distance from the centre of each TssA complex (Fig. 7c). In such a model it would not be necessary for the CTDs to interact directly with the tube/sheath assembly except perhaps in a transient manner as indicated by interaction studies. This agrees with surface plasmon resonance data for EAEC TssA2^B^ where the interaction of the Nt1-Nt2 region with the TssBC sheath is significantly greater than that of the corresponding CTD with Hcp^27^.

This proposal in which one domain or component of a protein subunit (CTD/Nt2-CTD) associates into a platform that facilitates the role of a second domain (Nt1) in subunit incorporation by a polymerising protein assembly, is a theme that occurs in the mechanism of flagellum assembly by the HAP2 cap protein^27,29,38,39^. An important aspect of the flagellin-capping model is the observation of domain mobility, a feature clearly shown here for both the relative motion of Nt1 with respect to the CTD in TssA1^B^ and between the CTD and Nt2 in TssA2^A^ and TssA2^B^, and the predicted flexibility of the linker between Nt2 and Nt1 in TssA2^A^/TssA2^B^. This would provide the necessary movement of the Nt1 domains for dynamic interactions with the TssBC subunits during sheath biogenesis. This might involve the displacement of Nt1 domains and their linkers, consequently allowing passage of Hcp, in addition to further TssB/C subunits to be incorporated into the growing sheath. This model does not necessarily require eversion of the CTD rings. However, we do not rule out the possibility that TssA2^A^/TssA2^B^ CTD oligomers can re-orientate to allow access of Hcp hexamers to the growing tube. In this regard, we note that the TssA1^B^ CTD ring oligomer can adequately accommodate Hcp hexamers within its lumen.

A question arising from the dihedral symmetry shown by all TssA structures that have been determined to date, is how this leads to a uni-directional assembly of the tube/sheath complex. While any initial interaction between TssA and other subunits of the complex can use either two-fold related face of the TssA oligomer, once contact has been made with T6SS components, the symmetry is broken. This would occur when TssA interacts with cytoplasmic components of the TssJLM membrane complex that occurs during the initial steps in T6SS assembly^27^, as one surface of the TssA ring faces the membrane while the other faces the cytosol. According to the current model, this asymmetry is reinforced once the baseplate is recruited to the membrane complex, whereupon one face of the TssA oligomer is located adjacent to the cytoplasmic face of the baseplate^27^. Here TssA is ready to coordinate inner tube and sheath assembly once the first Hcp ring is recruited to the base of the VgrG trimer located at the hub of the baseplate^40^. Although the precise mode of action of TssA subunits remains unknown, they are likely to lock the inner tube/sheath structure in the high energy form prior to firing^29^. Thus, TssA may possess chaperone-like activity.

A final question is to consider the mismatch in symmetry between the assemblies formed by members of different TssA clades, on the one hand, and other components of the T6SS, on the other, and how this impacts on the mechanism by which TssA executes its role. For example, the six-fold symmetry of the TssA2^B^ oligomer is consistent with the symmetry of the tube-sheath structure but not with the five-fold symmetry proposed for the TssJLM membrane complex^10,27^. In contrast, the symmetry of the TssA2^A^ oligomer matches that of the membrane complex but not the contractile machinery. In this regard it is interesting to note that, as seen with the different symmetries of TssA2^A^/TssA2^B^, both five-fold and six-fold symmetrical variants of the HAP2 capping protein can interact with the common 11-fold symmetrical flagellin filament^41,42^ suggesting that the apparent symmetry mismatch between oligomers formed by some TssA species and the tube/sheath assembly is not necessarily problematic. Based on these considerations we suggest that a flagellin-like capping model is a plausible mechanism for the role of all TssA subunits. Ultimately, these proposals need to be confirmed by the structural analysis of other complexes of secretion systems from each of the different TssA clades. Once this has been achieved, the manner in which the difference in structure and symmetries among TssA subunits resolves to provide equivalent function will be revealed.

## Methods

### Bacterial strains and growth conditions

Bacterial strain details are given in Supplementary Table 8. *E. coli* strains JM83 and DH5α were used for routine cloning steps. *B. cenocepacia* strain H111 and *A. hydrophila* ATCC 7966 were the source of the TssA1^B^ and TssA2^A^ subunit genes, respectively. LB was routinely used for growing bacteria except where indicated^43^. Lennox medium was used with glucose included at 0.2%^44^. D-BHI broth was made by dissolving 9.25 g brain-heart infusion (BBL) in 25 ml water and dialysed (12,000–14,000 MWCO) against 500 ml water overnight^45^. Antibiotics were used at the following concentrations for *E. coli*: ampicillin, 100 μg/ml; kanamycin, 50 μg/ml; chloramphenicol, 25 μg/ml; trimethoprim, 25 μg/ml (selection for trimethoprim resistance was applied in/on iso-sensitest (IST) broth/agar (Oxoid)). Bacto MacConkey agar base used for two-hybrid assays was obtained from Becton, Dickinson and Co.

### Plasmids and primers

All oligonucleotides and plasmids used in this study are listed in Supplementary Tables 9,10. Construction of plasmids is also described in Supplementary Table 10. Plasmids were transferred to *E. coli* by transformation^46^ and to *B. cenocepacia* by conjugation^47,48^.

### Construction of a *B. cenocepacia tssA1*^*B*^ mutant

To construct the *B. cenocepacia* H111 *tssA*::Tp mutant, a segment of the *tssA1*^*B*^ ORF was amplified with primers iotAfor3 and iotArev and cloned between the *Hin*dIII and *Bam*HI sites of pBBR1MCS (to give pBBR1MCS-‘tssA1^B^) whereupon it was disrupted by insertion of the *dfrB2* (Tp^R^) cassette from p34E-Tp (generating pBBR1MCS-‘tssA1^B^::Tp). The *tssA1*^*B*^::Tp fragment was then transferred to the suicide vector pSHAFT2 and the resultant plasmid (pSHAFT2-‘tssA1^B^::Tp) conjugally mobilised into H111 using *E. coli* S17–1(λpir). Ex-conjugants were selected on M9-glucose agar containing trimethoprim (25 μg/ml). Mutants were identified by PCR screening chloramphenicol-sensitive ex-conjugants using primers iotAfor and iotArev2 that anneal to sequences located outside the genomic region contained on the suicide vector. Construction of the *tssM* deletion mutant was performed by conjugally mobilising the allelic replacement plasmid, pSNUFF-ΔtssM, containing a *tssM* deletion allele (codons 506–1216 removed) into strain H111 using *E. coli* SM10(λpir). Ex-conjugants containing the integrated allelic replacement vector were selected on M9-glucose agar containing trimethoprim (25 μg/ml) and were then plated on M9-glucose agar containing *p*-chlorophenylalanine to identify recombinants in which a second crossover had resulted in excision of the integrated plasmid^49^. Mutants were identified by diagnostic PCR using primers tssM-OPfor and tssM-OPrev2.

### Hcp secretion assay

D-BHI broth was inoculated with overnight cultures of *B. cenocepacia* strains to give a starting OD_600_ of 0.03, and grown to an OD_600_ of 1.0 at 37 °C. Cultures were centrifuged to collect the cells and the supernatant was filter-sterilised. To precipitate secreted proteins, sodium deoxycholate was added to the cleared supernatant to a final concentration of 0.2 mg/ml followed by incubation on ice for 30 min. Trichloroacetic acid was then added to a final concentration of 10% (v/v), and the suspension was incubated overnight at −20 °C. Precipitated proteins were recovered by centrifugation for 15 min at 14,000 × *g* at 4 °C and washed with ice cold acetone. Pellets were air-dried and resuspended in 0.001 of the original volume of SDS-PAGE sample buffer. Cell-associated proteins were isolated by resuspending the initial bacterial pellet in a volume of SDS-PAGE sample buffer that was 20-fold less than the starting culture volume and transferred to a microcentrifuge tube. All protein samples were boiled for 5 min, centrifuged for 10 min at 14,000 × *g* to remove cell debris and then fractionated by electrophoresis in a 15% discontinuous SDS-PA gel followed by electroblotting onto a PVDF membrane (Millipore). Membranes were blocked with 5% (w/v) non-fat skimmed milk in TBS containing Tween 20 (0.05% (v/v)) solution for 1 h. Hcp was detected by probing with a custom polyclonal rat antibody raised to purified His_6_.Hcp (1:1000) and goat anti-rat HRP secondary antibody (1:5000). Antibody to the cytoplasmically located β subunit of *E. coli* RNA polymerase (1:2000) that cross-reacted to the *Burkholderia* spp. subunit was also used as a lysis control, with a rabbit anti-mouse HRP secondary antibody (1:5000). Detection of chemiluminescence was performed using an EZ-ECL detection kit and a XRS+ imaging system (BioRad). Uncropped blots are shown in Supplementary Fig. 5.

### Two-hybrid analysis

The bacterial adenylate cyclase two-hybrid (BACTH) system was employed^50^. *B. cenocepacia* T6SS subunit genes (*tssA*-*tssC*, *hcp*, *tssE*-*tssG*, *vgrG*, *tssJ*-*tssM*) or domain coding sequences were fused to the N- and C-terminal coding sequences of the *B. pertussis* CyaA T25 or T18 fragments present in plasmids pKT25, pKNT25, pUT18 and pUT18C. To avoid membrane or periplasmic targeting of CyaA fusion proteins, signal sequences and TMDs were omitted from the TssJ, TssL and TssM hybrid proteins. As a consequence, the cytoplasmic N-terminal region of TssM (TssM_N_) and the periplasmic C-terminal region (TssM_C_) were fused to the CyaA domains separately. Hybrid proteins were generated with full-length VgrG and also with the conserved core region corresponding to the phage T4 gp27 and gp5 proteins (VgrG_C_). DNA encoding full-length *A. hydrophila* TssA2^A^ and the TssA2^A^ Nt1 domain were also cloned into all four vectors while DNA encoding the Nt2 and CTDs were cloned into pKT25 and pUT18C only. Further details on plasmid constructions are given in Supplementary Table 10. The *E. coli cya*^−^ strain BTH101 was transformed with the two compatible plasmids expressing fusion proteins and plated on MacConkey indicator agar containing 1% (w/v) maltose, ampicillin (100 μg/ml) and kanamycin (50 μg/ml). Colony phenotypes were scored after 3 and 5 nights incubation at 30 °C (Mal^+^ (Cya^+^) colonies are purple and Mal^−^ (Cya^−^) colonies are white). The appearance of purple colonies after 3 days incubation indicated a strong Cya^+^ phenotype. The positive control combination (Zip control), where each plasmid encoded the leucine zipper segment of the yeast transcriptional activator protein, GCN4, fused to T25 and T18^50^ was always included as were all negative control combinations (two compatible empty vectors and one empty vector in combination with a plasmid specifying a hybrid protein).

The efficiency of CyaA complementation was quantitated by measurement of the β-galactosidase activity in broth cultures. Cells were grown in LB broth containing antibiotics and 0.5 mM IPTG at 30 °C with aeration for 14–16 h. The cultures were then chilled on ice before being diluted in LB medium to OD_600_ 0.4–0.6. 50 μl of each diluted culture was added to test tubes containing 950 μl of Z buffer (0.06 M Na_2_HPO_4_, 0.04 M NaH_2_PO_4_, 0.01 M KCl, 0.001 M MgSO_4_ and 0.27% (v/v) β-mercaptoethanol), chloroform (30 μl) and 0.1% SDS (30 μl). The mixtures were vortexed for 10 s and equilibrated at 30 °C for 15 min whereupon 200 μl of ONPG (4 mg/ml in Z buffer) was added to initiate the reaction. Reactions were stopped by addition of 500 μl 1 M Na_2_CO_3_ and the absorbance at 420 and 550 nm was measured. The β-galactosidase activity (in Miller units) was calculated using the equation 1000 (OD_420_–1.75 × OD_550_/*t* x *v* × OD_600_) where *t* corresponds to the duration of the reaction in minutes and *v* corresponds to the volume of culture used in the assay^43^. The background activity measured in the negative controls (a pair of empty vectors or one empty vector in combination with a plasmid encoding a hybrid protein) was 75–90 Miller units (Mu). Each assay reaction was performed in duplicate on three biological replicates (*n* = 3), and the mean, along with standard deviation, was calculated.

### Co-immunoprecipitation assay

Co-IP analysis was performed on cell lysates following co-expression of N-terminal FLAG-tagged TssA1^B^ as bait and an epitope-tagged T6SS subunit (or domain) as prey in *E. coli*. FLAG.TssA1^B^ was expressed from the second T7 RNAP-dependent promoter contained on pACYCDuet-1 while the prey protein was expressed from the upstream promoter on the same plasmid (except where indicated below). Prey proteins were generally tagged at their N-terminus with the vesicular stomatitis virus glycoprotein (VSV-g) epitope tag (TssC was C-terminally tagged). TssF was tagged at the N- or C-terminus with the human influenza haemagglutinin (HA) epitope tag. In two cases, a separate plasmid was used to express the prey protein: N-terminal HA-tagged TssF was expressed from the downstream T7 promoter of pETDuetΔO whereas MBP-TssE was expressed from the *tac* promoter present on pMAL-c5X. Further details on plasmid constructions are given Supplementary Table 10.

Expression of all proteins was induced by addition of 0.5–1 mM IPTG (0.1 mM for the TssC co-IP) to *E. coli* BL21(DE3) cells grown to OD_600_ 0.5–0.7 in 50 ml BHI broth at 37 °C (for the Hcp, TssK and TssL co-IPs), 30 °C (for the TssE, TssF and VgrG_C_ co-IPs) or 22 °C (for the TssC and TssM_N_ co-IPs), according to the solubility of the prey protein, and incubation was continued at the same temperature for 2–3 h. Bacterial cells were harvested and stored frozen at −20 °C overnight. Defrosted *E. coli* cell pellets containing co-expressed proteins were resuspended in TBS (50 mM Tris-Cl (pH 7.4), 150 mM NaCl (or 500 mM NaCl for VgrG_C_ and TssM_N_)) with EDTA-free protease inhibitor tablets (Roche) at a ratio of 5 ml TBS to 1 g cell pellet. Following sonication, the soluble fraction was cleared by centrifugation at 20,000 *g* for 15 min at 4 °C, and MgCl_2_ was added to a final concentration of 2.5 mM. After gently mixing by inversion, the mixture was centrifuged at 13,000 *g* for 10 min. The supernatant was then incubated with Tween 20 (0.2%) for one hour with gentle mixing on a rotating wheel at room temperature, and followed by centrifugation at 15,000 *g* for 10 min. The supernatant was used for co-immunoprecipitation with the anti-FLAG affinity gel. Fifty microlitres of the anti-FLAG M2 affinity resin suspension equilibrated in TBS was added to 200–1000 μl cell lysate (depending on the abundance of FLAG-tagged TssA1^B^ in the lysate) and the final volume was brought to 1 ml by adding TBS. After 2–3 h gentle mixing at 4 °C to allow for precipitation, the mixture was centrifuged at 7000 × *g* for 30 s, and the supernatant was removed (unbound material). The resin was then washed three times with 1 ml TBS with gentle mixing for 5–15 min at 4 °C between each wash followed by centrifugation at 7000 × *g* for 30 s. After discarding the supernatant of the final wash, the immunoprecipitated material was dissociated from the resin by boiling with 25 μl of SDS-PAGE sample buffer with or without 5% β-mercaptoethanol for 3 min. Finally, the mixture was centrifuged at 7000 × *g* for 30 s to clear the suspension. The supernatant (immunoprecipitated material) was subjected to SDS-PAGE alongside a pre-stained protein ladder, blotted onto PVDF membrane and probed with tag-specific antibody (anti-VSVg, anti-HA or anti-FLAG, each at 1:5000–1:10,000 dilution) and detected with HRP-conjugated secondary antibody (1:5000–1:6666 dilution). MBP fusion proteins were detected with HRP-conjugated anti-MBP (1:5000 dilution). The sizes of the tagged *B. cenocepacia* T6SS subunits in kDa (excluding the N-terminal formyl-methionine) are: TssA1^B^, 42.6; TssC, 56.1; Hcp, 19.8; TssE, 61.6; TssF, 69.8 (both versions); VgrG_C_, 60.7; TssK, 51.3; TssL, 24.4; TssM_N_, 44.5; MBP, 45.5 kDa. Uncropped blots are shown in Supplementary Fig. 4.

### Antibodies

Polyclonal antibody to *B. cenocepacia* Hcp was raised in rats at the University of Sheffield Biological Services unit using N-terminal His-tagged Hcp which was purified following its overproduction in *E. coli* as described below. Rabbit anti-FLAG mAb and anti-VSVg pAb were obtained from Sigma-Aldrich (cat. no. F7425 and V4888, respectively), whereas rabbit anti-HA mAb was purchased from Cell Signaling Technology (cat. no. C29F4). HRP-conjugated mouse anti-MBP polyclonal antibodies were obtained from New England Biolabs (cat. no. E8038S). The mouse mAb to the β subunit of *E. coli* RNA polymerase was obtained from Neoclone (cat. no. W0023). Proteins harbouring a polyhistidine tag were detected using the HisProbe-HRP conjugate (Thermo Fisher, cat. no. 15165) or mouse anti-KLH-conjugated His-tag peptide (BioLegend, 652501). HRP-linked goat-derived (secondary) antibodies that cross-react to rat and rabbit IgG were obtained from SouthernBiotech (cat. no. 3050–05) and Vector Laboratories Ltd (cat. no. PI-1000), respectively. HRP-conjugated rabbit anti-mouse IgG secondary antibody were purchased from Thermo Fisher (cat. no. 31450).

### Expression and purification of Hcp for antibody production

His_6_.Hcp was overproduced following a 4 h induction of the T7 promoter on pET14b-His_6_.TssD2 carried by *E. coli* BL21(DE3) cells growing in BHI at 37 °C. His_6_.Hcp, which lacks the first three native Hcp amino acids, was purified by sequential IMAC on nickel-sepharose (HisTrap HP, GE Healthcare Life Sciences) and SEC using a Superose-6 10/300 GL column equilibrated in PBS. Prior to the SEC step, the protein was concentrated using a 10,000 MWCO centrifugal concentrator.

### Expression and purification of TssA subunits and domains

Expression and purification of His_6_.TssA2^A^ (1–478), His_6_.TssA2^A^ Nt2 (223–387), His_6_.TssA2^A^ Nt2-CTD (223–478) and TssA2^A^ CTD (381–478) was carried out as previously described^33^. Both MBP.TssA1^B^ CTD (303–373) and His_6_.TssA1^B^ Nt1 (1–255) were overexpressed and purified, with TssA1^B^ CTD (303–373) being separated from the MBP solubility tag by Factor Xa cleavage^51^. MBP.TssA1^B^ CTD (294–373) was produced and purified in a similar manner as MBP.TssA1^B^ CTD (303–373). However, following Factor Xa cleavage of MBP.TssA1^B^ CTD (294–373), TssA1^B^ CTD oligomers were separated from the MBP solubility tag for EM analysis using a 100 kDa MWCO concentrator (Amicon) rather than SEC and the additional amylose column purification step was not included. Truncated TssA1^B^ CTD derivatives were overproduced with an N-terminal MBP-His_6_ tag in a similar manner to that described for MBP.TssA1^B^ CTD (303–373) but were purified by IMAC on a HisTrap HP column with imidazole gradient elution rather than by amylose affinity chromatography. For crystallisation, MBP.His_6_.TssA1^B^ CTD H12–H14 (amino acids 303–358) was purified by amylose affinity chromatography, and eluted with 10 mM maltose. His_6_.TssA1^B^ CTD H12–H14 was proteolytically removed from the MBP tag by treatment with Factor Xa, followed by purification on a nickel column with imidazole gradient elution and SEC on a Superdex 200 column prior to concentration and final buffer exchange^51^.

TssA2^A^ Nt1-Nt2.His_6_ (1–374), His_6_.TssA2^A^ CTD (381–478) and the TssA1^B^ variants (Native TssA1^B^ (1–373), His_6_.TssA1^B^ (1–373) and linkerHis_6_.TssA1^B^ (1–373)) were overproduced by inducing the T7 promoter present on the appropriate expression vector derivative in *E. coli* BL21(DE3) cells growing in BHI broth at 37 °C by addition of IPTG (final concentration 1 mM) to the culture at OD_600_ 0.5–0.7 and incubation was continued for a further 2–3 h (30 °C was used for inducing the linker His-tagged derivative). Harvested cells were resuspended in 50 mM Tris-Cl (pH 8.0), 100 mM NaCl (5 ml/g cell paste) and lysed by sonication. If the construct contained a His-tag, the lysate was cleared by centrifugation at 35,000 × *g* for 30 min. The clarified lysate was applied to nickel-sepharose (HisTrap HP, GE Healthcare Life Sciences) and eluted with a linear gradient of imidazole (10–500 mM).

For purification of native TssA1^B^ (1–373), nucleic acids were removed from the clarified supernatant by precipitation with polyethyleneimine (0.15% (v/v)) at pH 8.0. After centrifugation at 40,000 *g* for 30 min at 4 °C, ammonium sulphate was added to the supernatant to a final concentration of 30% to precipitate TssA. The precipitated protein was recovered by centrifugation at 40,000 × *g* for 30 min at 4 °C and resuspended in a small volume of 50 mM Tris-Cl (pH 8.0) containing 50 mM NaCl, which was then applied onto a Superose 6 column equilibrated with the same buffer. SEC was performed at 0.5 ml/min flow-rate. TssA1^B^ eluted from the column as a single protein peak at ~13.75 min corresponding to a size of ~1.9 MDa.

### SEC

Size estimation of His_6_.TssA1^B^ Nt1 (1–255) was performed on a Superose 12 GL column (GE Healthcare) in 50 mM Tris-Cl (pH 8.0) containing 500 mM NaCl. The following standards were used (molecular weights in kDa): thyroglobulin, 669; apoferritin, 443; alcohol dehydrogenase, 149.5; conalbumin, 75; ovalbumin, 43; carbonic anhydrase, 29; ribonuclease, 13.7.

### SEC-MALLS

For size exclusion chromatography-multiangle laser light scattering (SEC-MALLS), native TssA1^B^ (1–373) and TssA1^B^ CTD (294–373) were purified as described above and analysed on a Superose-6 10/300 column in 10 mM phosphate buffer (pH 7.4) containing 2.7 mM KCl and 137 mM NaCl, whilst purified His_6_.TssA2^A^ (4.5 mg/ml)_,_ His_6_.TssA2^A^ Nt2-CTD (6.8 mg/ml) and His_6_.TssA2^A^ CTD (6.0 mg/ml), were filtered (0.2 µm pore size) and analysed on an S200 10/300 column (GE Healthcare) in 50 mM Tris.HCl (pH 8.0), 500 mM NaCl. These were carried out at ~20 °C using a system comprising a Dawn Heleos-II multi-angle light scattering detector, an Optilab rEX refractive index detector and a QELS Plus module (all items by Wyatt Technology) linked to a Shimadzu HPLC system (SPD-20A UV detector, LC20-AD isocratic pump system, DGU-20A3 degasser and SIL-20A autosampler) at the Molecular Interactions Lab, University of York, UK. Peak areas chosen for analysis were defined by Astra software (Wyatt Technology).

### Limited proteolysis

Twenty microlitres of purified His_6_.TssA2^A^ at 3.7 mg/ml in 16.7 mM Tris-HCl (pH 8.0), 175 mM NaCl was mixed with 1 μl of glutamyl endopeptidase (V8 protease or Glu-C, Sigma), glycyl endopeptidase (Gly-C, Protogen) and trypsin type I (Sigma) prepared at 1 mg/ml in water. The reaction was allowed to proceed for 90 min at room temperature and then stopped by addition of 20 μl 10% acetic acid. Digestion products were fractionated by 4–20% SDS-PAGE and blotted on to a PVDF membrane for N-terminal sequence analysis or analysed by mass spectrometry.

### Mass spectrometry and N-terminal analysis

Mass spectrometry (MS) analysis of TssA1^B^ and TssA2^A^ cleavage products in ultrapure water was carried out on an electrospray Q-TOF mass spectrometer at the Astbury Centre for Structural Molecular Biology, University of Leeds, UK. Samples were diluted into 50% acetonitrile/0.1% formic acid, and analysed by Z-spray nanoelectrospray ionisation MS using a quadrupole-IMS-orthogonal time-of-flight MS (Synapt HDMS, Waters UK Ltd., Manchester, U.K.) and in-house fabricated gold/palladium coated nanospray capillaries. The MS was operated in positive TOF mode using a capillary voltage of 1.2 kV, cone voltage of 20 V, nanoelectrospray nitrogen gas pressure of 0.1 bar, backing pressure of 2.47 mbar and a trap bias of 4 V. The source and desolvation temperatures were set at 80 °C and 150 °C, respectively. During TOF-MS acquisition, argon was used as the buffer gas, at a pressure of 4.0 × 10^–3^ mbar in the trap and transfer regions. Mass calibration was performed by a separate injection of sodium iodide at a concentration of 2 µg/µl. Data processing was performed using MassLynx v4.1 software.

Sequencing of TssA1^B^ and TssA2^A^ cleavage products was performed using an ABI Procise Sequencer operating standard Edman degradation procedures. After electrophoresis, proteins were electroblotted from the gel on to PVDF membrane (ABI ProBlott) using CAPS buffer at pH 11.0. Blotting was performed for 1–2 h at 450 mA with water cooling. Proteins were located on the membrane by staining with 1% Coomassie blue for 1 min, followed by destaining in 50% methanol. Transfer efficiency was verified by staining the gel after the transfer. The bands of interest were excised from the blot using a scalpel blade and transferred to a blot cartridge for sequence determination.

### Electron microscopy (EM)

0.75% (w/v) uranyl formate was used as a negative stain. Carbon-coated grids were glow discharged for 30 s in a Cressington 208 carbon coater system. Purified protein samples (~5 μl of 5–50 μg/ml in 50 mM Tris-HCl (pH 8.0), 200 mM NaCl, 10% glycerol (LinkerHis_6_.TssA1^B^ (1–373), His_6_.TssA2^A^ Nt2-CTD (223–478) and His_6_.TssA2^A^ CTD (381–478)) or 20 mM Tris-HCl (pH 7.4), 200 mM NaCl, 1 mM EDTA (TssA1^B^ CTD (294–373))) were incubated on the grids for 1 min. Excess protein sample was removed by blotting and the grid was then washed twice in distilled water and once in negative stain (1 s for each wash) before staining for 20 s. After final blotting to remove excess stain, the grids were vacuum dried and imaged on Philips CM100 transmission electron microscope equipped with a Gatan Mulitiscan 794 1k × 1k charge-coupled device camera, at nominal magnification 28500 and defocus between 500 and 900 nm or Philips CM200 FEG transmission electron microscope using a Gatan UltraScan 890 (US4000SP) 2k × 2k charge-coupled device camera, at a nominal magnification of 66,000× and defocus between −200 and −800 nm. Apart from the initial screening of His_6_.TssA2^A^ CTD and His_6_.TssA2^A^ Nt2-CTD, all other images were captured on the CM200 FEG. Alignments and averaging for EM analysis were carried out using the IMAGIC-5 software package^52,53^.

### X-ray crystallography

Native data on crystals of purified His_6_.TssA1^B^ Nt1 (1–255)^51^ were collected on beamline I02 at the Diamond Light Source, and 150° of data were processed in xia2–3d^54–57^ to 1.8 Å in spacegroup P2_1_2_1_2 (Supplementary Table 2). Crystals from the same conditions were subjected to sublimation of elemental iodine crystals overnight. Crystals were cryoprotected in 0.16 M calcium acetate, 0.08 M sodium cacodylate buffer (pH 6.5), 16.4% (w/v) PEG 8000 and 30% glycerol. Iodine SAD data were collected at 1.7 Å wavelength on beamline I03 at the Diamond Light Source, and data were processed in FastDP^58^ to 2.04 Å in spacegroup P2_1_2_1_2_1_ (Supplementary Table 2). The structure was solved using SHELX^59^ alone and as part of FastEP based on the identification of seven potential iodine sites. The subsequent poly-alanine model was partially refined and used to solve the structure of the 1.8 Å native data. This model then underwent rounds of model building in Coot^60^ and refinement using Refmac5^61,62^ to produce the final model (Supplementary Table 2). The asymmetric unit contains one molecule of His_6_.TssA1^B^ Nt1 (1–255), with eight N-terminal tag residues, 119 water molecules, two calcium ions and an ethylene glycol molecule.

Native data on crystals of purified TssA1^B^ CTD (303–373)^51^ and the selenomethionine derivative were collected on beamline I24 at the Diamond Light Source. Data were processed in xia2–3da^54–56,63,64^ (Supplementary Table 3) to 3.08 Å, in spacegroup I222. Selenomethionine peak SAD data were collected at 0.97922 Å wavelength on beamline I03 at the Diamond Light Source. Data were processed in xia2–3d^54–57^ (Supplementary Table 3) to 3.03 Å, in spacegroup I222. The SHELX program suite, SHELXC, D and E^59^, was used to identify 15 selenium sites and produce an initial electron density map. An initial model was built using Coot^60^, Buccaneer^65^ and employing a structure of a shorter Bc TssA1^B^ CTD derivative comprising residues 303–358 which had been solved separately, as a guide. This model was used as input to Phenix^66,67^, and subsequent output was subjected to rounds of building in Coot^60^, NCS averaging and solvent flattening in DM^68^. Refinement to the native data at 3.08 Å was performed using Refmac5^61,62^. Between 4 and 6 residues of the protein remain unmodelled at the C-terminus of each chain.

Purified His_6_.TssA1^B^ CTD H12–H14 (303–358), together with 12 additional residues from the tag, was set down in sitting-drop crystallisation trials and produced crystals in conditions 0.18 M tri-ammonium citrate, 20% (w/v) PEG 3350, which were cryoprotected in 0.2 M di-ammonium citrate, 22% (w/v) PEG 3350, 25% (v/v) ethylene glycol. Data were collected on beamline I02 at the Diamond Light Source, and processed in xia2 3dii^54–57,63,64^ to 2.35 Å in spacegroup P6 (Supplementary Table 4). The structure was solved in spacegroup P6_2_ by molecular replacement, in Phaser^69^, using the previously solved structure of TssA1^B^ CTD containing helices ɑ12 and ɑ13. The model was built and refined to native data at 2.35 Å in Coot^60^ and Refmac^61,62^, with two molecules in the asymmetric unit.

The structure of a TssA1^B^ CTD fragment corresponding to residues 303–347 was determined as follows. TssA1^B^ CTD (303–373), together with 4 additional residues from the tag, also produced crystals in condition 0.1 M citric acid (pH 5.0), 20% (w/v) PEG 6000 which were cryoprotected in 0.1 M citric acid (pH 5.0), 22% (w/v) PEG 6000, 25% (v/v) ethylene glycol. Native data were collected on beamline I04–1 at the Diamond Light Source, and processed in xia2 3daii^54–57,63,64^ to 1.78 Å in spacegroup I222 (Supplementary Table 5). Selenomethionine-incorporated MBP.TssA1^B^ CTD (303–373) was overexpressed and purified using a similar strategy to that described previously^51^. Crystals were produced in condition 0.1 M citric acid (pH 5.0), 20% (w/v) PEG 6000, and were cryoprotected in 0.1 M citric acid (pH 4.0), 22% (w/v) PEG 6000, 25% (v/v) glycerol. Selenomethionine MAD peak, inflection and high energy remote data were collected on beamline I03 at the Diamond Light Source and data were processed in xia2 3da or 3daii^54–57,63,64^ (Supplementary Table 5). The structure was solved by MAD using the SHELX program suite^59^, followed by building in Coot^60^ and refinement in Refmac5^61,62^ to the native data at 1.78 Å. Analysis of the electron density map strongly suggested that the protein had been clipped after W347.

To generate a derivative for crystals produced from purified His_6_.TssA2^A^ Nt2 (223–387)^33^, flakes of ethyl mercury phosphate powder were added to a drop containing crystals for 5 min, before subsequent cryoprotection in 0.04 M potassium phosphate monobasic, 16% (w/v) PEG 8000, 30% (v/v) glycerol. Hg SAD data were collected at 1.0088 Å wavelength on beamline I03 at the Diamond Light Source, and data were processed in xia2-DIALS^54–58^ to 2.16 Å in spacegroup P2_1_ (Supplementary Table 6). The structure of mercury bound Nt2 was solved using SHELX^59^ based on the identification of one mercury site for each of the two monomers in the asymmetric unit and built using Buccaneer and Coot^60,65^. Refinement was performed using Refmac5^61,62^

Purified TssA2^A^ Nt1-Nt2.His_6_ (1–374) containing a C-terminal LEHHHHHH tag crystallised in 0.2 M sodium chloride, 0.1 M HEPES buffer (pH 7.0), and 20% (w/v) PEG 6000. Crystals were cryoprotected in 0.2 M sodium chloride, 0.1 M HEPES buffer (pH 7.0), and 20% (w/v) PEG 6000 and 25% ethylene glycol. Native data were collected on beamline I04 at the Diamond Light Source, and data were processed in xia2-DIALS^54–58^ to 1.76 Å in spacegroup P2_1_. The structure of these crystals was solved by molecular replacement^69^ using the coordinates of the mercury bound TssA2^A^ Nt2 dimer as a search model. The model was iteratively built using Coot and refined to 1.8 Å using Refmac5^61,62^ to produce the final model (Supplementary Table 6). The asymmetric unit contains 4 molecules of TssA2^A^ Nt1-Nt2.His_6_ (231–374), and 380 water molecules. Despite the fact that the construct covered both the Nt1 and Nt2 domains the purified protein had undergone proteolytic cleavage leading to the loss of the Nt1 domain. The model therefore contains the entire Nt2 domain (residues 231–374).

Native data on crystals of His_6_.TssA2^A^ Nt2-CTD (223–478)^33^ were collected on beamline I03 at the Diamond Light Source. Data were processed in xia2-DIALS^54–56,58^ to 3.13 Å, in spacegroup P2_1_ (Supplementary Table 7). Molecular replacement^69^ using a search model of the TssA2^A^ Nt2 dimer identified the position of five copies of the TssA2^A^ Nt2 dimer in the asymmetric unit with additional density being seen for 10 copies of the CTD. An initial model was built using Coot^60^ and subsequently was subjected to rounds of re-building^60^, and NCS averaging with solvent flattening in Parrot^70^. Refinement to the native data at 3.13 Å was performed using Refmac5^61,62^ (Supplementary Table 7). Representative electron density for each deposited model is shown in Supplementary Fig. 10. All structures were validated using Molprobity^71^, with average B-factors calculated using Baverage^72^. Structural alignment was carried out with LSQKAB using secondary structure matching using Cα of the main chain^34^. PISA was used to calculate interface areas between molecules^32^. Structural homologues were discovered using the Dali structural recognition server^35^. Diagrams shown in this paper were generated using the PyMOL Molecular Graphics System, (Schrödinger, LLC).

### Bioinformatics

Phylogenetic analysis was carried out using MEGA 6^73^ and sequence alignment utilised Clustal Omega^74^. ESpript 3 was used for secondary structure mapping of conserved residues^75^.

## Electronic supplementary material

Supplementary information

Description of Additional Supplementary Files

Supplementary Data 1

## Data Availability

The X-ray crystal structures of TssA1^B^ Nt1, TssA1^B^ CTD, His_6_.TssA1^B^ CTD H12–H14, TssA1^B^ CTD fragment (303–347), TssA2^A^ Nt2, and TssA2^A^ Nt2-CTD and associated data have been deposited in the Protein Data Bank under the ID codes 6HS5 https://www.ebi.ac.uk/pdbe/entry/pdb/6hs5, 6HS6 https://www.ebi.ac.uk/pdbe/entry/pdb/6hs6, 6H8E https://www.ebi.ac.uk/pdbe/entry/pdb/6h8e, 6H8F https://www.ebi.ac.uk/pdbe/entry/pdb/6h8f, 6G7B https://www.ebi.ac.uk/pdbe/entry/pdb/6g7b and 6G7C https://www.ebi.ac.uk/pdbe/entry/pdb/6g7c, respectively. Other data that support the findings of the study are available from the corresponding author(s) upon request.
