# Supplementary material for: Structural insights into the function of type VI secretion system TssA subunits
Source: Nat Commun. 2018 Nov 12;9:4765. doi: 10.1038/s41467-018-07247-1 (PMC6232143; doi:10.1038/s41467-018-07247-1)
Supplement: Supplementary file 1 — Supplementary information [file 41467_2018_7247_MOESM1_ESM.pdf]

Supplementary Information

Structural insights into the function of type VI secretion system TssA subunits

Dix *et al*.

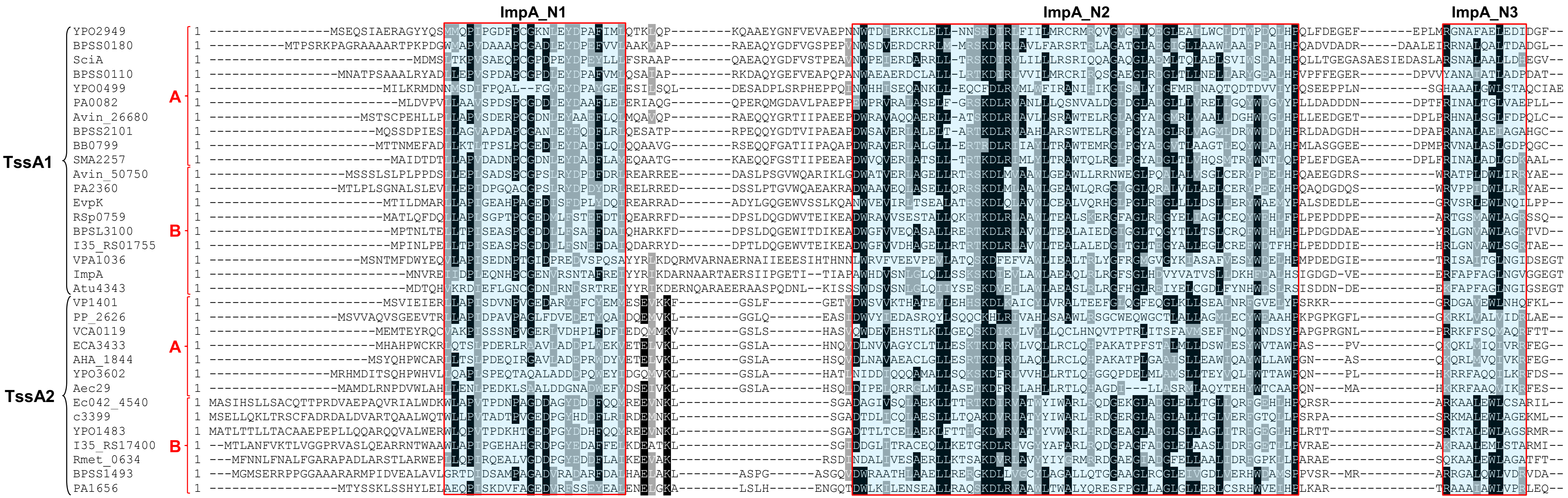

Supplementary Figure 1. Alignment of the ImpA_N region of TssA subunits. The N-terminal region of representative TssA1 and TssA2 subunits, corresponding to M1-E136 of *B. cenocepacia* TssA1^{B} (I35_RS01755), were aligned using Clustal Omega[1] and conserved residues were highlighted using Boxshade. Amino acids in white font with black shading are identical at the corresponding position in ≥ 50% of sequences aligned, whereas those highlighted in grey are similar. The three conserved motifs (ImpA_N1-ImpA_N3) that define the ImpA_N region are enclosed in red boxes with blue coloured fill. Members of the A and B sub-clades of each TssA family are indicated on the left of the alignment in red font. Gaps have been removed between non-alignable amino acids N-terminal to the ImpA_N1 motif to accommodate TssA sequences in a single line.

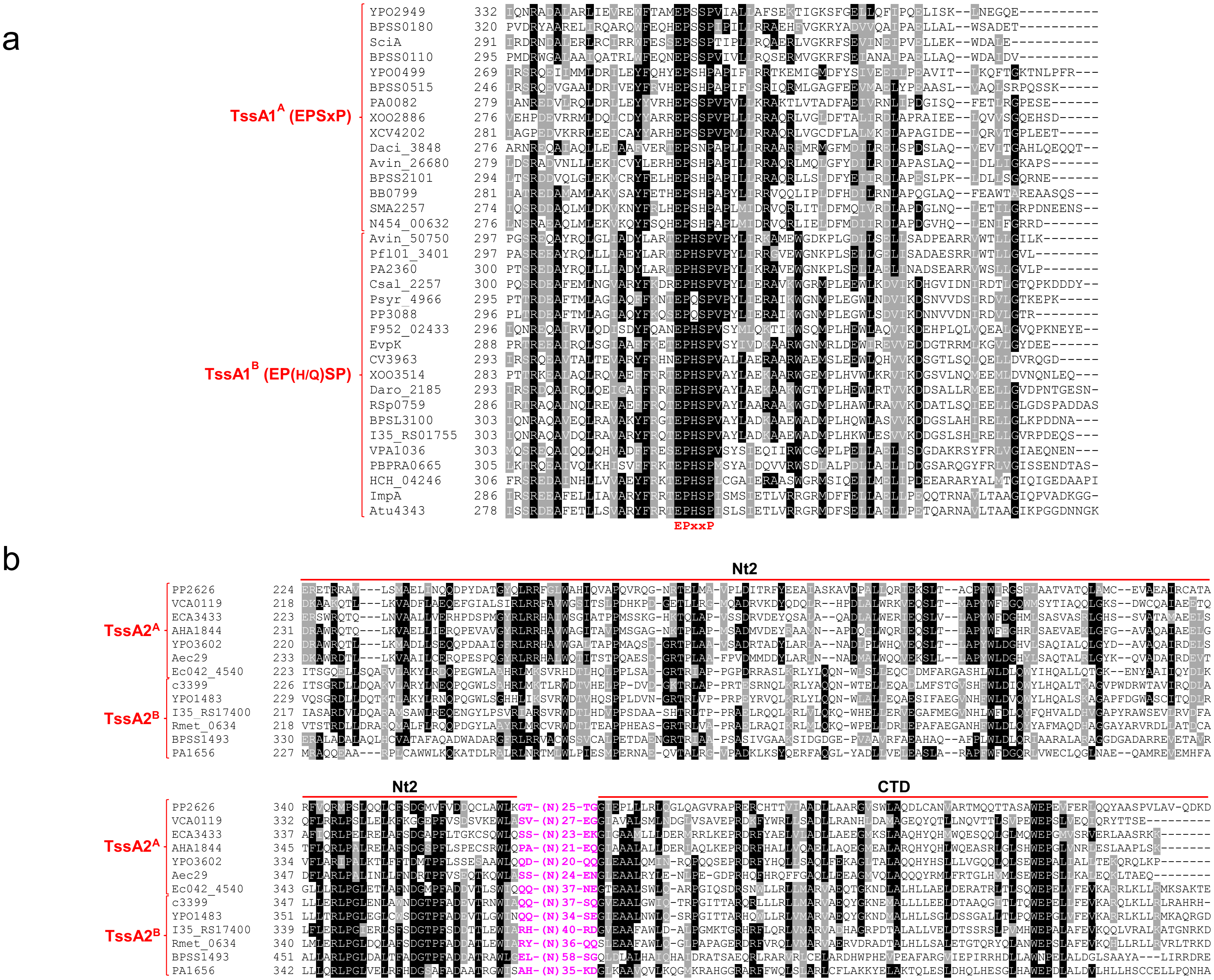

Supplementary Figure 2

Supplementary Figure 2. Alignment of C-terminal amino acid sequences of TssA1 and TssA2 subunits. Amino acid sequences of the C-terminal regions of representative TssA subunits were aligned using Clustal Omega[1] and conserved residues were highlighted using Boxshade. Amino acids in white font with black shading are identical at the corresponding position in ≥ 50% of sequences aligned, whereas those highlighted in grey are similar. **(a)** Alignment of TssA1 C-terminal domains. The CTDs of representative $TssA1^A$ and $TssA1^B$ orthologues are shown along with the last three residues of the predicted interdomain linker. The location of the conserved EPxxP motif is shown below the alignment. (**b**) Alignment of TssA2 Nt2 and C-terminal domains. Representative members of the A and B sub-clades, based on a phylogenetic analysis of full-length TssA2 subunits, are shown. Sequences shown in magenta are non-conserved and are predicted to constitute an interdomain linker connecting Nt2 to the CTD (the number of linker amino acids that are not shown is indicated). For clarity, the amino acid sequences at the extreme C-terminus of eight orthologues are not shown.

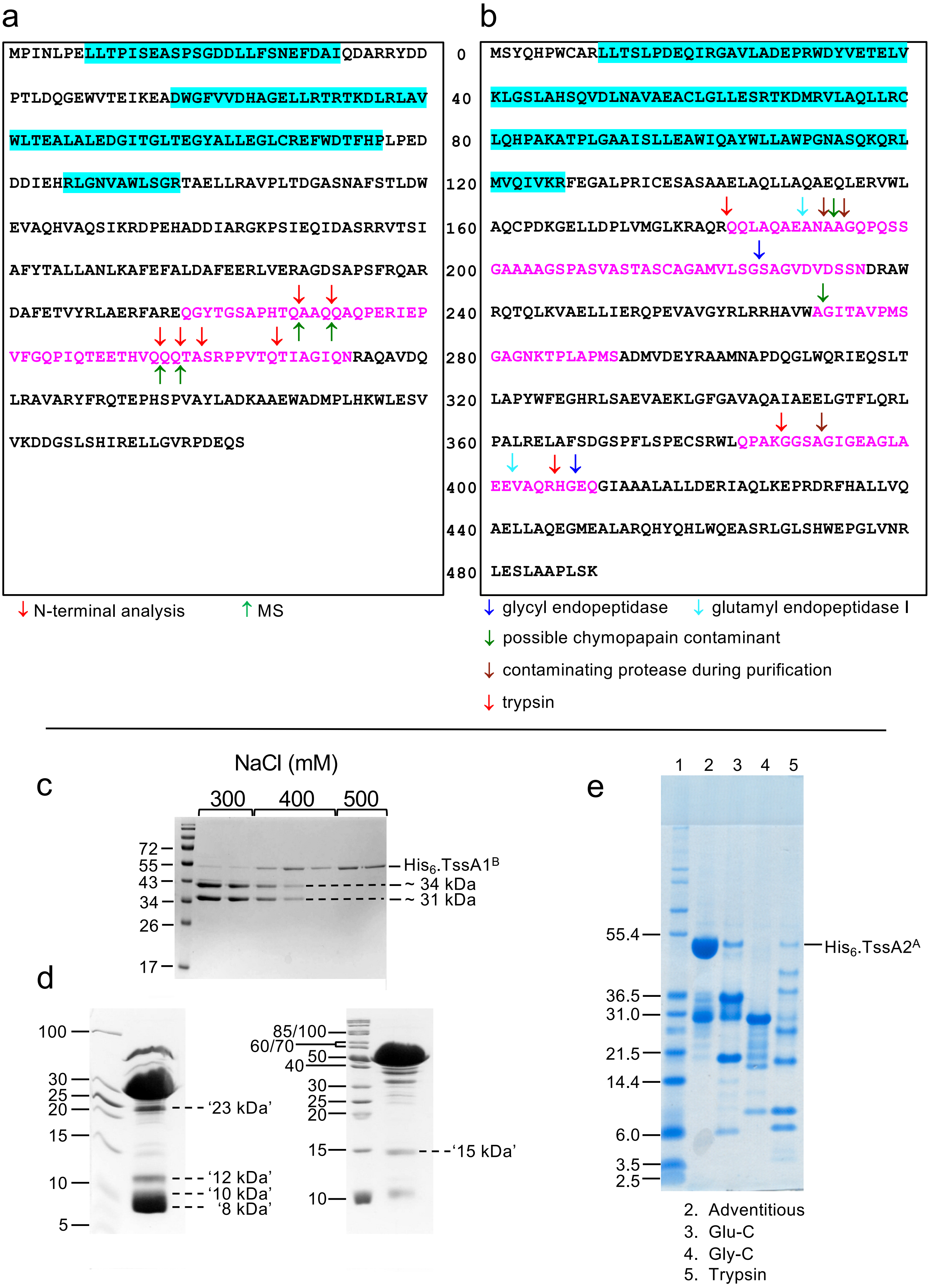

Supplementary Figure 3

Supplementary Figure 3. Domain analysis of TssA1^{B} and TssA2^{A}. Sequences of Bc TssA1^{B} (**a**) and Ah TssA2^{A} (**b**) with interdomain linkers shown in magenta font. (**a**) Adventitious protease cleavage sites utilised in interdomain linker identification are indicated by up and down arrows, for mass spectrometry and N-terminal analysis, respectively. (**b**) Arrows indicate protease specific cut sites within Ah TssA2^{A} identified by mass spectrometry that were used to identify the interdomain linker. (**c**) Analysis of large proteolytic degradation products of Bc TssA1^{B}. 12% SDS-PAGE analysis of His_6.TssA1^{B} following IMAC purification, dialysis and concentration, and application to a Q sepharose column in 20 mM Tris-HCl (pH 8.0) with elution by a NaCl step gradient, revealed two major species (~31 kDa and ~34 kDa), that corresponded to polypeptides of 31.3, 31.5, 34.2 and 34.4 kDa by mass spectrometry. Protein molecular weight markers are shown in kDa. (**d**) Analysis of small proteolytic degradation products of Bc TssA1^{B}. 15% SDS-PAGE analysis of His_6.TssA1^{B} degradation products before application to the Q column (left hand gel) and following elution (right hand gel), identified a series of low molecular weight peptides ~8, 10, 12, 15 and 23 kDa, these peptides were subjected to N-terminal amino acid sequence analysis. Protein molecular weight markers are shown in kDa. (**e**) Analysis of Ah TssA2^{A} proteolytic digestion products. SDS-PAGE analysis of His_6.TssA2^{A} following proteolytic digestion with contaminating protease (lane 2), glutamyl endopeptidase (Glu-C) (lane 3), glycyl endopeptidase (Gly-C) (lane 4), and trypsin (lane 5). Protein molecular weight markers are shown in kDa.

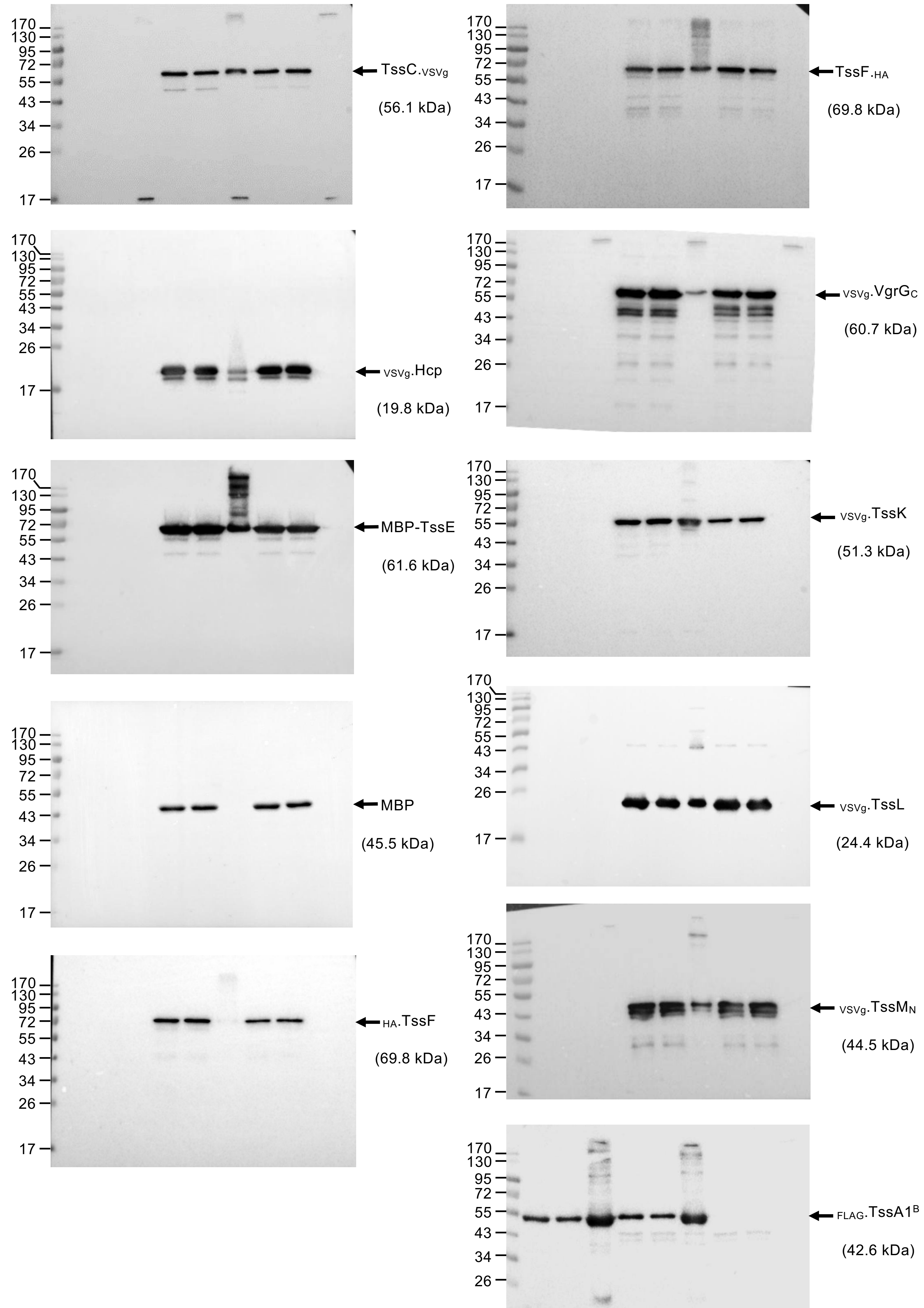

Supplementary Figure 4

Supplementary Figure 4. Co-immunoprecipitation analysis of FLAG-tagged Bc TssA1^{B} with other T6SS subunits. Uncropped images of western blots presented in **Fig. 2c** are shown. Molecular weights of the detected T6SS subunits (and control MBP) are indicated (which include any epitope, affinity or solubility tags) and markers are indicated in kDa. See legend to **Fig. 2** for more details.

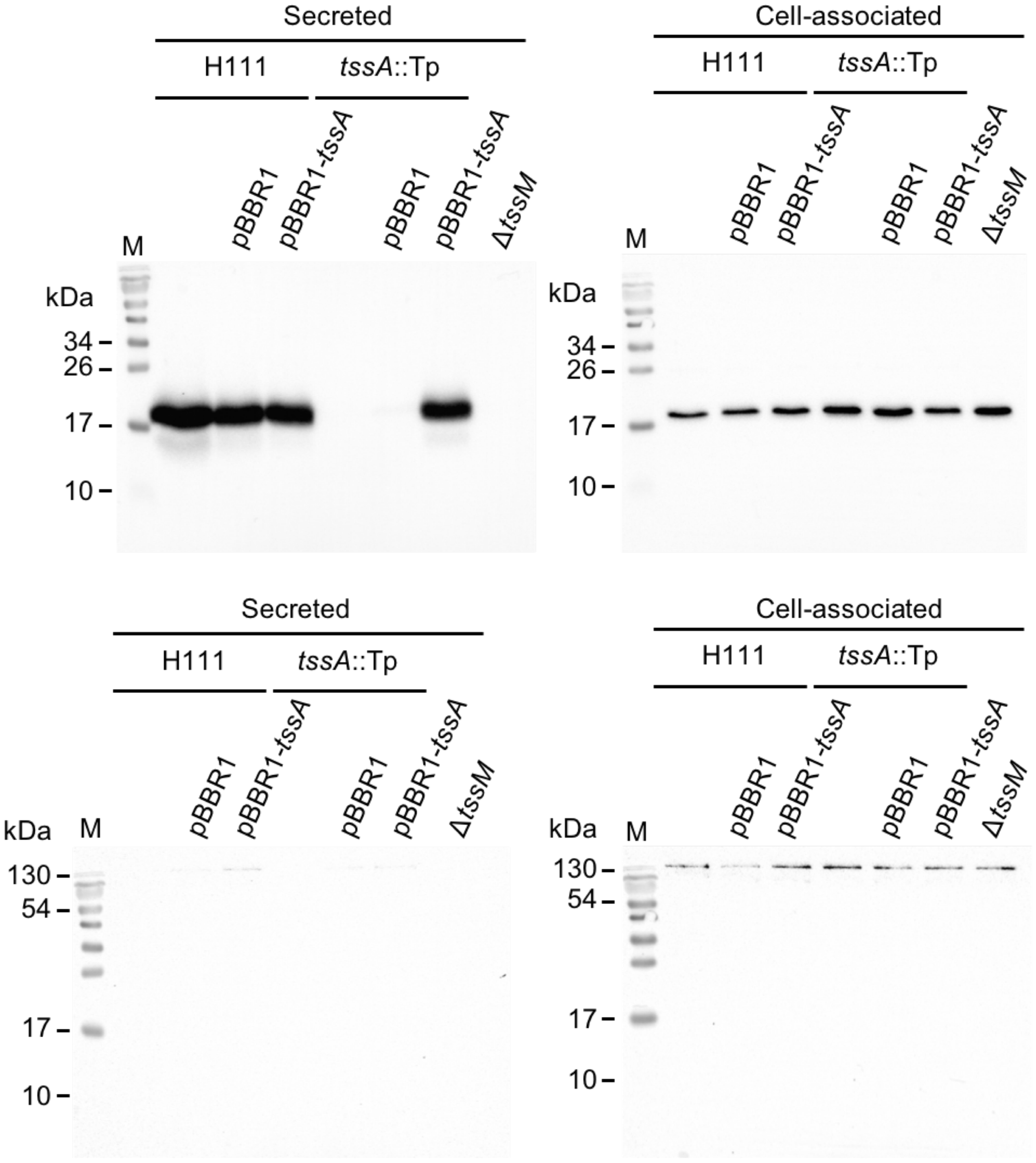

Supplementary Figure 5. Effect of TssA1^{B} inactivation on T6SS secretion activity. Uncropped images of western blots presented in **Fig. 3** are shown. See legend to **Fig. 3** for more details.

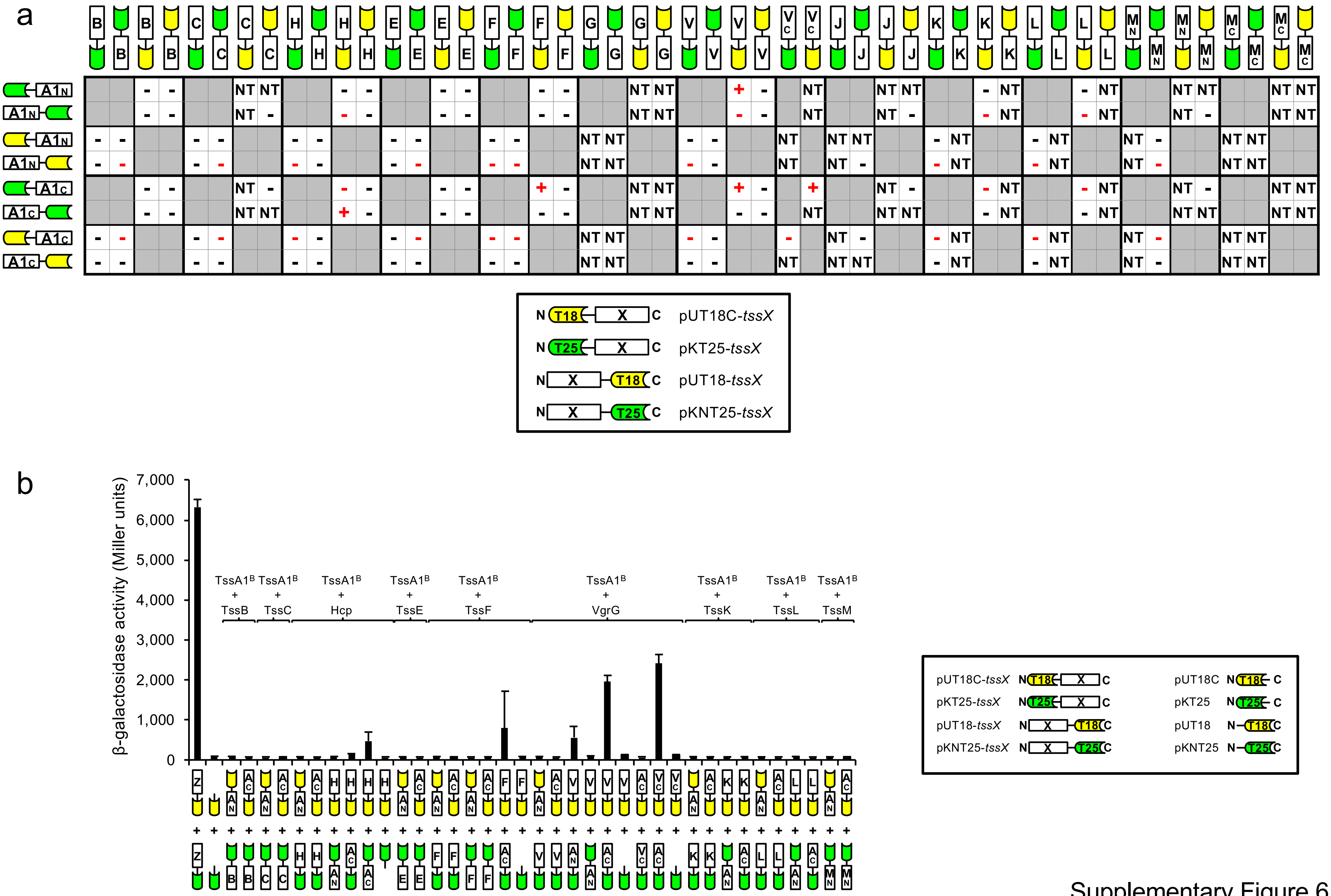

Supplementary Figure 6

Supplementary Figure 6. Two-hybrid analysis of TssA1^B domain interactions. (**a**) Each hybrid protein is represented by a green or yellow coloured motif indicating the CyaA fragment (T25 and T18, respectively) linked to a white rectangle labelled according to the T6SS subunit or TssA1^B domain. T6SS subunits are indicated by a single letter corresponding to the suffix used in the Tss nomenclature (i.e. B corresponds to TssB) except for H (Hcp), V (VgrG) and V_C (the VgrG core region, although only two hybrid proteins were constructed in this case). $A1_N$ and $A1_C$ correspond to TssA1^B NTD (Nt1) and CTD, respectively. Interactions of the cytoplasmic N- and periplasmic C-terminal regions of TssM (M_N and M_C, respectively) were investigated separately. Compatible pairwise combinations of BACTH plasmids encoding the indicated hybrid proteins were introduced into *E. coli* BTH101 and the resulting Mal phenotypes were scored on maltose-MacConkey agar. Colonies were recorded as +, positive or -, negative after 120 h incubation at 30 °C. Combinations involving TssG and TssM$_C$ that gave rise to a maltose-negative phenotype in combination with TssA1^B were not tested (NT). In most of the other cases only representative combinations were tested. Results shown in red font are from plasmid combinations that were quantitated by β-galactosidase assay and shown in (b). (**b**) β-galactosidase activities in *E. coli* BTH101 containing representative pairwise combinations of TssA1^B domain and T6SS subunit hybrid proteins were assayed. A_N, TssA1^B Nt1 (N-terminal 256 aa); A_C, TssA1^B CTD (C-terminal 80 aa); Z represents the Zip positive control. Otherwise, nomenclature is as in (a). Combinations involving TssG, TssJ and TssM$_C$ were not assayed as they do not appear to interact with TssA1^B. Data is representative of three independent experiments (n = 3) performed in duplicate and values correspond to the mean ± standard deviation. Values presented in **Supplementary Data 1**.

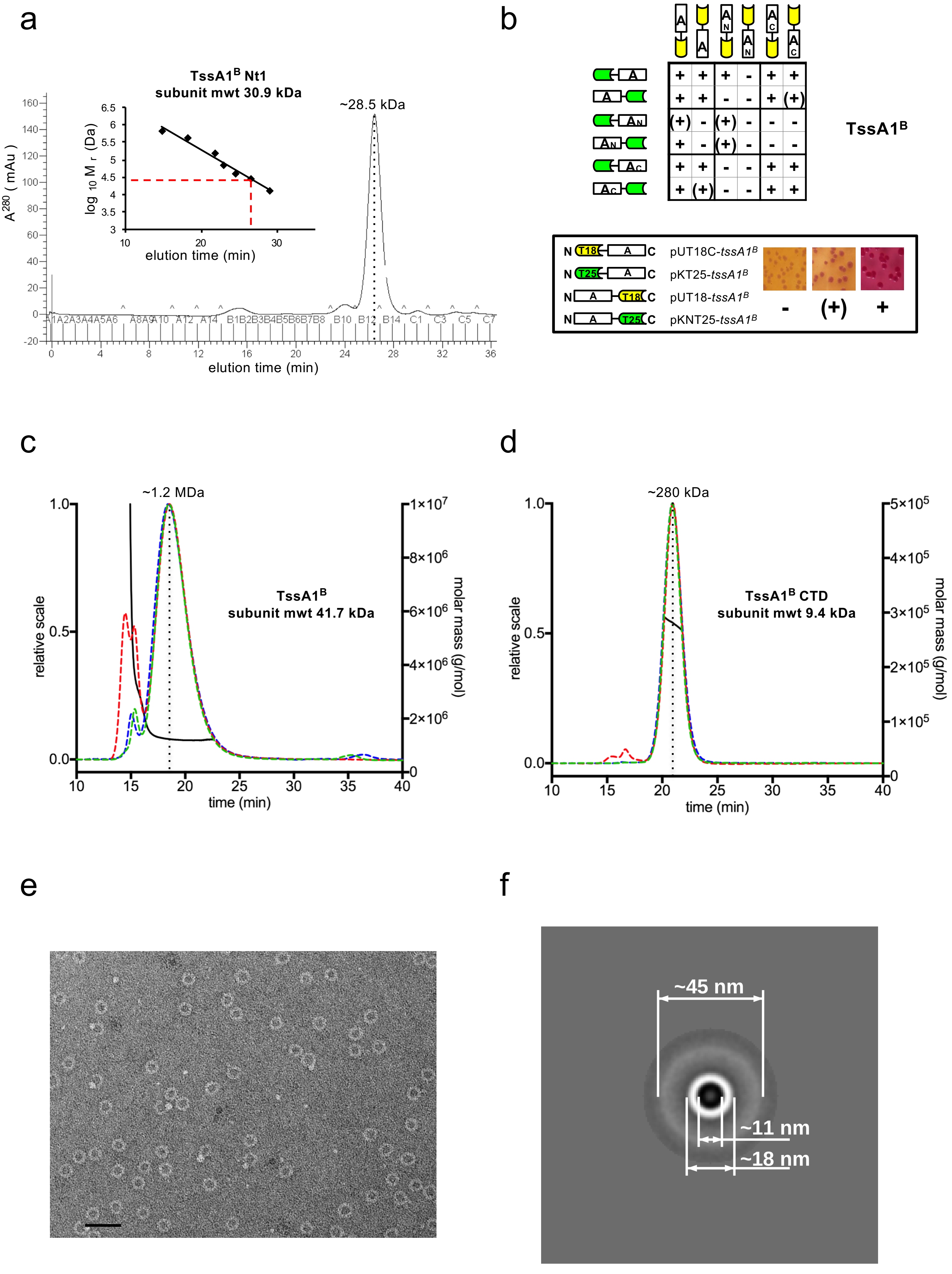

Supplementary Figure 7

Supplementary Figure 7. TssA1^B self-associates into a high molecular weight ring-like oligomer. (**a**) SEC profile of the Bc His_6.TssA1^B Nt1 domain indicating a monomer of 30.6 kDa is present. (**b**) Self-interaction analysis of Bc TssA1^B and its domains. Each hybrid protein is represented by a green or yellow coloured motif representing the CyaA fragment (T25 and T18, respectively) linked to a white rectangle labelled according to the fused region of TssA1^B (see key). All four possible hybrid proteins were constructed and analysed for TssA1^B and its domains in pairwise combinations (N, N-terminal domain (Nt1); C, C-terminal domain (CTD)). Colonies were recorded as +, positive; (+), weak positive; or -, negative (see key). (**c**) SEC-MALLS elution profile, peak positions, and molar mass plot of TssA1^B (~1.2 MDa). The normalised signals for light scattering (LS) are shown in red, refractive index (RI) in blue, UV absorption (280 nm) in green, and molecular weight estimate curve is shown in black. The molecular weight was determined by a Zimm fit procedure at each point. The X-axis is the elution time in minutes. (**d**) SEC-MALLS elution profile, peak positions, and molar mass plot of TssA1^B CTD (~280 kDa). Details as for (c). (**e**) Negative stain EM of single particle views of TssA1^B CTD. Scale bar is 50 nm. (**f**) Negative stain EM average of TssA1^B. Measurements indicate the inner diameter of the CTD ring (110 Å), the CTD ring outer diameter (180 Å), and diameter of the whole particle (450 Å).

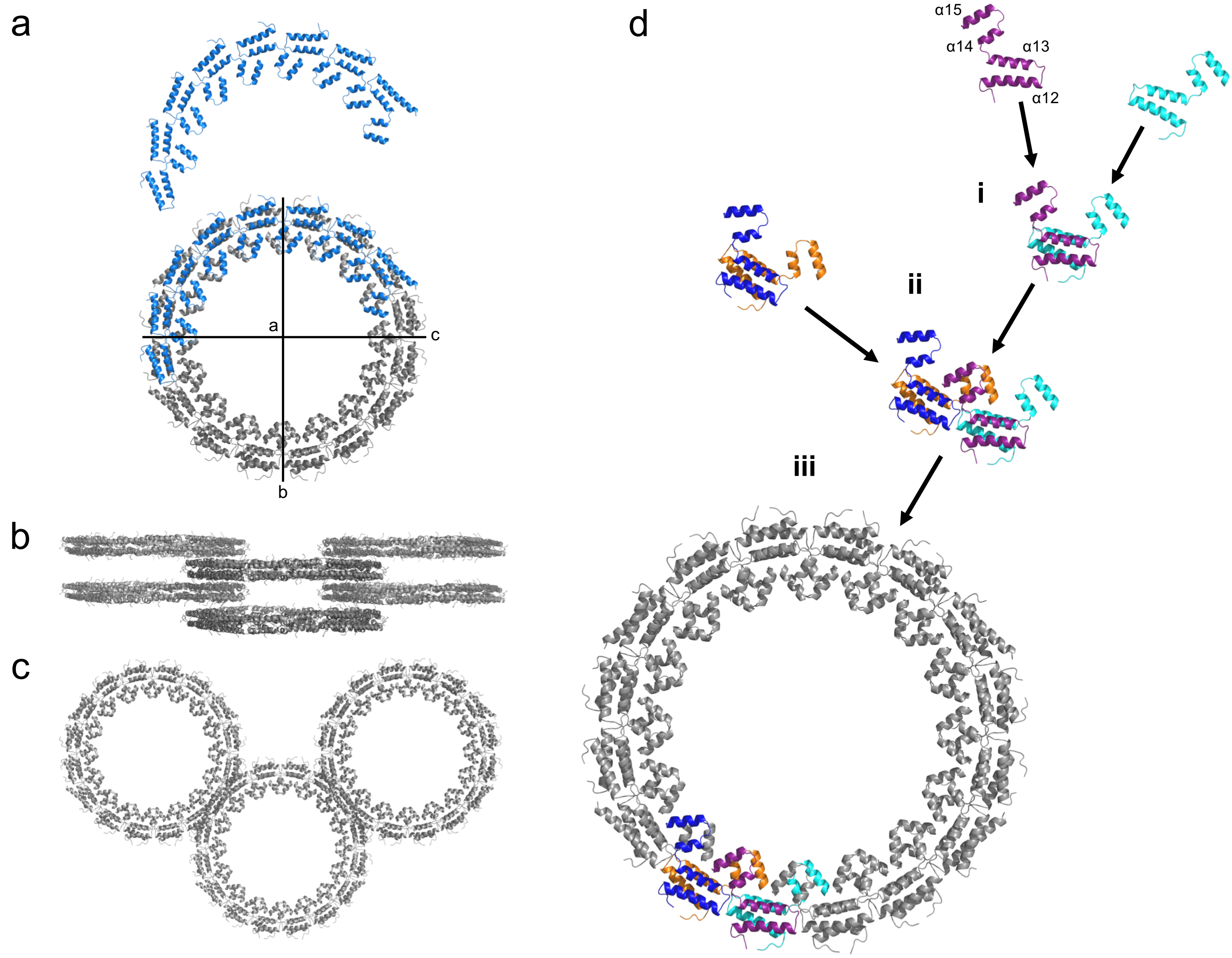

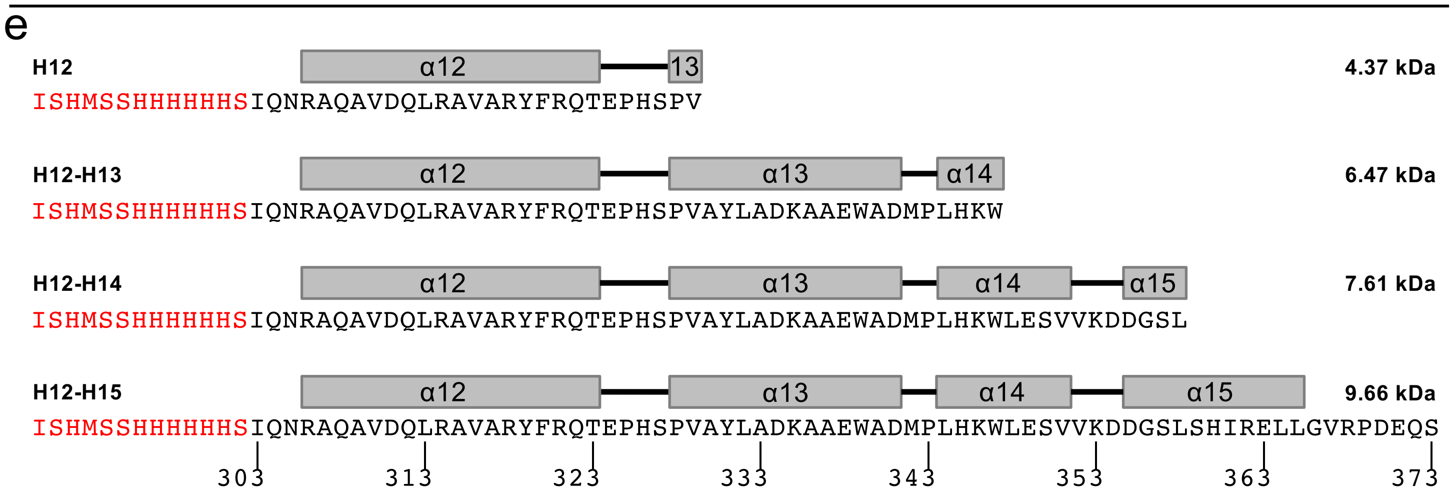

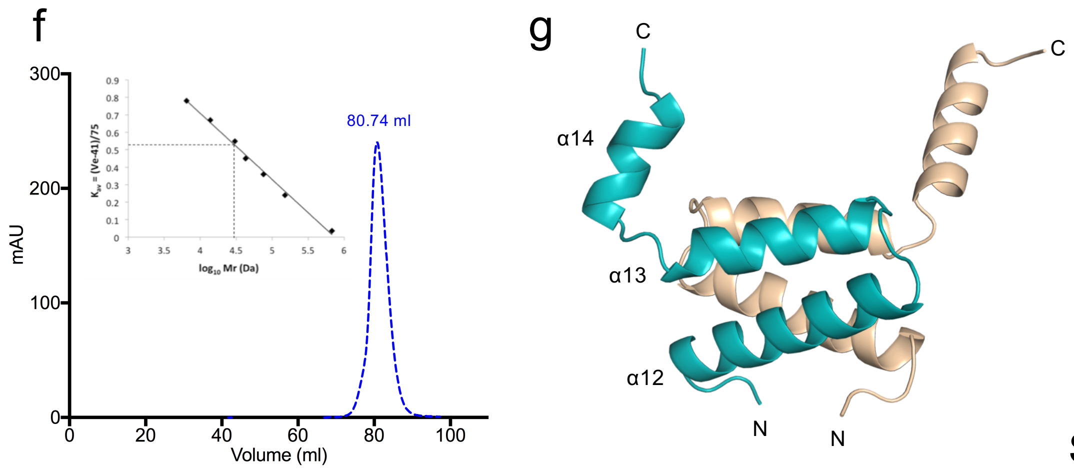

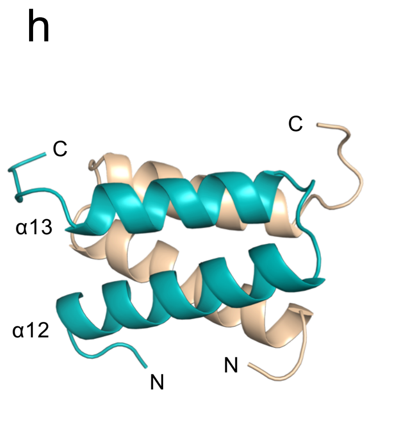

Supplementary Figure 8

Supplementary Figure 8. Assembly of the TssA1^B CTD ring oligomer. (a) Formation of the TssA1^B (I35_RS01755) CTD ring from the 8 subunits of the asymmetric unit. The contents of the asymmetric unit (blue) are related to the remaining sections of the ring (grey) by the 2-fold crystallographic symmetry axes around a, b and c. View is down the crystallographic axis a. **(b)** and **(c)** Packing of TssA1^B CTD ring oligomers in the crystal lattice. (b) View perpendicular to the 16-fold axis, (c) view parallel to the 16-fold axis. **(d)** Proposed assembly pathway for the TssA1^B CTD ring oligomer. (i) Two monomers (purple and cyan) dimerise, via helices α12 and α13; (ii) two dimers (purple/cyan and orange/blue) assemble with the majority of interactions occurring between helices α14 and α15 (purple and orange); (iii) sequential addition of further dimers (or higher order oligomers) results in assembly of a ring containing 32 subunits that exhibits D_{16} symmetry. **(e)** Nested deletion of TssA1^B CTD, containing an N-terminal MBP.His_6 tag. The sequences shown are released upon Factor Xa cleavage, with remaining His_6-tag residues highlighted in red. Construct H12-H15 corresponds to full-length TssA1^B CTD and was shown to form ring oligomers by EM, whereas the truncated derivatives form lower molecular weight complexes. **(f)** SEC profile of the H12-H14 construct on Superdex 200 (following removal of the MBP tag) to show failure to assemble ring oligomers. The elution peak is seen to shift from a high molecular weight structure ~355 kDa for selenomethionine-incorporated TssA1^B CTD[2], to a much lower molecular weight of ~30 kDa for the H12-H14 construct. SEC also showed that the MBP-tagged H12 construct formed monomers while MBP-tagged H12-H13 formed dimers. **(g)** Crystal structure of H12-H14 determined to 2.35 Å showing dimer formation. Residues 303-358 and 303-354 are resolved in chains A (wheat) and B (teal), respectively. **(h)** Crystal structure showing dimer formation by a truncated TssA1^B CTD derivative containing residues 303-347 (corresponding to helices α12 and α13) that arose from adventitious clipping of TssA1^B CTD following its release from MBP.TssA1^B CTD (303-373). Structure determined to 1.78 Å resolution.

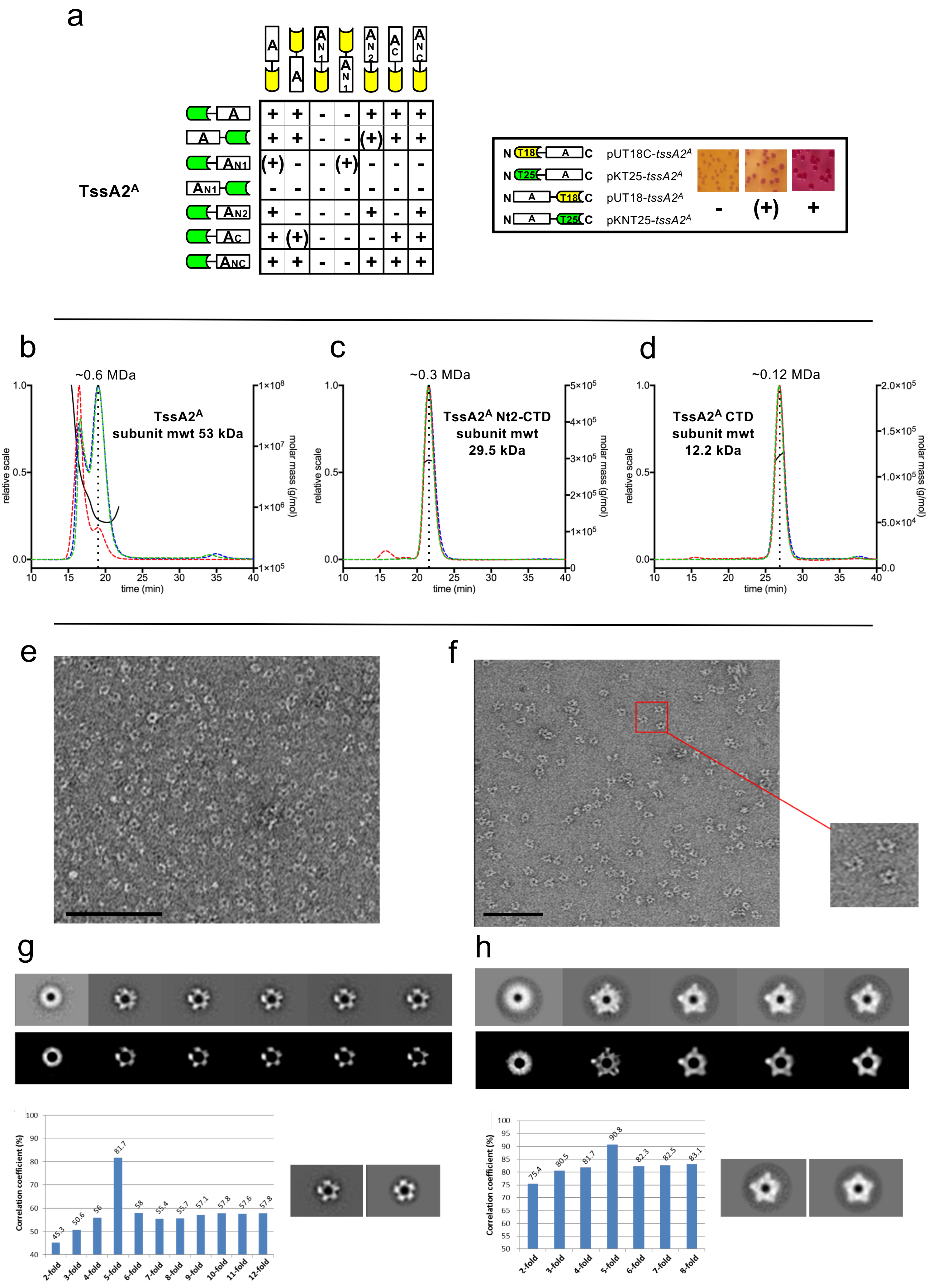

Supplementary Figure 9

Supplementary Figure 9. TssA2^A self-associates into a high molecular weight ring-like oligomer. (**a**) Self-interaction analysis of Ah TssA2^A and its domains. Hybrid proteins are represented as in Suppl. Fig. 6b All possible hybrid proteins were constructed and analysed for TssA2^A and its domains (N1, N-terminal domain (Nt1); N2, middle domain (Nt2); C, C-terminal domain (CTD); NC, Nt2-CTD region). (**b**)-(**d**) SEC-MALLS elution profile, peak positions, and molar mass plot of TssA2^A, TssA2^A Nt2-CTD and TssA2^A CTD, respectively. Details as for Supplementary Fig. 7c. (**e**) Negative stain EM of Ah TssA2^A CTD single particles. Scale bar is 100 nm. (**f**) Negative stain EM of Ah TssA2^A Nt2-CTD single particles, with a sample of individual particles selected in the magnified insert. Scale bar is 100 nm. (**g**)-(**h**) Class averaging and correlation co-efficient calculation for Ah TssA2^A CTD and Ah TssA2^A Nt2-CTD particles, respectively. The x-axis shows the specific symmetry that was tested, and the y-axis shows the correlation coefficient (%) of all the single particles rotated at a specific angle according to the fold of symmetry, then correlated to their unrotated self.

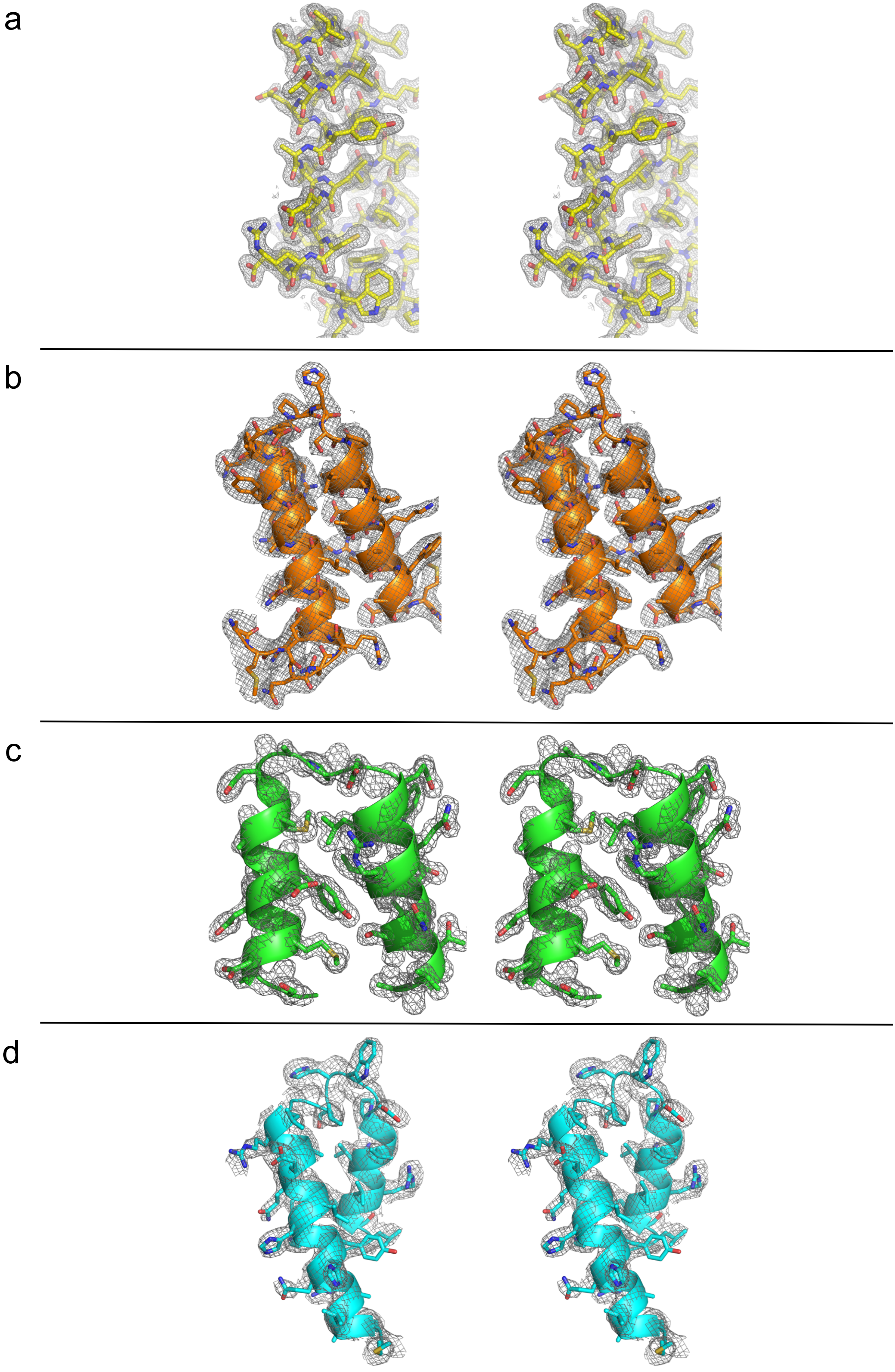

Supplementary Figure 10

Supplementary Figure 10. Electron density maps for TssA1^B Nt1, TssA1^B CTD, TssA2^A Nt2 and TssA2^A CTD. (**a**) Electron density visible for residues K72-P115 of helices α4-α5 of TssA1^B Nt1. (**b**) Electron density visible for tag residues H301-M302 and residues I303-W347 of helices α12-α13 of TssA1^B CTD. (**c**) Electron density visible for residues M284-P315 of helices α3-α4 of the TssA2^A Nt2 structure**.** (**d**) Electron density visible for residues M438-A474 of helices α11-α12 of the TssA2^A CTD structure. All 2Fo-Fc maps contoured to 1.0 σ RMSD.

Supplementary Table 1. N-terminal amino acid sequence of low mol. wt. His_6.TssA1^B degradation products

Peptide mol. wt.[a]	N-terminal sequence
23 kDa	G-S-S-H-H-H-H-[b]
	H-A-D-X-X-X-
15 kDa	A-A-Q-Q-A-Q-P-E
	Q-A-Q-P-E-X-I-E-
12 kDa	K-R-D-P-E-H-A-D-D-
	R-D-P-E-H-A-D-D-
10 kDa	Q-Q-T-A-S-R-P-
	T-A-S-R-P-P-V-
	S-R-P-P-V-T-
8 kDa	T-I-A-G-I-Q-N-

[a]Sizes were estimated based on migration in SDS PA gels.
[b]Corresponds to the N-terminus of His_6.TssA1^B following removal of the N-terminal methionine.

Supplementary Table 2. Data collection and refinement statistics for His$_6$.TssA1^B Nt1 (1-255)

	His$_6$.TssA1^B Nt1 (1-255) Native	His$_6$.TssA1^B Nt1 (1-255) Peak - Iodine
**PDB code**	**6HS5**	
**Data collection**[a]		
Wavelength (Å)	1.04434	1.70000
Space group	$P2_12_12$	$P2_12_12$
Cell dimensions		
a, *b*, *c* (Å)	49.7, 125.7, 45.6	49.8, 125.5, 45.6
α, β, γ (°)	90.0, 90.0, 90.0	90.0, 90.0, 90.0
Resolution range (Å)	49.67-1.80 (1.85-1.80)	29.64-2.04 (2.09-2.04)
R_{merge}	0.043 (0.344)	0.169 (0.865)
$I / \sigma I$	21.2 (4.4)	19.3 (2.8)
Completeness (%)	99.8 (99.0)	98.1 (74.2)
Redundancy	5.4 (5.3)	23.0 (15.8)
No. total reflections	146307 (10493)	427376 (15999)
No. unique reflections	27244 (1966)	18554 (1011)
**Refinement**		
Resolution range (Å)	46.2-1.80	
No. reflections (Work/Free)	25817/1381	
R_{work} / R_{free}	0.177/0.226	
No. atoms		
Protein	1991	
Ligand/ion[b]	6	
Water	119	
B-factors (Å^2)		
Protein	30.6	
Ligand/ion[b]	22.5/20.5	
Water	29.4	
R.m.s. deviations		
Bond lengths (Å)	0.0157	
Bond angles (°)	1.5665	
Ramachandran favoured (%)[c]	98.79	
Ramachandran outliers (%)[c]	0.00	

The number of crystals for the His$_6$.TssA1^B Nt1 (1-255) structure is 1 per data set
[a]Values in parentheses are for highest-resolution shell.
[b]1 x ethylene glycol/2 x calcium ion
[c]Calculated using MolProbity[3]

Supplementary Table 3. Data collection and refinement statistics for TssA1[B] CTD (303-373)

	TssA1[B] CTD (303-373) Native	TssA1[B] CTD (303-373) Peak - Selenomethionine
**PDB code**	**6HS6**	
**Data collection**[a]		
Wavelength (Å)	1.70001	0.97922
Space group	I222	I222
Cell dimensions		
a, *b*, *c* (Å)	46.3, 201.7, 263.7	46.0, 200.3, 263.0
α, β, γ (°)	90.0, 90.0, 90.0	90.0, 90.0, 90.0
Resolution range (Å)	41.49-3.08 (3.16-3.08)	100.12-3.03 (3.11-3.03)
R_{merge}	0.131 (0.786)	0.220 (2.144)
$I / \sigma I$	17.5 (3.7)	9.8 (1.4)
Completeness (%)	99.9 (100.0)	99.9 (100.0)
Redundancy	12.6 (11.9)	12.9 (13.4)
No. total reflections	297122 (20394)	315386 (23629)
No. unique reflections	23602 (1709)	24385 (1759)
**Refinement**		
Resolution range (Å)	41.49-3.08	
No. reflections (Work/Free)	22387/1215	
R_{work} / R_{free}	0.198/0.242	
No. atoms		
Protein	4401	
Ligand/ion	0	
Water	0	
B-factors ($Å^2$)		
Protein	70.2	
Ligand/ion	-	
Water	-	
R.m.s. deviations		
Bond lengths (Å)	0.0114	
Bond angles (°)	1.6210	
Ramachandran favoured (%)[b]	96.05	
Ramachandran outliers (%)[b]	0.00	

The number of crystals for the TssA1[B] CTD (303-373) structure is 1 per data set

[a]Values in parentheses are for highest-resolution shell.

[b]Calculated using MolProbity[3]

Supplementary Table 4. Data collection and refinement statistics for His$_6$.TssA1^B CTD H12-H14 (303-358)

	His$_6$.TssA1^B CTD H12-H14 (303-358)
**PDB code**	**6H8E**
**Data collection**[a]	
Wavelength (Å)	0.97943
Space group	$P6_2$
Cell dimensions	
a, *b*, *c* (Å)	65.3, 65.3, 66.1
α, β, γ (°)	90.0, 90.0, 120.0
Resolution range (Å)	42.97-2.35 (2.41-2.35)
R_{merge}	0.094 (0.713)
$I / \sigma I$	17.1 (3.1)
Completeness (%)	99.9 (99.4)
Redundancy	8.5 (8.1)
No. total reflections	57299 (4002)
No. unique reflections	6747 (492)
**Refinement**	
Resolution range (Å)	42.97-2.35
No. reflections (Work/Free)	6372/357
R_{work} / R_{free}	0.193/0.237
No. atoms	
Protein	897
Ligand/ion	0
Water	0
B-factors (Å^2)	
Protein	45.1
Ligand/ion	-
Water	-
R.m.s. deviations	
Bond lengths (Å)	0.0129
Bond angles (°)	1.4320
Ramachandran favoured (%)[b]	99.06
Ramachandran outliers (%)[b]	0.00

The number of crystals for the His$_6$.TssA1^B CTD (303-358) structure is 1

[a]Values in parentheses are for highest-resolution shell.

[b]Calculated using MolProbity[3]

Supplementary Table 5. Data collection, phasing and refinement statistics for TssA1^{B} CTD (303-373) fragment

	TssA1^{B} CTD (303-373) fragment	TssA1^{B} CTD (303-373) fragment - Selenomethionine		
**PDB code**	**6H8F**			
**Data collection**[a]				
		Peak	*Inflection*	*Remote*
Wavelength (Å)	0.92000	0.97934	0.97949	0.96411
Space group	I222	I222	I222	I222
Cell dimensions				
a, *b*, *c* (Å)	48.3, 62.7, 65.7	47.8, 62.6, 65.6	47.8, 62.8, 65.8	47.7, 62.9, 65.7
α, β, γ (°)	90.0, 90.0, 90.0	90.0, 90.0, 90.0	90.0, 90.0, 90.0	90.0, 90.0, 90.0
Resolution range (Å)	38.90-1.78 (1.83-1.78)	16.79-1.71 (1.75-1.71)	38.64-1.87 (1.92-1.87)	38.61-2.04 (2.09-2.04)
R_{merge}	0.068 (0.697)	0.059 (0.512)	0.065 (0.739)	0.072 (0.703)
$I / \sigma I$	13.2 (2.0)	21.4 (2.7)	22.0 (2.9)	21.7 (4.0)
Completeness (%)	97.0 (99.3)	97.9 (83.9)	100.0 (100.0)	100.0 (100.0)
Redundancy	4.5 (4.6)	11.2 (5.6)	12.5 (9.6)	12.7 (13.1)
No. total reflections	42707 (3238)	120101 (3596)	105843 (5954)	83404 (6305)
No. unique reflections	9502 (700)	10692 (644)	8487 (618)	6577 (482)
**Refinement**				
Resolution (Å)	38.90-1.78			
No. reflections (Work/Free)	9048/454			
R_{work} / R_{free}	0.189/0.237			
No. atoms				
Protein	752			
Ligand/ion	0			
Water	39			
B-factors (Å^2)				
Protein	27.6			
Ligand/ion	-			
Water	27.6			
R.m.s deviations				
Bond lengths (Å)	0.0117			
Bond angles (°)	1.3164			
Ramachandran favoured (%)[b]	100.00			
Ramachandran outliers (%)[b]	0.00			

The number of crystals for the TssA1^{B} CTD (303-373) fragment structure is 1 per data set
[a]Values in parentheses are for highest-resolution shell.
[b]Calculated using MolProbity[3]

Supplementary Table 6. Data collection and refinement statistics for TssA2[A] Nt1-Nt2.His$_6$ (1-374) and TssA2[A] His$_6$.Nt2 (231-374)

	TssA2[A] Nt1-Nt2.His$_6$ (1-374)	TssA2[A] His$_6$.Nt2 (231-374) - Mercury
**PDB code**	**6G7B**	
**Data collection**[a]		
Wavelength (Å)	0.9795	1.0088
Space group	$P2_1$	$P2_1$
Cell dimensions		
a, *b*, *c* (Å)	39.8, 101.3, 78.4	45.8, 40.0, 100.5
α, β, γ (°)	90.0, 104.6, 90.0	90.0, 102.5, 90.0
Resolution range (Å)	42.13-1.76 (1.79-1.76)	44.71-2.16 (2.23-2.16)
R_{merge}	0.065 (0.611)	0.162 (1.492)
$I / \sigma I$	7 (1.2)	9.4 (1.2)
Completeness (%)	99.9 (98.7)	95.9 (71.9)
Redundancy	3.6 (3.3)	12.4 (8.6)
No. total reflections	214716 (9711)	231080 (8786)
No. unique reflections	59629 (2971)	18671 (1026)
**Refinement**		
Resolution range (Å)	101.32-1.76	
No. reflections (Work/Free)	56717/2805	
R_{work} / R_{free}	0.272/0.306	
No. atoms		
Protein	4584	
Ligand/ion	0	
Water	380	
B-factors (Å^2)		
Protein	22.9	
Ligand/ion	-	
Water	29.3	
R.m.s. deviations		
Bond lengths (Å)	0.0175	
Bond angles (°)	1.702	
Ramachandran favoured (%)[b]	98.8	
Ramachandran outliers (%)[b]	0.00	

The number of crystals for the TssA2[A] Nt1-Nt2.His$_6$ (1-374) structure is 1 per data set
[a]Values in parentheses are for highest-resolution shell.
[b]Calculated using MolProbity[3]

Supplementary Table 7. Data collection and refinement statistics for TssA2[A] His$_6$.Nt2-CTD (223-478)

	TssA2[A] His$_6$.Nt2-CTD (223-478)
**PDB code**	**6G7C**
**Data collection**[a]	
Wavelength (Å)	0.97623
Space group	$P2_1$
Cell dimensions	
a, *b*, *c* (Å)	70.0, 202.1, 139.0
α, β, γ (°)	90.0, 90.7, 90.0
Resolution range (Å)	139.00-3.13 (3.21-3.13)
R_{merge}	0.113 (1.368)
$I / \sigma I$	5.8 (0.9)
Completeness (%)	99.9 (100.0)
Redundancy	3.7 (3.6)
No. total reflections	253133 (18358)
No. unique reflections	67977 (5030)
**Refinement**	
Resolution range (Å)	202.08-3.13
No. reflections (Work/Free)	64474/3380
R_{work} / R_{free}	0.255/0.328
No. atoms	
Protein	18363
Ligand/ion	0
Water	0
B-factors (Å^2)	
Protein	99.3
Ligand/ion	-
Water	-
R.m.s. deviations	
Bond lengths (Å)	0.011
Bond angles (°)	1.60
Ramachandran favoured (%)[b]	90.5
Ramachandran outliers (%)[b]	1.35

The number of crystals for the TssA2[A] His$_6$.Nt2-CTD (223-478) structure is 1
[a]Values in parentheses are for highest-resolution shell.
[b]Calculated using MolProbity[3]

Supplementary Table 8. Bacterial strains

Strain	Genotype/Description[a]	Source or reference
B. cenocepacia		
H111	CF isolate, prototroph	(4)
H111ΔtssM	H111 containing an in-frame deletion within *tssM*	This study
H111-tssA::Tp	H111 containing a Tp^R cassette insertion within *tssA*	This study
A. hydrophila		
ATCC 7966	Wild type. Prototroph.	(5)
E. coli		
JM83	F^- *ara* Δ(*lac-proAB*) *rpsL* ϕ80d*lacZΔM15* (Sm^R)	(6)
DH5α	F^- *recA1 endA1 gyrA96 thi-1 hsdR17* ($r_K^- m_K^+$) *supE44 relA1 Δ(lacZYA-argF)U169 deoR phoA* λ^- ϕ80d*lacZΔM15*	(7)
S17-1(λpir)	*thi proA hsdR recA* RP4-2-*tet*::Mu-1 *kan*::Tn*7* integrant λpir (Tp^R Sm^R)	(8)
SM10(λpir)	*thi-1 thr leu tonA lacY supE recA* RP4-2-Tc::Mu λpir (Km^R)	(8)
BTH101	F^-, *cya-99 araD139 galE15 galK16 rpsL1* (Sm^R) *hsdR2 mcrA1 mcrB1*	(9)
BL21(DE3)	F^- *ompT* $hsdS_B$ (r_B^- m_B^+) *dcm gal* λDE3	(10)
ER2523	*fhuA2* [*lon*] *ompT gal sulA11 (mcr-73*::mini-Tn*10)2* (Tc^S) [*dcm*] *zgb-210*::Tn*10* (Tc^S) *endA1 Δ(mcrC-mrr)114*::IS*10*	New England Bio-labs

[a]Km^R, kanamycin resistant; Rf^R, rifampicin resistant; Sm^R, streptomycin resistant; Tc^S, tetracycline sensitive; Tp^R, trimethoprim resistant.

Supplementary Table 9. Oligonucleotides

Oligo/Primer name	Sequence (5'-3')[a,b,c]
**Vector modification**	
delacO1for	GATCACGGGATCTCGACGCTCTCCCTATTAGGAAATTAATACG
delacO1for2	ACTCACTATAGGGACCTGTAGAAATAATTTTGTTTAACTTTAATAAGGAGATATAC
delacO1rev	CATGGTATATCTCCTTATTAAAGTTAAACAAAATTATTTCTACAGGTCC
delacO1rev2	CTATAGTGAGTCGTATTAATTTCCTAATAGGGAGAGCGTCGAGATCCCGT
delacO2for	GTACACGGCCGCATAATCGAAATTAATACGACT
delacO2for2	CACTATAGGGACCATCTTAGTATATTAGTTAAGTATAAGAAGGAGATATACA
delacO2rev	TATGTATATCTCCTTCTTATACTTAACTAATAT
delacO2rev2	ACTAAGATGGTCCCTATAGTGAGTCGTATTAATTTCGATTATGCGGCCGT
pMALHisFor	GCGCCAATTGACCAACAAGGACCATAGATTATGAAAATC*CATCACCATCACCATCAC*GAAGAAGGTAAACTGGTAATCT
pMALHisRev	TGGCGGGTTCGGCAGCAGATCT
**Construction of *tssA1*^{B} mutant**	
iotAfor	GCGCAAGCTTCACGCGACATCTCATGCATC
iotAfor3	GCGCAAGCTTCCGCGGAAATTCGACGCGATCCAGGAC
iotArev	GCGCGGATCCCCCGTCTTGCGTTTACGACT
iotArev2	ATCACGAAGAGCATTCCGCC
**Construction of *tssM* mutant**	
tssMfor	GCGCTCTAGAGGAACCTGAACGTCCTATGC
tssMrev2	GCGCGGTACCTCATTGCGCCCTCTGTGCAT
tssM-OPfor	TCATCCCGTTTGACAGCATG
tssM-OPrev2	ATCTTGCCGAAGTAGGCGATTT
**Subunit expression for purification**	
TssA1^{B}	

tssAnonHisFor	GCGCTCTAGAAATAATTTTGTTTAACTTTAAGAAGGAGATATACCATGCCGATCAATCTCCCCGA
pET14b-iotAfor	GCGCCATATGCCGATCAATCTCCCCGA
pET14b-iotArev	GCGCGGATCCTGCGTTTACGACTGCTCGTC
TssAlinkerHis1for	GCG*CATCACCATCACCATCAC*GCGCAGCAGGCCCAGCCCGA
TssAlinkerHis1rev	CGC*GTGATGGTGATGGTGATG*CGCCTGCGTGTGCGGCGCGC
pET14b-ACTDfor	GCGCCATATGCCTCCGGTGACGCAGACGAT
pACYC-tssA.CTDfor	GCGCCATATGAGCAGC*CATCATCATCATCATCAC*AGCATCCAGAACCGTGCGCAGGC
pACYC-tssA.CTDH1rev2	GCGCGGATCCTTAGACCGGGCTGTGCGGCTCGG
pACYC-tssA.CTDH2rev2	GCGCGGATCCTTACCACTTGTGCAGCGGCATGT
pACYC-tssA.CTDH3rev2	GCGCGGATCCTTACAGCGAGCCGTCGTCCTTCA
TssA2[A]	
pET21a-AHA1844.Nt1Nt2.for	ATATATACATATGAGCTATCAACACCCCTGGTGTGCAC
pET21a-AHA1844.Nt1Nt2.rev	TATATTATATACTCGAGCAGCCAGCGGCTGCA
pACYC-AHA1844.CTDSD2for	GCGCGGATCCGAGTGCGGGCATTGGCGAGGC
pACYC-AHA1844.rev	GCGCAAGCTTTCATTTCGACAACGGCGCCG
TssD	
pET-His-iotDfor2	TATGCGAAGGGCCATGTTACAT
pET14b-iotDrev	GCGCGGATCCTTAGACCGCGTAGGTCTT
**Subunit expression for co-IP**	
TssA.NdeI.FLAG.for	GCGCCATATG*GATTATAAAGACGACGATGATAAA*CCGATCAATCTCCCCGAGCT
TssA.BglII.rev	GCGCAGATCTTGCGTTTACGACTGCTCGTC
tssC.NcoI.for	GCGCCCATGGCAAACCAGCAAACGGCTGC
tssC.VSVg.BamHI.rev	GCGCGGATCCTTA*CTTACCCAGGCGGTTCATTTCGATATCAGTGTA*TGAATTTGCCGGTTTGG
tssD.forpACYC.NtermVSVgTag	GCGCCCATGGCA*TACACTGATATCGAAATGAACCGCCTGGGTAAG*ATGTTACATATGCACTTGCAGTTTGGTAGT
tssD. BamHI.rev	GCGCGGATCCTTAGACCGCGTAGGTCTTGT

pMALtssEfor	GCGCCATATGAAACGATTCGAACCCAG
pMALtssErev	GCGCGGATCCCGGCTTGATTTACTGCATGC
tssF.NcoI.for	GCGCCCATGGAAGAATTGCTGCCGTATTA
tssF.HA.HindIII.rev	GCGCAAGCTTCTA*AGCGTAGTCTGGGACGTCGTATGGGTA*CGCCAGGATCGATTCGC
tssF.NdeI.HA.for	GCGCCATATG*TACCCATACGACGTCCCAGACTACGCT*GAAGAATTGCTGCCGTATTA
C-term-iotFrev	GCGCGGTACCATCGACTACGCCAGGATCGA
tssI.BspHI.VSVgtag.For	GCGCTCATGAGC*TACACTGATATCGAAATGAACCGCCTGGGTAAG*ATGGATGCGCACAGCATGAT
BCAM0148gp5.BglII.rev	GCGCAGATCTTTAAAACTCCTGGGACCGGTAGC
TssKforpET.NtermVSVgTag	GCGCCCATGGCA*TACACTGATATCGAAATGAACCGCCTGGGTAAG*ATGAGTTATTCGGCCAAGGT
TssK.BglII.rev	GCGCAGATCTTCATGATGTGACCGCGATCA
TssLforpET.NtermVSVgTag	GCGCCCATGGCA*TACACTGATATCGAAATGAACCGCCTGGGTAAG*ATGAGCTACGCGCCTTCCTT
TssL.Rev	GCGCAGATCTTTACTTCAAGCGGTGCGCGAT
TssM1.VSVg.NcoI.for	GCGCCCATGGCA*TACACTGATATCGAAATGAACCGCCTGGGTAAG*GTGAAGCGTGCGAACCAGCA
TssM1.HindIII.rev	GCGCAAGCTTTTAGAAGGTCGCGTAGCGCAGCC
**Two-hybrid system**	
TssA1[B]	
Cterm-iotAfor	GCGCCTGCAGGGATGCCGATCAATCTCCCCGA
Cterm-iotArev	GCGCTCTAGATGCGTTTACGACTGCTCGTC
Nterm-iotAfor	GCGCAAGCTTGATGCCGATCAATCTCCCCGA
N-iotAfullrev	GCGCGGATCCGACGACTGCTCGTCGGGCCG
C-iotAlongNTDrev	GCGCTCTAGAGGTTAGCTGCCGGTATAGCCCTGTT
N-iotAlongNTDrev	GCGCGGATCCTCGCTGCCGGTATAGCCCTGTT
C-term-iotAfor	GCGCCTGCAGGGATGCCGATCAATCTCCCCGA
C-iotAshortCTDfor	GCGCCTGCAGGTCCTCCGGTGACGCAGACGAT
N-iotAshortCTDfor	GCGCAAGCTTTCCTCCGGTGACGCAGACGAT
TssA2[A]	
AHA1844fullfor	GCGCTCTAGAGATGAGCTATCAACACCCCT
AHA1844-Crev2	GCGCGGTACCGGCATTCATTTCGACAACGG
AHA1844-Nrev	GCGCGGTACCCATTTCGACAACGGCGCCGC

AHA1844NTDrev	GCGCGGTACCTTAGCTGGAACTGTCGACGTCGACG
AHA1844NTDrev2	GCGCGGTACCGTGGAACTGTCGACGTCGACG
AHA1844for	GCGCTCTAGACGTCGACGTCGACAGTTCCA
AHA1844CTDrev	GCGCGGTACCTTAGGCCTCGCCAATGCCCGCACT
AHA1844for2	GCGCTCTAGAAAGTGCGGGCATTGGCGAGGC
Other T6SS subunits	
Cterm-iotBfor	GCGCTCTAGACGTCGAGATGGCCAAGAAAGA
Cterm-iotBrev	GCGCGAATTCTTCGTCATTCGCCGTCTTTGT
Nterm-iotBfor	GCGCGCATGCCACCGCAACTGACGCGATAAC
N-iotBfullrev	GCGCTCTAGAGATTCGCCGTCTTTGTCG
Cterm-iotCfor	GCGCCTGCAGAGATGAACCAGCAAACGGCT
Cterm-iotCrev	GCGCTCTAGACTCGCTTATGAATTTGCCGG
Nterm-iotCfor	GCGCAAGCTTGCAGATGAACCAGCAAACGGCT
Nterm-tssCrev.new	GCGCGGATCCTTGAATTTGCCGGTTTGGGCA
Cterm-tssCfor.new	GCGCCTGCAGAATGAACCAGCAAACGGCT
pUT18C-iotDfor	GCGCCTGCAGGAGTCGAAGGGCCATGTTACA
Cterm-iotDrev	GCGCTCTAGAGTTTAGACCGCGTAGGTCTTGT
Nterm-iotDfor	GCGCAAGCTTGCACTTGCAGTTTGGTAGTCC
Nterm-iotDrev	GCGCGGATCCGCGTAGGTCTTGTCGTTCTT
Cterm-iotEfor	GCGCTCTAGAGGCGATGAAACGATTCGAACCC
Cterm-iotErev	GCGCGAATTCTCGGCTTGATTTACTGCATGC
Nterm-iotEfor	GCGCAAGCTTGACGATGAAACGATTCGAACCC
N-iotEfullrev	GCGCGGATCCGACTGCATGCGTGC CGC GCG T
Cterm-iotFfor	GCGCTCTAGAGACGATGGAAGAATTGCTGCCG
Cterm-iotFrev	GCGCGGTACCATCGACTACGCCAGGATCGAT
Nterm-iotFfor	GCGCAAGCTTGCAGATGGAAGAATTGCTGCCGT
N-iotFfullrev	GCGCTCTAGAGACGCCAGGATCGATTCGCC
C-iotGfrag1for	GCGCCTCTAGAGATGCAAGCCCCGAACCGGCG
Cterm-iotGrev	GCGCGAATTCTTGGGTCTCAGTGAATCACGTG
N-iotGfrag1for	GCGCAAGCTTGATGCAAGCCCCGAACCGGCG
Nterm-iotGrev2	GCGCGGATCCTCGTGAATCACGTGCAGTTCGTA
TssIfor	GCGCTCTAGAGATGGATGCGCACAGCATGAT
C-tssIrev	GCGCCCCGGGAGATCTAGTCTATGCTTCCGACGAAG
N-tssIrev	GCGCCCCGGGAAAGATCTCCTGCTTCCGACGAAGGACTGA
Rev.gp5	GCGCAGATCTTTAAAACTCCTGGGACCGGTAG
For1tssj	GCGCCTGCAGAACTTCTGGCCGGATGCGCGGC

C-tssJrev	GCGC<u>GGATCC</u>TTAACCGCAGGAGACCGAAG
For2tssj	GCGC<u>CTGCAG</u>ACTTCTGGCCGGATGCGCGGC
N-tssJrev	GCGC<u>GGATCC</u>AAACCGCAGGAGACCGAAGACA
tssKfor	GCGC<u>GGATCC</u>AATGAGTTATTCGGCCAAGGT
C-tssKrev	GCGC<u>GGTACC</u>TCATGATGTGACCGCGATCA
N-tssKrev	<u>GGTACC</u>AATGATGTGACCGCGATCAGTT
tssLfor	CGCG<u>TCTAGA</u>AATGAGCTACGCGCCTTCCTT
C-tssLrev	GCGC<u>GGTACC</u>TTACTTCAAGCGGTGCGCGATCT
N-tssLrev	GCGC<u>GGTACC</u>ACTTCAAGCGGTGCGCGATCT
tssM1for	CGC<u>TCTAGA</u>AGTGAAGCGTGCGAACCAGCA
C-tssM1rev	CGC<u>GGTACC</u>TTAGAAGAAGGTCGCGTAGCGCA
N-tssM1rev	CGC<u>GGTACC</u>AAAAGAAGGTCGCGTAGCGCAG
tssM2for	CGC<u>GGATCC</u>CACGATCGGCAACCAGCAGCT
C-tssM2rev	GCG<u>GGTACC</u>GTCATTGCGCCCTCTGTGCA
N-tssM2rev	GCG<u>GGTACC</u>AATTGCGCCCTCTGTGCATTCG

[a]Sequences specifying restriction endonuclease cleavage sites are underlined.

[b]Sequences specifying affinity or epitope tags are shown in italic font.

[c]Stop codons are shown in red font.

Supplementary Table 10. Plasmids

Plasmid	Description[a]	Source or reference
pBBR1MCS	Mobilisable BHR cloning vector (Cm^R)	(11)
pBBR1MCS-TssA1^B	Complementation plasmid. Constructed by amplifying *B. cenocepacia tssA* with primers iotAfor and iotArev, restricting with *Hin*dIII and *Bam*HI, and ligating the amplicon to the same sites of pBBR1MCS.	This study
pBBR1MCS-'tssA1^B	The final 347 codons of *B. cenocepacia tssA* were amplified with primers iotAfor3 and iotArev, the amplicon was cut with *Hin*dIII and *Bam*HI, and ligated between the same sites of pBBR1MCS.	This study
p34E-Tp	*dfrB2* (Tp^R) cassette vector (Ap^R, Tp^R)	(12)
pBBR1MCS-'tssA1^B::Tp	pBBR1MCS-'tssA3 with Tp^R cassette from p34E-Tp inserted into the *Eco*RI site within the *tssA* coding sequence.	This study
pSHAFT2	R6K-derived suicide vector for allelic replacement (Ap^R, Cm^R)	(13)
pSHAFT2-'tssA1^B::Tp	Disrupted *tssA1^B* allele was removed from pBBR1MCS-'tssA1^B::Tp with *Xho*I and *Not*I, and ligated to the *Sal*I and *Not*I sites of pSHAFT2.	This study
p34E-TpTer	p34E containing *dfrB2* gene fused to *rrnB* T1T2 terminators (Ap^R, Tp^R)	(13)
pEX18Tp-pheS	Gene replacement vector with ColE1-derived replicon, and mutated phenylalanyl tRNA synthase α-subunit gene, *pheS**, for cPhe counter- selection (Tp^R)	(14)
pEX18Tp-ΔrrnB-pheS	pEX18Tp-pheS derivative with *Afe*I-*Bsa*I restriction fragment removed (Tp^R)	This study
pSNUFF	pEX18Tp-ΔrrnB-pheS derivative containing *dfrB2-rrnB* T1T2 cassette in replacement of *Spe*I-*Eco*RV restriction fragment (Tp^R)	This study
pBBR1-tssM(-)	pBBR1MCS containing *tssM* amplified as a 3,943 bp DNA fragment with primers tssMfor and	This study

	tssMrev2, and ligated between the *Xba*I and *Kpn*I sites	
pBBR1-ΔtssM	pBBR1-tssM(-) with a 2133 bp in-frame deletion introduced into the *tssM* ORF by restriction with *Xho*I followed by religation	This study
pSNUFF-ΔtssM	pSNUFF containing Δ*tssM* allele from pBBR1-ΔtssM ligated between the *Xba*I and *Kpn*I sites	This study
**Protein expression**		
pET14b	T7 promoter expression vector allowing for incorporation of N-terminal His_6 tag, pMB1 origin (Ap^R)	Novagen
pET-21a	T7 promoter expression plasmid for incorporation of C-terminal His_6 tag, pMB1 origin (Ap^R)	Novagen
pACYCDuet-1	Dual T7-lac promoter expression vector, *lacI*, p15A origin (Cm^R)	Novagen
pETDuet-1	Dual T7-lac promoter expression vector, *lacI*, pMB1 origin (Ap^R)	Novagen
pETDuetΔO	pETDuet-1 lacking the *lac* operators at the two T7 promoters and containing a partial deletion of the *lacI* gene. Deletion of *lacO* at first T7 promoter and part of the *lacI* gene involved replacement of the 572 bp region between the *Bcl*I-*Not*I sites by annealed oligonucleotides delacO1for, delacO1for2, delacO1rev and delacO1rev2. Deletion of the *lacO* at the downstream T7 promoter was carried out by replacing the 101 bp region between the *Bsr*GI-*Nde*I sites by annealed oligonucleotides delacO2for, delacO2for2, delacO2rev and delacO2rev2. For both manipulations, the 'for2' and 'rev2' primers were kinased to facilitate ligation of the assembled double-stranded oligonucleotide to the vector.	This study
pMAL-c5X	Plasmid for generating fusions of MalE (MBP) to the N-terminus of target proteins. *malE* is under P_{tac} control. Contains *lacI* under P_{lacIQ} control (Ap^R)	New England Bio-labs

pMAL-c5X-His_6	pMAL-c5X encoding MBP with a hexa-histidine tag located between amino acids 3 and 4. Constructed by replacing the 390 bp *Mfe*I-*Bgl*II fragment (that includes the *malE* Shine-Dalgarno sequence and first ~360 bp of *malE*) with a *Mfe*I-*Bgl*II DNA fragment generated by PCR using pMAL-c5X as template and primers pMALHisFor and pMALHisRev.	This study
pET14b-$TssA1^B$	pET14b encoding native $TssA1^B$. *B. cenocepacia tssA* was amplified with primers tssAnonHisFor and pET14b-iotArev, cut with *Xba*I and *Bam*HI, and ligated between the same sites of pET14b	This study
pET14b-His_6.$TssA1^B$	pET14b encoding N-terminal His-tagged $TssA1^B$. *B. cenocepacia tssA* was amplified with primers pET14b-iotAfor and pET14b-iotArev, cut with *Nde*I and *Bam*HI, and ligated between the same sites of pET14b. The encoded product contains a 20 amino acid N-terminal tag that harbours a hexahistidine tag and a thrombin cleavage site.	This study
pACYCDuet-linkerHis1.$TssA1^B$	pACYCDuet-1 encoding *B. cenocepacia* TssA with 6 consecutive histidine codons located between codons 262-263. Constructed by SOE PCR using two pairs of primers (pET14b-iotAfor and TssAlinkerHis1rev, and TssAlinkerHis1for and pET14b-iotArev) in which the products from the first pair of PCRs were combined and spliced in a second PCR using pET14b-iotAfor and pET14b-iotArev. The resulting amplicon was cut with *Nde*I and *Bgl*II, and ligated to the same sites of pACYCDuet-1.	This study
pMAL-c5X-$TssA1^B$.CTD	pMAL-c5X encoding a MBP-$TssA1^B$ CTD fusion. DNA encoding amino acids 294-373 of *B. cenocepacia tssA* was amplified with primers pET14b-ACTDfor and pET14b-iotArev, the product was restricted with *Nde*I and *Bam*HI, and ligated between the same sites of pMAL-c5X.	This study

	Following cleavage of the MBP fusion with Factor Xa, the released $TssA_{CTD}$ contains four additional non-native amino acids (ISHM) at the N-terminus.	
pMAL-c5X-TssA1^{B}.CTD3	pMAL-c5X encoding MBP fused to amino acids 303-373 of *B. cenocepacia* TssA1^{B}.	(2)
pMAL-c5X-His_6.TssA1^{B}.CTD H12	pMAL-c5X encoding MBP fused to His_6-tagged amino acids 303-329 of Bc TssA1^{B} (includes α-helix H12). *B. cenocepacia* DNA was amplified with primers pACYC-tssA.CTDfor and pACYC-tssA.CTDH1rev2, the product was restricted with *Nde*I and *Bam*HI, and ligated between the same sites of pMAL-c5X.	This study
pMAL-c5X-His_6.TssA1^{B}.CTD H12-H13	pMAL-c5X encoding MBP fused to His_6-tagged amino acids 303-347 of Bc TssA1^{B} (includes α-helices H12-H13). *B. cenocepacia* DNA was amplified with primers pACYC-tssA.CTDfor and pACYC-tssA.CTDH2rev2, the product was restricted with *Nde*I and *Bam*HI, and ligated between the same sites of pMAL-c5X.	This study
pMAL-c5X-His_6.TssA1^{B}.CTD H12-H14	pMAL-c5X encoding MBP fused to His_6-tagged amino acids 303-358 of Bc TssA1^{B} (includes α-helices H12-H14). *B. cenocepacia* DNA was amplified with primers pACYC-tssA.CTDfor and pACYC-tssA.CTDH3rev2, the product was restricted with *Nde*I and *Bam*HI, and ligated between the same sites of pMAL-c5X.	This study
pMAL-c5X-His_6.TssA1^{B}.CTD	pMAL-c5X encoding MBP fused to His_6-tagged amino acids 303-373 of Bc TssA1^{B}, i.e. the entire CTD (includes H12-H15). *B. cenocepacia* DNA was amplified with primers pACYC-tssA.CTDfor and pET14b-iotArev, the product was restricted with *Nde*I and *Bam*HI, and ligated between the same sites of pMAL-c5X.	This study
pET21a-TssA2^{A}.Nt1-Nt2.His_6	pET21a encoding Nt1-Nt2 region of TssA2^{A} with His-tag at C-terminus of Nt2. *A. hydrophila tssA* codons (1-374) amplified with primers pET21a-	This study

	AHA1844.Nt1Nt2.for and pET21a-AHA1844.Nt1Nt2.rev, and ligated between the *Nde*I and *Xho*I sites of pET21a	
pACYCDuet-His$_6$.TssA2^A.CTD	pACYCDuet-1 encoding N-terminal His-tagged TssA2^A CTD. *A. hydrophila tssA* codons 381-478 amplified with primers pACYC-AHA1844.CTDSD2for and pACYC-AHA1844.rev, and ligated between the *Bam*HI and *Hin*dIII sites of pACYCDuet-1	This study
pET14b-His$_6$.TssD2	pET14b encoding N-terminal His-tagged TssD. *B. cenocepacia tssD* amplified with primers pET-His-iotDfor2 and pET14b-iotDrev, cut with *Nde*I and *Bam*HI, and ligated to the same sites of pET14b. Expresses TssD lacking the first three amino acids of TssD and containing a 20 amino acid N-terminal tag that harbours a hexahistidine tag and a thrombin cleavage site.	
**Protein expression for co-IP**		
pACYCDuet-FLAG.tssA	*B. cenocepacia tssA* amplified with primers TssA.NdeI.FLAG.for and TssA.BglII.rev, and inserted between *Nde*I and *Bgl*II sites of pACYCDuet-1. Expresses $_{FLAG}$.TssA1^B.	This study
pACYCDuet-tssC.VSVg	*B. cenocepacia tssC* amplified with primers tssC.NcoI.for and tssC.VSVg.BamHI.rev, and inserted between *Nco*I and *Bam*HI sites of pACYCDuet-1. Expresses TssC.$_{VSVg}$.	This study
pACYCDuet-tssC.VSVg-FLAG.tssA	*B. cenocepacia tssC* amplified with primers tssC.NcoI.for and tssC.VSVg.BamHI.rev, and inserted between *Nco*I and *Bam*HI sites of pACYCDuet-FLAG.tssA. Expresses TssC.$_{VSVg}$ and $_{FLAG}$.TssA1^B.	This study
pACYCDuet-VSVg.tssD	*B. cenocepacia tssD* amplified with tssD.forpACYC.NtermVSVgTag and tssD. BamHI.rev, and inserted between *Nco*I and	This study

	*Bam*HI sites of pACYCDuet-1. Expresses $_{VSVg}$.TssD.	
pACYCDuet-VSVg.tssD-FLAG.tssA	*B. cenocepacia tssD* amplified with tssD.forpACYC.NtermVSVgTag and tssD. BamHI.rev, and inserted between *Nco*I and *Bam*HI sites of pACYCDuet-FLAG.tssA. Expresses $_{VSVg}$.TssD and $_{FLAG}$.TssA1^{B}.	This study
pMAL-c5X-His$_6$.tssE	*B. cenocepacia tssE* amplified with pMALtssEfor and pMALtssErev, and inserted between *Nde*I and *Bam*HI sites of pMAL-c5X-His$_6$. Expresses $_{His6}$.MBP-TssE.	This study
pACYCDuet-tssF.HA	*B. cenocepacia tssF* amplified with tssF.NcoI.for and tssF.HA.HindIII.rev, and inserted between *Nco*I and *Hin*dIII sites of pACYCDuet-1. Expresses TssF$_{.HA}$.	This study
pACYCDuet-tssF.HA-FLAG.tssA	*B. cenocepacia tssF* amplified with tssF.NcoI.for and tssF.HA.HindIII.rev, and inserted between *Nco*I and *Hin*dIII sites of pACYCDuet-FLAG.TssA. Expresses TssF$_{.HA}$ and $_{FLAG}$.TssA1^{B}.	This study
pETDuet∆O-HA.tssF	*B. cenocepacia tssF* amplified with tssF.NdeI.HA.for and C-term-iotFrev, and inserted between *Nde*I and *Acc*65I sites of pETDuet∆O. Expresses $_{HA}$.TssF.	This study
pACYCDuet-VSVg.tssI$_{gp27gp5}$	*B. cenocepacia tssI* conserved core region amplified with tssI.BspHI.VSVgtag.For and BCAM0148gp5.BglII.rev, and inserted between *Nco*I and *Bam*HI sites of pACYCDuet-1. Expresses $_{VSVg}$.TssI$_{C}$ corresponding to the gp27gp5 core region (N-terminal 526 amino acids).	This study
pACYCDuet-VSVg.tssI$_{gp27gp5}$-FLAG.tssA	*B. cenocepacia tssA* amplified with TssA.NdeI.FLAG.for and TssA.BglII.rev, and inserted between *Nde*I and *Bgl*II sites of pACYCDuet-VSVg. tssI$_{gp27gp5}$. Expresses $_{VSVg}$.TssI$_{C}$ and $_{FLAG}$.TssA1^{B}.	This study

pACYCDuet-VSVg.tssK	*B. cenocepacia tssK* amplified with TssKforpET.NtermVSVgTag and TssK.BglII.rev, and inserted between *Nco*I and *Bam*HI sites of pACYCDuet-1. Expresses $_{VSVg}$.TssK.	This study
pACYCDuet-VSVg.tssK-FLAG.tssA	*B. cenocepacia tssA* amplified with TssA.NdeI.FLAG.for and TssA.BglII.rev, and inserted between *Nde*I and *Bgl*II sites of pACYCDuet-VSVg.tssK. Expresses $_{VSVg}$.TssK and $_{FLAG}$.TssA1[B].	This study
pACYCDuet-VSVg.tssL	*B. cenocepacia tssL* amplified with TssLforpET.NtermVSVgTag and TssL.Rev, and inserted between *Nco*I and *Bam*HI sites of pACYCDuet-1. Expresses $_{VSVg}$.TssL.	This study
pACYCDuet-VSVg.tssL-FLAG.tssA	*B. cenocepacia tssL* amplified with TssLforpET.NtermVSVgTag and TssL.Rev, and inserted between *Nco*I and *Bam*HI sites of pACYCDuet-FLAG.TssA. Expresses $_{VSVg}$.TssL and $_{FLAG}$.TssA1[B].	This study
pACYCDuet-VSVg.tssM$_{NTR}$	*B. cenocepacia tssM* codons 62-449 amplified with TssM1.VSVg.NcoI.for and TssM1.HindIII.rev, and inserted between *Nco*I and *Hin*dIII sites of pACYCDuet-1. Expresses $_{VSVg}$.TssM$_{NTR}$ (TssM cytoplasmic component).	This study
pACYCDuet-VSVg.tssM$_{NTR}$-FLAG.tssA	*B. cenocepacia tssM* codons 62-449 amplified with TssM1.VSVg.NcoI.for and TssM1.HindIII.rev, and inserted between *Nco*I and *Hin*dIII sites of pACYCDuet-FLAG.tssA. Expresses $_{VSVg}$.TssM$_{NTR}$ and $_{FLAG}$.TssA1[B].	This study
**Two-hybrid system[b]**		
pKT25	Vector for generating fusions to C-terminus of CyaA T25 fragment, *ori*$_{p15A}$ (Km^R)	(15)
pKT25-zip	pKT25 containing the coding sequence of the yeast GCN4 leucine zipper fused to the T25 coding sequence	(9)
pKNT25	Vector for generating fusions to N-terminus of CyaA T25 fragment, *ori*$_{p15A}$ (Km^R)	(16)

pUT18	Vector for generating fusions to N-terminus of CyaA T18 fragment, *ori*$_{pMB1}$ (Ap^R)	(15)
pUT18C	Vector for generating fusions to C-terminus of CyaA T18 fragment, *ori*$_{pMB1}$ (Ap^R)	(15)
pUT18C-zip	pUT18C containing the coding sequence of the yeast GCN4 leucine zipper fused to the T18 coding sequence	(15)
pKT25-tssA1^B	*B. cenocepacia tssA* amplified with Cterm-iotAfor and Cterm-iotArev, and inserted between the *Pst*I and *Xba*I sites of pKT25.	This study
pKNT25-tssA1^B	*B. cenocepacia tssA* amplified with Nterm-iotAfor and N-iotAfullrev, and inserted between the *Hin*dIII and *Bam*HI sites of pKNT25	This study
pUT18-tssA1^B	*B. cenocepacia tssA* amplified with Nterm-iotAfor and N-iotAfullrev, and inserted between the *Hin*dIII and *Bam*HI sites of pUT18	This study
pUT18C-tssA1^B	*B. cenocepacia tssA* amplified with Cterm-iotAfor and Cterm-iotArev, and inserted between the *Pst*I and *Xba*I sites of pUT18C	This study
pKT25-tssA1$^B_{Nt1}$	*B. cenocepacia tssA* codons 1-256 amplified with C-term-iotAfor and C-iotAlongNTDrev, and inserted between the *Pst*I and *Xba*I sites of pKT25	This study
pKNT25-tssA1$^B_{Nt1}$	*B. cenocepacia tssA* codons 1-256 amplified with Nterm-iotAfor and N-iotAlongNTDrev, and inserted between the *Hin*dIII and *Bam*HI sites of pKNT25	This study
pUT18-tssA1$^B_{Nt1}$	*B. cenocepacia tssA* codons 1-256 amplified with Nterm-iotAfor and N-iotAlongNTDrev, and inserted between the *Hin*dIII and *Bam*HI sites of pUT18	This study
pUT18C-tssA1$^B_{Nt1}$	*B. cenocepacia tssA* codons 1-256 amplified with C-term-iotAfor and C-iotAlongNTDrev, and inserted between the *Pst*I and *Xba*I sites of pUT18C	This study

pKT25-tssA1$^{B}_{CTD}$	*B. cenocepacia tssA* codons 294-374 amplified with C-iotAshortCTDfor and Cterm-iotArev, and inserted between the *Pst*I and *Xba*I sites of pKT25	This study
pKNT25-tssA1$^{B}_{CTD}$	*B. cenocepacia tssA* codons 294-373 amplified with N-iotAshortCTDfor and N-iotAfullrev, and inserted between the *Hin*dIII and *Bam*HI sites of pKNT25	This study
pUT18-tssA1$^{B}_{CTD}$	*B. cenocepacia tssA* codons 294-373 amplified with N-iotAshortCTDfor and N-iotAfullrev, and inserted between the *Hin*dIII and *Bam*HI sites of pUT18	This study
pUT18C-tssA1$^{B}_{CTD}$	*B. cenocepacia tssA* codons 294-374 amplified with C-iotAshortCTDfor and Cterm-iotArev, and inserted between the *Pst*I and *Xba*I sites of pUT18C	This study
pKT25-tssA2^{A}	*A. hydrophila tssA* amplified with primers AHA1844fullfor and AHA1844-Crev2, and inserted between the *Xba*I and *Kpn*I sites of pKT25.	This study
pKNT25-tssA2^{A}	*A. hydrophila tssA* amplified with primers AHA1844fullfor and AHA1844-Nrev, and inserted between the *Xba*I and *Kpn*I sites of pKNT25	This study
pUT18-tssA2^{A}	*A. hydrophila tssA* amplified with primers AHA1844fullfor and AHA1844-Nrev, and inserted between the *Xba*I and *Kpn*I sites of pUT18	This study
pUT18C-tssA2^{A}	*A. hydrophila tssA* amplified with primers AHA1844fullfor and AHA1844-Crev2, and inserted between the *Xba*I and *Kpn*I sites of pUT18C.	This study
pKT25-tssA2^{A} Nt1	*A. hydrophila tssA* codons 1-229 amplified with primers AHA1844fullfor and AHA1844NTDrev, and inserted between the *Xba*I and *Kpn*I sites of pKT25	This study
pKNT25-tssA2^{A} Nt1	*A. hydrophila tssA* codons 1-229 amplified with primers AHA1844fullfor and AHA1844NTDrev2,	This study

	and inserted between the *Xba*I and *Kpn*I sites of pKNT25	
pUT18-tssA2^{A} Nt1	*A. hydrophila tssA* codons 1-229 amplified with primers AHA1844fullfor and AHA1844NTDrev2, and inserted between the *Xba*I and *Kpn*I sites of pUT18	This study
pUT18C-tssA2^{A} Nt1	*A. hydrophila tssA* codons 1-229 amplified with primers AHA1844fullfor and AHA1844NTDrev, and inserted between the *Xba*I and *Kpn*I sites of pUT18C	This study
pKT25-tssA2^{A} Nt2	*A. hydrophila tssA* codons 244-386 amplified with primers AHA1844for and AHA1844CTDrev, and inserted between the *Xba*I and *Kpn*I sites of pKT25	This study
pUT18C-tssA2^{A} Nt2	*A. hydrophila tssA* codons 244-386 amplified with primers AHA1844for and AHA1844CTDrev, and inserted between the *Xba*I and *Kpn*I sites of pUT18C	This study
pKT25-tssA2^{A} CTD	*A. hydrophila tssA* codons 381-478 amplified with primers AHA1844for2 and AHA1844-Crev2, and inserted between the *Xba*I and *Kpn*I sites of pKT25	This study
pUT18C-tssA2^{A} CTD	*A. hydrophila tssA* codons 381-478 amplified with primers AHA1844for2 and AHA1844-Crev2, and inserted between the *Xba*I and *Kpn*I sites of pUT18C	This study
pKT25-tssA2^{A} Nt2-CTD	*A. hydrophila tssA* codons 244-478 amplified with primers AHA1844for and AHA1844-Crev2, and inserted between the *Xba*I and *Kpn*I sites of pKT25	This study
pUT18C-tssA2^{A} Nt2-CTD	*A. hydrophila tssA* codons 244-478 amplified with primers AHA1844for and AHA1844-Crev2, and inserted between the *Xba*I and *Kpn*I sites of pUT18C	This study

pKT25-tssB	*B. cenocepacia tssB* amplified with Cterm-iotBfor and Cterm-iotBrev, and inserted between the *XbaI* and *Eco*RI sites of pKT25	This study
pKNT25-tssB	*B. cenocepacia tssB* amplified with Nterm-iotBfor and N-iotBfullrev, and inserted between the *SphI* and *XbaI* sites of pKNT25	This study
pUT18-tssB	*B. cenocepacia tssB* amplified with Nterm-iotBfor and N-iotBfullrev, and inserted between *SphI* and *XbaI* sites of pUT18	This study
pUT18C-tssB	*B. cenocepacia tssB* amplified with Cterm-iotBfor and Cterm-iotBrev, and inserted between the *XbaI* and *Eco*RI sites of pUT18C	This study
pKT25-tssC	*B. cenocepacia tssC* amplified with Cterm-iotCfor and Cterm-iotCrev, and inserted between the *Pst*I and *Xba*I sites of pKT25	This study
pKNT25-tssC	*B. cenocepacia tssC* amplified with Nterm-iotCfor and Nterm-tssCrev.new, and inserted between the *Hin*dIII and *Bam*HI sites of pKNT25, followed by *Acc*65I digestion, end filling and self-ligation.	This study
pUT18-tssC	*B. cenocepacia tssC* amplified with Nterm-iotCfor and Nterm-tssCrev.new, and inserted between *Hin*dIII and *Bam*HI sites of pUT18	This study
pUT18C-tssC	*B. cenocepacia tssC* amplified with Cterm-tssCfor.new and Cterm-iotCrev, and inserted between the *Pst*I and *Xba*I sites of pUT18C	This study
pKT25-tssD	*B. cenocepacia tssD* amplified with pUT18C-iotDfor and Cterm-iotDrev, and inserted between the *Pst*I and *Xba*I sites of pKT25	This study
pKNT25-tssD	*B. cenocepacia tssD* amplified with Nterm-iotDfor and Nterm-iotDrev, and inserted between the *Hin*dIII and *Bam*HI sites of pKNT25	This study
pUT18-tssD	*B. cenocepacia tssD* amplified with Nterm-iotDfor and Nterm-iotDrev, and inserted between *Hin*dIII and *Bam*HI sites of pUT18	This study

pUT18C-tssD	*B. cenocepacia tssD* amplified with pUT18C-iotDfor and Cterm-iotDrev, and inserted between the *Pst*I and *Xba*I sites of pUT18C	This study
pKT25-tssE	*B. cenocepacia tssE* amplified with Cterm-iotEfor and Cterm-iotErev, and inserted between the *Xba*I and *Eco*RI sites of pKT25	This study
pKNT25-tssE	*B. cenocepacia tssE* amplified with Nterm-iotEfor and N-iotEfullrev, and inserted between the *Hin*dIII and *Bam*HI sites of pKNT25	This study
pUT18-tssE	*B. cenocepacia tssE* amplified with Nterm-iotEfor and N-iotEfullrev, and inserted between *Hin*dIII and *Bam*HI sites of pUT18	This study
pUT18C-tssE	*B. cenocepacia tssE* amplified with Cterm-iotEfor and Cterm-iotErev, and inserted between the *Xba*I and *Eco*RI sites of pUT18C	This study
pKT25-tssF	*B. cenocepacia tssF* amplified with Cterm-iotFfor and Cterm-iotFrev, and inserted between the *Xba*I and *Acc*65I sites of pKT25	This study
pKNT25-tssF	*B. cenocepacia tssF* amplified with Nterm-iotFfor and N-iotFfullrev, and inserted between the *Hin*dIII and *Xba*I sites of pKNT25	This study
pUT18-tssF	*B. cenocepacia tssF* amplified with Nterm-iotFfor and N-iotFfullrev, and inserted between *Hin*dIII and *Xba*I sites of pUT18	This study
pUT18C-tssF	*B. cenocepacia tssF* amplified with Cterm-iotFfor and Cterm-iotFrev, and inserted between the *Xba*I and *Acc*65I sites of pUT18C	This study
pKT25-tssG	*B. cenocepacia tssG* amplified with C-iotGfrag1for and Cterm-iotGrev, and inserted between the *Xba*I and *Eco*RI sites of pKT25	This study
pKNT25-tssG	*B. cenocepacia tssG* amplified with N-iotGfrag1for and Nterm-iotGrev2, and inserted between the *Hin*dIII and *Bam*HI sites of pKNT25	This study
pUT18-tssG	*B. cenocepacia tssG* amplified with N-iotGfrag1for and Nterm-iotGrev2, and inserted between *Hin*dIII and *Bam*HI sites of pUT18	This study

pUT18C-tssG	*B. cenocepacia tssG* amplified with C-iotGfrag1for and Cterm-iotGrev, and inserted between the *Xba*I and *Eco*RI sites of pUT18C	This study
pKT25-tssI	*B. cenocepacia tssI* amplified with TssIfor and C-tssIrev, and inserted between the *Xba*I and *Bam*HI sites of pKT25 following cleavage of the amplicon with *Xba*I and *Bgl*II	This study
pKNT25-tssI	*B. cenocepacia tssI* amplified with TssIfor and N-tssIrev, and inserted between the *Xba*I and *Bam*HI of pKNT25 following cleavage of the amplicon with *Xba*I and *Bgl*II	This study
pUT18-tssI	*B. cenocepacia tssI* amplified with TssIfor and N-tssIrev, and inserted between the *Xba*I and *Bam*HI sites of pUT18 following cleavage of the amplicon with *Xba*I and *Bgl*II	This study
pUT18C-tssI	*B. cenocepacia tssI* amplified with TssIfor and C-tssIrev, and inserted between the *Xba*I and *Bam*HI sites of pUT18C following cleavage of the amplicon with *Xba*I and *Bgl*II	This study
pKT25-tssI$_C$	*B. cenocepacia tssI* codons 1-526 amplified with TssIfor and Rev.gp5, and inserted between the *Xba*I and *Bam*HI sites of pKT25 following cleavage of the amplicon with *Xba*I and *Bgl*II	This study
pUT18C-tssI$_C$	*B. cenocepacia tssI* codons 1-526 amplified with TssIfor and Rev.gp5, and inserted between the *Xba*I and *Bam*HI sites of pUT18C following cleavage of the amplicon with *Xba*I and *Bgl*II	This study
pKT25-tssJ	*B. cenocepacia tssJ* codons 13-200 amplified with For1tssj and C-tssJrev, and inserted between the *Pst*I and *Bam*HI sites of pKT25	This study
pKNT25-tssJ	*B. cenocepacia tssJ* codons 13-200 amplified with For2tssj and N-tssJrev, and inserted between the *Pst*I and *Bam*HI sites of pKNT25	This study
pUT18-tssJ	*B. cenocepacia tssJ* codons 13-200 amplified with For2tssj and N-tssJrev, and inserted between *Pst*I and *Bam*HI sites of pUT18	This study

pUT18C-tssJ	*B. cenocepacia tssJ* codons 13-200 amplified with For2tssj and C-tssJrev, and inserted between the *Pst*I and *Bam*HI sites of pUT18C	This study
pKT25-tssK	*B. cenocepacia tssK* amplified with tssKfor and C-tssKrev, and inserted between the *Bam*HI and *Acc*65I sites of pKT25	This study
pKNT25-tssK	*B. cenocepacia tssK* amplified with tssKfor and N-tssKrev, and inserted between the *Hin*dIII and *Bam*HI sites of pKNT25	This study
pUT18-tssK	*B. cenocepacia tssK* amplified with tssKfor and N-tssKrev, and inserted between *Bam*HI and *Acc*65I sites of pUT18	This study
pUT18C-tssK	*B. cenocepacia tssK* amplified with tssKfor and C-tssKrev, and inserted between the *Bam*HI and *Acc*65I sites of pUT18C	This study
pKT25-tssL	*B. cenocepacia tssL* codons 1-204 amplified with tssLfor and C-tssLrev, and inserted between the *Xba*I and *Acc*65I sites of pKT25	This study
pKNT25-tssL	*B. cenocepacia tssL* codons 1-204 amplified with tssLfor and N-tssLrev, and inserted between the *Xba*I and *Acc*65I sites of pKNT25	This study
pUT18-tssL	*B. cenocepacia tssL* codons 1-204 amplified with tssLfor and N-tssLrev, and inserted between *Xba*I and *Acc*65I sites of pUT18	This study
pUT18C-tssL	*B. cenocepacia tssL* codons 1-204 amplified with tssLfor and C-tssLrev, and inserted between the *Xba*I and *Acc*65I sites of pUT18C	This study
pKT25-tssM$_{NTR}$	*B. cenocepacia tssM* codons 62-450 amplified with tssM1for and C-tssM1rev, and inserted between the *Xba*I and *Acc*65I sites of pKT25	This study
pKNT25-tssM$_{NTR}$	*B. cenocepacia tssM* codons 62-450 amplified with tssM1for and N-tssM1rev, inserted between the *Xba*I and *Acc*65I sites of pKNT25, followed by *Acc*65I digestion, end filling and self-ligation.	This study
pUT18-tssM$_{NTR}$	*B. cenocepacia tssM* codons 62-450 amplified with tssM1for and N-tssM1rev, and inserted	This study

	between *Xba*I and *Acc*65I sites of pUT18, followed by *Acc*65I digestion, DNA end filling and self-ligation.	
pUT18C-tssM$_{NTR}$	*B. cenocepacia tssM* codons 62-450 amplified with tssM1for and C-tssM1rev, and inserted between the *Xba*I and *Acc*65I sites of pUT18C	This study
pKT25-tssM$_{CTR}$	*B. cenocepacia tssM* codons 467-1309 amplified with tssM2for and C-tssM2rev, and inserted between the *Bam*HI and *Acc*65I sites of pKT25	This study
pKNT25-tssM$_{CTR}$	*B. cenocepacia tssM* codons 467-1308 amplified with tssM2for and N-tssM2rev, and inserted between the *Bam*HI and *Acc*65I sites of pKNT25	This study
pUT18-tssM$_{CTR}$	*B. cenocepacia tssM* codons 467-1308 amplified with tssM2for and N-tssM2rev, and inserted between the *Bam*HI and *Acc*65I sites of pUT18	This study
pUT18C-tssM$_{CTR}$	*B. cenocepacia tssM* codons 467-1309 amplified with tssM2for and C-tssM2rev, and inserted between the *Bam*HI and *Acc*65I sites of pUT18C	This study

[a]Ap^R, ampicillin resistance; Cm^R, chloramphenicol resistance; Km^R, kanamycin resistance; Tp^R, trimethoprim resistance; BHR, broad host-range

[b]T6SS subunit or domain coding sequences included the native or an added translation termination codon (as appropriate) when ligated to pKT25 or pUT18C but were omitted for pKNT25 and pUT18.

Supplementary references

1. Sievers, F. & Higgins, D. G. Clustal Omega, accurate alignment of very large numbers of sequences. *Methods Mol. Biol.* **1079,** 105–16 (2014).
2. Owen, H. J. *et al.* TssA from Burkholderia cenocepacia : expression, purification, crystallization and crystallographic analysis. *Acta Crystallogr. Sect. F Struct. Biol. Commun.* **74,** 536–542 (2018).
3. Chen, V. B. *et al.* MolProbity: All-atom structure validation for macromolecular crystallography. *Acta Crystallogr. Sect. D Biol. Crystallogr.* **66,** 12–21 (2010).
4. Romling, U. *et al.* Epidemiology of chronic Pseudomonas aeruginosa infections in cystic fibrosis. *J Infect Dis* **170,** 1616–1621 (1994).
5. Seshadri, R. *et al.* Genome sequence of Aeromonas hydrophila ATCC 7966T: Jack of all trades. *J. Bacteriol.* **188,** 8272–8282 (2006).
6. Yanisch-Perron, C., Vieira, J. & Messing, J. Improved M13 phage cloning vectors and host strains: nucleotide sequences of the M13mpl8 and pUC19 vectors. *Gene* **33,** 103–119 (1985).
7. Jessee, J. New subcloning efficiency competent cells: >1 × 106 transformants/µg. *Focus Bethesda Res. Lab.* **8,** 9–10 (1986).
8. Simon, R., Priefer, U. & Pühler, A. A Broad Host Range Mobilization System for In Vivo Genetic Engineering: Transposon Mutagenesis in Gram Negative Bacteria. *Biotechnology* **1,** 784–791 (1983).
9. Karimova, G., Pidoux, J., Ullmann, A. & Ladant, D. A bacterial two-hybrid system based on a reconstituted signal transduction pathway. *Proc. Natl. Acad. Sci.* **95,** 5752–5756 (1998).
10. Studier, F. W. & Moffatt, B. A. Use of bacteriophage T7 RNA polymerase to direct selective high-level expression of cloned genes. *J. Mol. Biol.* **189,** 113–130 (1986).
11. Kovach, M. E., Phillips, R. W., Elzer, P. H., Roop, R. M. & Peterson, K. M. pBBR1MCS: a broad-host-range cloning vector. *Biotechniques* **16,** 800–802 (1994).
12. DeShazer, D. & Woods, D. E. Broad-Host-Range Cloning and Cassette Vectors

Based on the R388 Trimethoprim Resistance Gene. *Biotechniques* **20,** 762–764 (1996).

13. Shastri, S. *et al.* An efficient system for the generation of marked genetic mutants in members of the genus Burkholderia. *Plasmid* **89,** 49–56 (2017).
14. Barrett, A. R. *et al.* Genetic Tools for Allelic Replacement in Burkholderia Species. *Appl. Environ. Microbiol.* **74,** 4498–4508 (2008).
15. Karimova, G., Ullmann, A. & Ladant, D. Protein-protein interaction between Bacillus stearothermophilus tyrosyl-tRNA synthetase subdomains revealed by a bacterial two-hybrid system. *J. Mol. Microbiol. Biotechnol.* **3,** 73–82 (2001).
16. Karimova, G., Dautin, N. & Ladant, D. Interaction network among Escherichia coli membrane proteins involved in cell division as revealed by bacterial two-hybrid analysis. *J. Bacteriol.* **187,** 2233–2243 (2005).
