# Supplementary material for: Structural insights into the function of type VI secretion system TssA subunits
Source: Nat Commun. 2018 Nov 12;9:4765. doi: 10.1038/s41467-018-07247-1 (PMC6232143; doi:10.1038/s41467-018-07247-1)
Supplement: Supplementary file 2 — Description of Additional Supplementary Files [file 41467_2018_7247_MOESM2_ESM.docx]

**Description of Additional Supplementary Files**

**File Name:** Supplementary Data 1

**Description:** β-galactosidase activity measurements for two-hybrid assays.

Sheet 1 shows the activities presented in Figure 2b. Sheet 2 shows the activities presented

in Supplementary Figure 6b. Plasmid designations: pKT, pKT25; pKNT, pKNT25; pUT, pUT18;

pUTC, pUT18C.
